# Supplementary material for: Cost-effectiveness of a patient-centred approach to managing multimorbidity in primary care: a pragmatic cluster randomised controlled trial
Source: BMJ Open. 2020 Jan 19;10(1):e030110. doi: 10.1136/bmjopen-2019-030110 (PMC7044971; doi:10.1136/bmjopen-2019-030110)
Supplement: Supplementary data [file bmjopen-2019-030110supp001.pdf]

Appendix 1. Unit costs

Service		Unit cost (£)	Notes	Source
**Social services**				
Social home care provided for social services		12	Independent sector home care provided for social services, face-to-face, based on 30 minute visit	[1]
Daycare		61	Per client attendance	
Social worker		55	Based on 1 hour appointment	
Meals on wheels		7.03		[2]
**Prescription charges***				
Prescription charge per item		8.40		[3]
Prescription charge 3mo prepayment certificate		29.10		
Prescription charge 12mo prepayment certificate		104		
**GP practice services**				
GP appointment	Control arm	51.84	Per surgery consultation lasting 14.4 minutes (3D data)	[1]
	Intervention arm	48.60	Per surgery consultation lasting 13.5 minutes (3D data)	

Thorn J, *et al. BMJ Open* 2020; 10:e030110. doi: 10.1136/bmjopen-2019-030110

Service		Unit cost (£)	Notes	Source
GP home visit		100	Based on consultation lasting 15 minutes and 12 minutes travel time[4], plus 5 miles travel cost (NHS travel reimbursed at 56p per mile)	
GP phone call	Control arm	41.40	Based on consultation lasting 11.5 minutes (3D data)	
	Intervention arm	32.40	Based on consultation lasting 9 minutes (3D data)	
GP email consultation		21.60	Based on 6 minute consultation[5]	
Nurse appointment	Control arm	10.85	Based on 15.1 minute appointment (3D data), inflated from 14/15 costs[6]	
	Intervention arm	14.16	Based on 19.7 minute appointment (3D data), inflated from 14/15 costs[6]	
Nurse phone call	Control arm	4.24	Based on 5.9 minute call, inflated from 14/15 costs [6]	
	Intervention arm	5.03	Based on 7 minute call, inflated from 14/15 costs[6]	
Nurse home visit		22.21	Based on 15 minute visit, 12 minutes travelling, 5 miles travel costs (NHS travel reimbursed at 56p per mile)	
HCA face-to-face appointment	Control arm	3.45	Based on 9.4 minute appointment (3D data), band[**] 2 nurse	
	Intervention arm	3.59	Based on 9.8 minute appointment (3D data), band 2 nurse	
HCA phone call (control arm)		5.87	Based on 16 minute call (3D data), band 2 nurse	

Thorn J, *et al. BMJ Open* 2020; 10:e030110. doi: 10.1136/bmjopen-2019-030110

Service	Unit cost (£)	Notes	Source
HCA home visit	12.70	Based on 15 minutes appointment, 12 minutes travelling, 5 miles travel costs, band 2 nurse (NHS travel reimbursed at 56p per mile)	
**Community services**			
Occupational Therapy visit	16	Based on band 5 occupational therapist, 30 minute appointment	[1]
Occupational Therapy home visit	25.20	Based on band 5 occupational therapist, 30 minute appointment, 12 minutes travelling, 5 miles travel costs (NHS travel reimbursed at 56p per mile)	
Occupational Therapy phone call	5.33	Based on band 5 occupational therapist, 10 minute call	
Speech and Language Therapy (SALT) visit	16	Based on band 5 SALT, 30 minute appointment	
Speech and Language Therapy home visit	25.20	Based on band 5 SALT, 30 minute appointment, 12 minutes travelling, 5 miles travel costs (NHS travel reimbursed at 56p per mile)	
Speech and Language Therapy telephone call	5.33	Based on band 5 SALT, 10 minute call	

Thorn J, *et al. BMJ Open* 2020; 10:e030110. doi: 10.1136/bmjopen-2019-030110

Service	Unit cost (£)	Notes	Source
Physiotherapy visit	16	Based on band 5 physiotherapist, 30 minute appointment	
Physiotherapy home visit	25.20	Based on band 5 physiotherapist, 30 minute appointment, 12 minutes travelling, 5 miles travel costs (NHS travel reimbursed at 56p per mile)	
Physiotherapy phone call	5.33	Based on band 5 physiotherapist, 10 minute call	
Chiropody / Podiatry visit	16	Based on band 5 podiatrist, 30 minute appointment	
Chiropody / Podiatry home visit	25.20	Based on band 5 podiatrist, 30 minute appointment, 12 minutes travelling, 5 miles travel costs (NHS travel reimbursed at 56p per mile)	
Chiropody / Podiatry phone call	5.33	Based on band 5 podiatrist, 10 minute call	
Community / District Nurse /Mental health nurse visit	22	Based on band 6 district nurse, 30 minute appointment	
Community / District Nurse /Mental health nurse home visit	46.8	Based on band 6 district nurse, 1 hour appointment including travel time, 5 miles travel costs (NHS travel reimbursed at 56p per mile)	
Community / District Nurse/ Mental health nurse phone call	7.33	Based on band 6 district nurse, 10 minute phone call	

Thorn J, *et al. BMJ Open* 2020; 10:e030110. doi: 10.1136/bmjopen-2019-030110

Service	Unit cost (£)	Notes	Source
NHS Counselling/Cognitive Behavioural Therapy visit	21	Based on band 6 counsellor, 30 minute appointment	
NHS Counselling/Cognitive Behavioural Therapy home visit	32.20	Based on band 6 counsellor, 30 minutes appointment, 12 minutes travelling, 5 miles travel costs (NHS travel reimbursed at 56p per mile)	
NHS Counselling/Cognitive Behavioural Therapy phone call	7	Based on band 6 counsellor, 10 minute call	
NHS 111 phone call	8.06	Maximum call cost of £7.80 in May 2013	[7]
NHS walk-in centre visit	41.74	Weighted mean of type 4 non-admitted, emergency medicine per attendance	[8]
OOH service visit	82.32	Average OOH hourly evening rate was £58.36 in 2005 compared with £36.75 for normal hours. Same differential applied to current GP in hours costs.	[9],[10]
OOH service home visit	158.80		
OOH service phone call	65.74		
**Ambulance services**			
Paramedic at home not involving a hospital visit	184	Ambulance services, see, treat and refer	[1]
Ambulance to hospital	238	See and treat and convey (including carbon 39 kgCO2e)	[1]
**Investigations (in outpatients)**			

5

Service	Unit cost (£)	Notes	Source
MRI	155.64		[8]
DEXA	71		
CT scan	104.88		
ECG	72		
Ultrasound	58.47		
Fluoroscopy	147.68		
**Investigations (directly accessed)**			
Cytology	16.88	Directly accessed pathology services	[8]
Histopathology and histology	30.77	Directly accessed pathology services	
Clinical Biochemistry	1.18	Directly accessed pathology services	
Haematology	3.10	Directly accessed pathology services	
Immunology	6.42	Directly accessed pathology services	
Microbiology	7.63	Directly accessed pathology services	
Other	3.13	Directly accessed pathology services	
MRI scan	147.25	Diagnostic imaging, directly accessed, weighted mean	
CT scan	107.52	Diagnostic imaging, directly accessed, weighted mean	
Ultrasound scan	52.82	Diagnostic imaging, directly accessed, weighted mean	
Bone scan	210.11	Nuclear medicine, directly accessed, weighted mean	

Thorn J, *et al. BMJ Open* 2020; 10:e030110. doi: 10.1136/bmjopen-2019-030110

Service	Unit cost (£)	Notes	Source
x-ray	30.26	Directly accessed diagnostic services, Plain film	
DEXA scan	68.29	Diagnostic imaging, direct access	
ECG	67.06	Diagnostic imaging, direct access, 19 years and over	
**Secondary care**			
Outpatient attendance	135.27	Weighted mean, consultant led	[8]
Day case	733.31	Weight mean of all daycases	
Elective inpatient	3749.81	Weighted mean	
Non-elective inpatient, long stay	3058.14	Weighted mean	
Non-elective inpatient, short stay	615.83	Weighted mean	
Elective excess bed day	361.67	Weighted mean	
Non-elective excess bed day	298.41	Weighted mean	
Individual HRGs		Mapped directly from Reference Costs file	
**Productivity losses**			
Median hourly wage, age 22-29	10.52	Excluding overtime, for employees on adult wages not affected by absence	[11]
Median hourly wage, age 30-39	13.57		
Median hourly wage, age 40-49	13.92		
Median hourly wage, age 50-59	13.18		

Service	Unit cost (£)	Notes	Source
Median hourly wage, age 60+	11.26		

* Where prescription charges are levied, patients can choose to pay for each individual item separately, or can purchase a pre-payment certificate to cover any number of prescriptions in a 3- or 12-month period ** NHS bands refer to payscales: for example, a recently qualified nurse will be paid on band 5, while a nurse with more experience might be paid on band 6.

OOH: out-of-hours. HCA: health care assistant. MRI: magnetic resonance imaging. DEXA: dual energy X-ray absorptiometry. ECG: electrocardiogram. CT: computerized tomography.

1. Curtis L, Burns A. Unit Costs of Health and Social Care 2016. University of Kent, Canterbury: Personal Social Services Research Unit 2016.
2. Curtis L. Unit Costs of Health and Social Care 2014. University of Kent, Canterbury: Personal Social Services Research Unit 2014.
3. Department of Health. NHS charges from April 2016 2016 [Available from: https://www.gov.uk/government/speeches/nhs-charges-from-april-2016.
4. Curtis L, Burns A. Unit Costs of Health and Social Care 2015. University of Kent, Canterbury: Personal Social Services Research Unit 2015.
5. Matheson C. Implementation of WebGP and e-consultations in Wessex GP practices. 2016
6. Bank of England. UK inflation calculator [Available from: http://www.bankofengland.co.uk/education/Pages/resources/inflationtools/calculator/default.aspx.
7. Health Committee. Written evidence from NHS Direct NHS Trust (ES 31) 2013 [Available from: https://www.publications.parliament.uk/pa/cm201314/cmselect/cmhealth/171/171vw25.htm.
8. Department of Health. NHS reference costs 2015 to 2016 2016 [Available from: https://www.gov.uk/government/publications/nhs-reference-costs-2015-to-2016.
9. O'Dowd A. Cost of out of hours care was 22% higher than predicted in England. *BMJ: British Medical Journal* 2006;332(7550):1113.
10. Curtis L, Netten A. Unit costs of health and social care: University of Kent; 2005 [
11. Office for National Statistics. Annual Survey of Hours and Earnings: 2016 2016 [Available from: https://www.ons.gov.uk/employmentandlabourmarket/peopleinwork/earningsandworkinghours/datasets/agegroupashetable6.
