# Supplementary material for: Cost-effectiveness of a patient-centred approach to managing multimorbidity in primary care: a pragmatic cluster randomised controlled trial
Source: BMJ Open. 2020 Jan 19;10(1):e030110. doi: 10.1136/bmjopen-2019-030110 (PMC7044971; doi:10.1136/bmjopen-2019-030110)
Supplement: Supplementary data [file bmjopen-2019-030110supp002.pdf]

Appendix 2. **Glossary of economic terms.**

Economic evaluation

Economic evaluation refers to an analysis comparing both costs and outcomes (or benefits) to assess the value for money that an intervention offers.

Cost-consequences analysis

A cost-consequences analysis (CCA) is a type of economic evaluation in which the costs and the outcomes are presented in disaggregated form. No attempt is made to combine costs and outcomes into a cost-effectiveness statistic, instead providing commissioners with a broad range of relevant information, and allowing the scope to apply their own priorities.

Cost-effectiveness analysis

A cost-effectiveness analysis (CEA) is a type of economic evaluation in which the outcomes (or benefits) are measured in terms of a single (usually) clinical measure. For example, a cost-effectiveness analysis might have a result expressed in terms of cost per life-year gained.

Cost-utility analysis

A cost-utility analysis (CUA) is a type of economic evaluation in which the outcome is assessed in terms of a measure of health outcome to which preference-based valuations have been attached. The most commonly used outcome is the quality-adjusted life year (QALY). CUA is a particular form of CEA.

Net monetary benefit

The net monetary benefit is a cost-effectiveness statistic combining costs and outcomes in a single measure, and taking into account the societal ‘willingness-to-pay’ for a unit of improvement. A positive incremental net monetary benefit indicates that society is willing to pay for the benefits that can be gained.
