# Supplementary material for: Cost-effectiveness of a patient-centred approach to managing multimorbidity in primary care: a pragmatic cluster randomised controlled trial
Source: BMJ Open. 2020 Jan 19;10(1):e030110. doi: 10.1136/bmjopen-2019-030110 (PMC7044971; doi:10.1136/bmjopen-2019-030110)
Supplement: Supplementary data [file bmjopen-2019-030110supp003.pdf]

Appendix 3. Mean number of health and social care resource-use contacts over 15 months of follow-up.

**Services**	**Usual care**		**Intervention**	
	**mean (SD)**	***N***	**mean (SD)**	***N***
Inpatient stays	0.50 (1.05)	722	0.55 (1.09)	766
Inpatient nights	4.51 (16.6)	722	3.84 (11.94)	766
Outpatient visits	3.60 (4.00)	722	3.64 (3.73)	766
Day cases	0.15 (0.54)	722	0.14 (0.51)	766
Accident and emergency visits	0.63 (1.41)	722	0.61 (1.14)	766
Ambulance journeys	0.55 (1.83)	601	0.60 (3.50)	615
GP face-to-face consultations	7.27 (5.93)	715	8.70 (6.26)	754
GP home visits	0.64 (1.89)	715	0.79 (2.16)	754
GP phone calls	2.88 (5.00)	715	2.99 (4.53)	754
Nurse face-to-face consultations	5.40 (5.70)	715	7.93 (10.09)	754
Nurse home visits	0.06 (0.40)	715	0.09 (0.54)	754
Nurse phone calls	0.60 (1.56)	715	0.76 (1.63)	754
Healthcare assistant face-to-face consultations	2.42 (4.30)	715	3.55 (7.75)	754
Healthcare assistant GP home visits	0.00 (0.05)	715	0.08 (0.28)	754
Healthcare assistant GP phone calls	0.03 (0.17)	715	0.03 (0.17)	754
Prescribed medications	143.44 (130.57)	717	132.23 (117.36)	755
GP investigations	19.60 (16.52)	717	22.61 (16.99)	755
Pharmacist reviews		722	0.78 (0.42)	766
Occupational therapist clinic visits	0.18 (1.10)	601	0.2 (1.28)	615
Occupational therapist home visits	0.24 (1.29)	601	0.08 (0.5)	615
Occupational therapist phone calls	0.06 (0.53)	601	0.01 (0.14)	615
Speech and language therapist clinic visits	0.02 (0.33)	601	0.1 (1.03)	615
Speech and language therapist home visits	0.04 (0.69)	601	0 (0)	615
Speech and language therapist phone calls	0.00 (0.08)	601	0 (0)	615
Physiotherapist clinic visits	0.80 (2.74)	601	1.03 (2.67)	615
Physiotherapist home visits	0.16 (1.34)	601	0.15 (0.92)	615
Physiotherapist phone calls	0.00 (0.06)	601	0.01 (0.11)	615
Podiatrist clinic visits	1.26 (2.83)	601	1.19 (2.53)	615
Podiatrist home visits	0.13 (0.84)	601	0.21 (1.46)	615
Podiatrist phone calls	0.01 (0.13)	601	0.02 (0.49)	615
Community mental health nurse clinic contacts	0.27 (2.59)	601	0.08 (0.42)	615
Community mental health nurse home visits	0.21 (2.45)	601	0.35 (5.16)	615
Community mental health nurse phone calls	0.02 (0.23)	601	0.01 (0.13)	615

District nurse home visits	1.21 (4.08)	601	1.48 (6.54)	615
District nurse phone calls	0.03 (0.30)	601	0.05 (0.84)	615
Counsellor clinic visits	0.24 (2.34)	601	0.14 (0.99)	615
Counsellor home visits	0.01 (0.10)	601	0.01 (0.18)	615
Counsellor phone calls	0.00 (0.08)	601	0.00 (0.06)	615
NHS 111 phone calls	0.21 (0.85)	601	0.18 (0.57)	615
NHS walk-in centre visits	0.08 (0.41)	601	0.09 (0.50)	615
GP out-of-hours clinic visits	0.08 (0.34)	601	0.07 (0.28)	615
GP out-of-hours home visits	0.04 (0.33)	601	0.06 (0.56)	615
GP out-of-hours phone calls	0.02 (0.20)	601	0.03 (0.28)	615
Paramedic attendances at home	0.09 (0.43)	601	0.05 (0.29)	615
Other healthcare clinic visits	0.11 (0.88)	601	0.25 (3.24)	615
Other healthcare home visits	0.06 (0.65)	601	0.04 (0.41)	615
Other healthcare phone calls	0.01 (0.19)	601	0.01 (0.10)	615
Carer contacts	30.93 (148.26)	601	25.27 (135.78)	615
Daycare contacts	2.14 (17.53)	601	1.17 (10.50)	615
Meals on wheels	2.66 (28.32)	601	0.93 (18.98)	615
Social worker contacts	0.77 (11.35)	601	0.45 (3.98)	615
