# Supplementary material for: Cost-effectiveness of a patient-centred approach to managing multimorbidity in primary care: a pragmatic cluster randomised controlled trial
Source: BMJ Open. 2020 Jan 19;10(1):e030110. doi: 10.1136/bmjopen-2019-030110 (PMC7044971; doi:10.1136/bmjopen-2019-030110)
Supplement: Author's manuscript [file bmjopen-2019-030110.draft_revisions.pdf]

BMJ Open

BMJ Open is committed to open peer review. As part of this commitment we make the peer review history of every article we publish publicly available.

When an article is published we post the peer reviewers' comments and the authors' responses online. We also post the versions of the paper that were used during peer review. These are the versions that the peer review comments apply to.

The versions of the paper that follow are the versions that were submitted during the peer review process. They are not the versions of record or the final published versions. They should not be cited or distributed as the published version of this manuscript.

BMJ Open is an open access journal and the full, final, typeset and author-corrected version of record of the manuscript is available on our site with no access controls, subscription charges or pay-per-view fees (http://bmjopen.bmj.com).

If you have any questions on BMJ Open's open peer review process please email editorial.bmjopen@bmj.com

Cost-effectiveness of a patient-centred approach to managing multimorbidity in primary care: a pragmatic cluster randomised controlled trial

Journal:	*BMJ Open*
Manuscript ID	bmjopen-2019-030110
Article Type:	Research
Date Submitted by the Author:	27-Feb-2019

Complete List of Authors:	Thorn, Joanna; University of Bristol, Population Health Sciences<br>Man, Mei-See; University of Bristol School of Social and Community Medicine,<br>Chaplin, Katherine ; University of Bristol Centre for Academic Primary Care, Bristol Medical School<br>Bower, Peter; University of Manchester, NPCRDC<br>Brookes, Sara; Institute of Cancer and Genomic Sciences, University of Birmingham, Cancer Research UK Clinical Trials Unit ; University of Bristol, Population Health Sciences<br>Gaunt, Daisy; University of Bristol Faculty of Medicine and Dentistry, Bristol Randomised Trials Collaboration & School of Social and Community Medicine; University of Bristol<br>Fitzpatrick, Bridie; University of Glasgow, Institute of Health and Wellbeing<br>Gardner, Caroline; University of Manchester Faculty of Biology, Medicine and Health<br>Guthrie, Bruce; University of Dundee, Population Health Sciences Division, Medical Research Institute<br>Hollinghurst, Sandra; University of Bristol, Community Based Medicine<br>Lee, Victoria; University of Manchester Faculty of Biology, Medicine and Health<br>Mercer, Stewart; University of Glasgow, Institute of Health and Wellbeing<br>Salisbury, Chris; University of Bristol, Academic Unit of Primary Health Care

Keywords:	Multimorbidity, PRIMARY CARE, patient-centred care, economic evaluation

Note: The following files were submitted by the author for peer review, but cannot be converted to PDF. You must view these files (e.g. movies) online.
figure1.tif

Cost-effectiveness of a patient-centred approach to managing multimorbidity in primary care: a pragmatic cluster randomised controlled trial

Joanna C Thorn[1], Mei-See Man[1,2], Katherine Chaplin[1], Peter Bower[3], Sara T Brookes[2], Daisy Gaunt[2], Bridie Fitzpatrick[4], Caroline Gardner[3], Bruce Guthrie[5], Sandra Hollinghurst[1], Victoria Lee[3], Stewart W Mercer[6], Chris Salisbury[1]

[1] Centre for Academic Primary Care, Population Health Sciences, Bristol Medical School, University of Bristol, Canynge Hall, 39 Whatley Road, Bristol, BS8 2PS, UK
[2] Bristol Randomised Trials Collaboration, Population Health Sciences, University of Bristol, Canynge Hall, 39 Whatley Road, Bristol, BS8 2PS, UK
[3] NIHR School for Primary Care Research, Centre for Primary Care and Health Services Research, Faculty of Biology, Medicine and Health, University of Manchester, Manchester Academic Health Science Centre, UK
[4] Institute of Health and Wellbeing, University of Glasgow, 1 Lilybank Gardens, Glasgow, G12 8RZ, UK
[5] Division of Population Health Sciences, Medical Research Institute, University of Dundee, Dundee, UK
[6] Usher Institute, University of Edinburgh, Old Medical School, Teviot Place, Edinburgh, EH8 9AG, UK

Corresponding author: Dr Joanna Thorn, Population Health Sciences, University of Bristol, 1-5 Whiteladies Road, Bristol, BS8 1NU, UK; joanna.thorn@bristol.ac.uk; Tel. +44 (0) 117 4283125

3999 words

Funding: This project was funded by the National Institute for Health Research (NIHR) Health Services and Delivery Research Programme (project number 12/130/15). This study was designed and conducted in collaboration with the Bristol Randomised Trials Collaboration (BRTC), a UKCRC Registered clinical trials unit (CTU) in receipt of NIHR CTU support funding. CS is partly supported by The NIHR Collaboration for Leadership in Applied Health Research and Care West (CLAHRC West) and by Bristol Clinical Commissioning Group.

The views and opinions expressed in this report are those of the authors and do not necessarily reflect those of the NIHR, the NHS or the Department of Health.

Keywords: Multimorbidity, primary care, economic evaluation, patient-centred care

Running title: Multimorbidity management in primary care

For peer review only - http://bmjopen.bmj.com/site/about/guidelines.xhtml

Acknowledgments: We would like to thank the patients and practices who participated in the study, and the other members of the 3D study team (Cindy Mann, Polly Duncan, Keith Moffatt, Emma Moody, Imran Rafi, Becca Robinson, Ali Heawood, Bryar Kadir, John McLeod). We would also like to thank Professor Joanna Coast and Dr Padraig Dixon for valuable guidance. This article is an abbreviated version of the final report to the funders (Salisbury C, Man M-S, Chaplin K, Mann C, Bower P, Brookes S, et al. A patient-centred intervention to improve the management of multimorbidity in general practice: the 3D RCT. *Health Serv Deliv Res* 2019;7(5)).

Author contributions

CS, PB, BG, STB, SH and SWM conceived, designed and interpreted results from the 3D trial. SH and JCT designed the economic evaluation. M-SM (trial manager), KC, BF, CG and VL contributed to local economic data collection and interpretation of results. DG (statistician) conducted the multiple imputation. JCT carried out the analysis and wrote the paper, and all authors revised it for important intellectual content.

Competing interests statement

CS reports grants from CLAHRC West and Bristol CCG outside the submitted work. No other competing interests are declared.

For peer review only

Abstract

Objective Patients with multiple chronic health conditions are often managed in a disjointed fashion in primary care, with annual review clinic appointments offered separately for each condition. This study aimed to determine the cost-effectiveness of the 3D intervention, which was developed to improve the system of care.

Design Economic evaluation conducted alongside a pragmatic cluster-randomised trial.

Setting General practices in three centres in England and Scotland.

Participants 797 adults with three or more chronic conditions were randomised to the 3D intervention, while 749 participants were randomised to receive usual care.

Intervention The 3D approach: comprehensive 6-monthly GP consultations, supported by medication reviews and nurse appointments.

Primary and secondary outcome measures The primary economic evaluation assessed the cost per quality-adjusted life year (QALY) gained from the perspective of the NHS and personal social services (PSS). Costs were related to changes in a range of secondary outcomes (QALYs accrued by both participants and carers, and deaths) in a cost–consequences analysis from the perspectives of the NHS/PSS, patients/carers and societal productivity.

Results

Very small increases were found in both QALYs (adjusted mean difference 0.007 [-0.009 to 0.023]) and costs (adjusted mean difference £126 [£-739 to £991]) in the intervention arm compared with usual care after 15 months. The incremental cost-effectiveness ratio was £18,499, with a 50.8% chance of being cost-effective at a willingness-to-pay threshold of £20,000 per QALY (55.8% at £30,000 per QALY).

Conclusions

The small differences in costs and outcomes were consistent with chance, and the uncertainty was substantial; therefore, the evidence for the cost-effectiveness of the 3D approach from the NHS/PSS perspective should be considered equivocal. These results should be considered in conjunction with evidence from the participants themselves about their experience of patient-centred care and with other outcomes to inform a decision on implementation.

For peer review only - http://bmjopen.bmj.com/site/about/guidelines.xhtml

Article summary

Strengths and limitations of this study

- This economic evaluation was conducted alongside the largest randomised controlled trial of an intervention for managing multimorbid patients in primary care.
- Data collection was meticulous and high questionnaire return rates were achieved.
- Data on the use of care homes, which may contribute significantly to social care costs, were not available to include in the economic evaluation.
- Estimates of healthcare costs for this type of intervention have wide confidence intervals, even with a substantial sample size.

For peer review only

Data sharing statement

Requests for data sharing should be submitted to the Professor C Salisbury (c.salisbury@bristol.ac.uk) for consideration. Access to anonymised data might be granted following review via the University of Bristol Research Data Storage Facility.

For peer review only - http://bmjopen.bmj.com/site/about/guidelines.xhtml

Introduction

The number of patients living with multiple chronic health conditions (multimorbidity) is increasing in developed countries as the population ages[1]. Multimorbidity is associated with poor health-related quality of life[2 3], higher use of health services and higher costs[4 5]. Multimorbidity is the norm for older adults (with a prevalence of up to 98%[4]), leading to a substantial economic burden[6].

Care pathways for patients with multimorbidity are often poorly co-ordinated and burdensome for patients. Each condition may be treated separately, which fails to take account of interactions between diseases[1]. There have been relatively few randomised controlled trials (RCTs) of interventions designed to address issues affecting multimorbid patients, with mixed findings leading to substantial uncertainty about the effectiveness of such interventions[7]. Evidence on the cost-effectiveness of interventions remains even more limited and the evidence that is available is inconsistent[7-9]. A recent review of comprehensive care programmes for multimorbidity found no evidence that such interventions reduce healthcare costs or primary care visits[10]. Several reviews have highlighted the need for research on new interventions, including assessment of cost-effectiveness[1 7 11].

The '3D' intervention was developed to address the issues associated with managing patients with multimorbidity in primary care in the UK. The intervention was based on a patient-centred care model and aimed to improve continuity of care with caregivers in GP surgeries, reduce patient burden in accessing healthcare and increase patient involvement in decision-making about their care. The 3D randomised trial assessed the effectiveness of the 3D approach compared with usual care, and incorporated an embedded process evaluation. The trial did not find any differences in health-related quality of life over 15 months of follow-up, but the intervention did lead to improved patient-centred care[12 13]. Here, we report the results of the economic evaluation conducted alongside the 3D trial.

Methods

The 3D trial

Full details of the study are given elsewhere[13 14]. A favourable ethical opinion was granted by the South-West (Frenchay) National Health Service (NHS) Research Ethics Committee (14/SW/0011), and the trial was prospectively registered (ISCRTN06180958). A patient and public advisory group was recruited before the trial began, which contributed to discussions about many aspects of trial design, interpretation and dissemination. In brief, the 3D cluster randomised trial compared the effectiveness and cost-effectiveness of a complex intervention for multimorbidity with usual care delivered in 33 general practices in Scotland and England. Multimorbidity was defined as having three or more chronic conditions from those included in the NHS Quality and Outcomes Framework[15]. Clinical staff in intervention practices

For peer review only - http://bmjopen.bmj.com/site/about/guidelines.xhtml

attended two half-day 3D training sessions. Participants in the intervention arm were offered six-monthly holistic reviews of their health problems. Each review consisted of a nurse review appointment to collect relevant clinical information, a medication review conducted by a pharmacist and an extended appointment for the patient to discuss their conditions with a named GP. An individual health plan was negotiated with the patient, detailing their own priorities for managing their conditions. The primary outcome measure for effectiveness was health-related quality of life at 15 months after randomisation, measured using the EQ-5D-5L[16].

The primary economic cost–utility analysis was conducted from the NHS and personal social services (NHS/PSS) perspective over 15 months of follow-up. A secondary analysis was conducted from the perspective of patients and carers.

Economic outcomes

The primary economic outcome measure was quality-adjusted life years (QALYs). EQ-5D-5L measurements were collected by postal questionnaires at baseline, 9 months and 15 months post-recruitment, supplemented by telephone collection for non-responders. Utility scores based on a UK population were derived from responses to the EQ-5D-5L cross-mapped to valuations obtained for the EQ-5D-3L instrument[17]. This was a change (approved by the Independent Data Monitoring Committee) to the analysis originally planned, as the National Institute for Health and Care Excellence (NICE) issued a position statement recommending this approach[18] subsequent to the design of the study but prior to analysis. QALYs over the 15-month period were formed from these valuations by means of linear interpolation and an area under the curve calculation. Patients who died during follow-up were treated as if their most recent utility score was relevant until death, and set to zero immediately at death.

Resource-use measurement

As the trial population had multimorbidity, resource use related to any health condition experienced by the participant was considered relevant.

Staff training attendance records were kept to track resources used to deliver the training programmes for GPs, nurses and receptionists, including trainee and trainer delivery time (and preparation time), travel costs, and course materials. 3D GP appointments, nurse appointments and pharmacist reviews were captured by downloading routine GP records, supplemented by manual data capture by researchers reviewing participants' medical records at the end of the trial.

Data on medications prescribed and tests/investigations in primary care were extracted from downloads of routine GP records. Details of the number and duration of primary care consultations (other than 3D reviews) were similarly extracted. These included face-to-face, telephone and home consultations with

For peer review only - http://bmjopen.bmj.com/site/about/guidelines.xhtml

doctors, nurses or health care assistants based in general practice. Duration details were not available for practices in Scotland. Therefore, an average duration for each type of consultation by each staff type in each arm was derived using available data (practices in England only) and applied to all relevant consultations. NHS community care, care from social services and patient personal resource use during the 15-month follow-up period after recruitment were captured in the patient-reported questionnaires. The research team collected NHS secondary care data from participants' GP records.

To assess travel costs, the patient's normal transport method for GP appointments and the cost (for public transport) or mileage (for private transport) were collected in the patient-reported questionnaire at baseline. Time off work for patients and carers to attend hospital appointments was similarly collected from patients at 9 and 15 months. Usual practice with regards to time off work for GP appointments was recorded at baseline. Over-the-counter medication expenditure was captured in the patient-reported questionnaires at 9 and 15 months, as was private use of treatments and therapies. Details of whether the participant paid prescription charges or not were requested in the questionnaire at baseline.

Valuation of resource use

Unit costs for NHS staff time for training and delivery of the intervention were based on the most recently available national estimates[19]. Actual expenses relating to training materials, refreshments and staff travel were recorded. Because only some GPs and a small proportion of patients in each practice participated in the trial, costs were adjusted to estimate the cost of training a full practice, divided by the number of eligible patients in the practice and annualised over an estimated 5-year period of relevance. Medication costs were downloaded directly as part of the routine GP records, supplemented by estimates from the British National Formulary where necessary[20]. Patient prescription charges were subtracted from the NHS perspective medication costs. Community and primary care costs were based on recent national estimates[19].

Secondary care contacts were assigned to a healthcare resource group (HRGs, groups of events that have been judged to consume similar levels of resources) and costed based on national reference costs[21]. Productivity costs were estimated based on average weekly earnings stratified by age group[22]. Mileage costs were estimated using UK government allowances[23]. Costs for over-the-counter medication and private therapies and treatments were all reported directly by patients. Unit costs used in the analyses are detailed in appendix 1.

All costs were reported in 2015/2016 pounds sterling, adjusted for inflation where necessary. Costs and outcomes occurring during the final three months of follow-up were discounted in line with NICE guidance (currently 3.5%)[24]. Dates were not available for all types of resource use measured in the trial; in these cases, 50% of the costs incurred in the final six months of follow-up were subjected to discounting.

For peer review only - http://bmjopen.bmj.com/site/about/guidelines.xhtml

Data cleaning and missing costs and outcomes

Data cleaning was undertaken prior to unblinding. Questionnaires were not classed as 'missing data' for the cost analysis unless the questionnaire was not returned or the majority of responses were uninterpretable. Medication costs downloaded from practices were manually amended where they were clearly wrong (*e.g.* a prescription for a salbutamol inhaler with a recorded cost of over £1000). The primary analysis included all participants using imputation to predict missing costs and outcomes[25]. Data imputed (using chained equation multiple imputation methods) for the main statistical analysis were used for the economic evaluation[12 26 27]. To facilitate convergence of the imputation model, costs were imputed using aggregated cost categories (medications, pharmacy reviews, secondary care, primary care, social care and other types of care) rather than at the level of individual resource-use items.

Economic analyses

Statistical analyses were conducted using Stata 14.2[28]. All analyses were by 'intention to treat' (comparing the two groups as randomised and including all patients in the primary analysis).

A cost–utility analysis was conducted from the NHS/PSS perspective corresponding to the NICE reference case[24]. The incremental mean difference in QALYs between the two arms of the trial and 95% confidence intervals were derived. Overall mean NHS/PSS costs, and standard errors for both arms of the trial were calculated. The incremental mean difference in total costs between the two arms of the trial and 95% confidence intervals were estimated.

Cost and QALY data were combined to calculate an incremental cost-effectiveness ratio (ICER) and net monetary benefit (NMB) statistic[29] from the NHS/PSS perspective. In the primary analysis we estimated whether the 3D approach was cost-effective at the established NICE thresholds of £20,000 and £30,000 per QALY gained. The probability that the 3D approach was cost-effective at various societal 'willingness to pay for a QALY' thresholds was depicted using a cost-effectiveness acceptability curve (CEAC). All measures of cost-effectiveness (ICER, CEAC and NMB) and confidence intervals were derived parametrically using the output of seemingly-unrelated regression analysis to account for the correlation between costs and outcomes, and controlling for baseline imbalance in utility for the QALY equation[30]. Clustering within GP practices was accounted for by including the randomisation variables in the regression.

The costs of each component of the intervention were estimated separately from the NHS/PSS perspective, the patient/carer perspective and the societal productivity perspective and related to changes in a range of secondary outcomes in a cost–consequences analysis[31]. Consequences included QALYs accrued by both participants and carers, and deaths. The cost-consequences analysis was based on

For peer review only - http://bmjopen.bmj.com/site/about/guidelines.xhtml

available cases, which differed in number for each type of healthcare resource or outcome; an available case was defined as an individual having complete data for each relevant timepoint. Linear regression output was used to derive confidence intervals parametrically, accounting for clustering within practices.

Sensitivity analyses

One-way sensitivity analyses were used to judge the potential impact of sources of uncertainty. This included an analysis excluding participants who died to assess the impact of the imbalance in deaths between arms, and an analysis without discounting either costs or outcomes to assess the impact of the discount rate. A complete-case analysis was also conducted to assess the impact of the imputation process; a complete case was defined as a participant for whom full resource-use data and full outcome data were available.

Results

The results are reported according to the specifications of the Consolidated Health Economic Evaluation Reporting Standards (CHEERS) statement[32].

Missing data

16 practices (797 participants) were randomised to be offered the 3D approach, while 17 practices (749 participants) were randomised to receive usual care. Missing data occurred for a number of reasons, including withdrawal from the trial, or leaving the practice. 12 participants (1.5%) in the 3D arm and six (0.8%) in the control arm had no information on secondary care use because it was not possible to locate their medical records (*p*=0.2). Practice downloads of medication and investigation data failed for 18 participants (2.3%) in the intervention arm and eight (1.1%) in the usual care arm (*p*=0.07), while 19 (2.4%) and 10 (1.3%) 3D and usual care participants respectively were missing consultation data from practice downloads (*p*=0.13). Not all participants returned all questionnaires at all timepoints. 165 participants (20.7%) in the intervention arm and 125 (16.7%) in the control arm did not return a questionnaire at one or more follow-up points (*p*=0.04). Not all those who did return questionnaires completed the resource-use questions; in total, 181 (22.7%) in the intervention arm and 146 (19.5%) in the control arm were missing resource-use data from questionnaires at one or more follow-up points (*p*=0.12). Complete datasets were available for 1191 participants in total (599 (75.2%) in the 3D arm and 592 (79%) in the control arm, *p*=0.07). Participants with missing data were in a significantly poorer health state at baseline (mean (95%CI) EQ-5D-5L score: 0.453 (0.422 to 0.485)) than participants with full datasets (mean (95%CI) EQ-5D-5L score: 0.589 (0.574 to 0.605)).

For peer review only - http://bmjopen.bmj.com/site/about/guidelines.xhtml

Primary analysis

Outcomes and resource use

The primary analysis using imputed data showed that participants in the intervention arm gained a mean of 0.007 additional QALYs over 15 months compared with participants in the usual care arm (95%CI: -0.009 to 0.023). Total costs per patient from the NHS/PSS perspective were £126 (95%CI: -£739 to £991) higher in the intervention arm than in the usual care arm. Wide variability was observed in both costs and outcomes, with both confidence intervals overlapping zero. Disaggregated resource-use data are in appendix 2.

Cost-effectiveness of 3D

Cost-effectiveness statistics from the NHS/PSS perspective are given in Table 1. The ICER was £18,499, and the net monetary benefit at a societal willingness-to-pay value of £20,000 was £10 (95%CI: -£956 to £977). At this willingness-to-pay value, the probability that the 3D approach is cost-effective was 0.508, while at £30,000, the probability of cost-effectiveness was 0.558. A cost-effectiveness acceptability curve depicting the probability of cost-effectiveness at a range of willingness-to-pay values is given in Figure 1.

Sensitivity analyses

Results from an analysis restricted to complete cases only are given in Table 2. The complete-case analysis suggested that the 3D approach was associated with both lower costs and better outcomes, with a probability of cost-effectiveness of 0.705 at a willingness-to-pay threshold of £20,000. A sensitivity analysis excluding participants who died suggested that the probability of cost-effectiveness of the 3D approach at £20,000 was 0.561. A further sensitivity analysis using undiscounted costs and outcomes did not suggest that the discount rate affected the conclusions.

Cost–consequences analysis

Costs and selected outcomes (on an available case basis) are presented in Table 3 from the primary perspective of the NHS/PSS and the secondary perspective of the patient/carer themselves alongside an estimate of the societal loss of productivity.

Costs from all perspectives were very similar between arms and no cost group differed significantly (other than those associated with the intervention itself). Other than for daycase/outpatient care, emergency care and medications, costs to the NHS were higher in the intervention arm than in the usual care arm, while social services usage was higher in the usual care arm. Overall costs (based on available data) from the NHS/PSS perspective were slightly higher in the usual care arm although the difference was consistent with a chance finding.

For peer review only - http://bmjopen.bmj.com/site/about/guidelines.xhtml

Costs borne by patients and carers were higher overall in the intervention arm, although the medication costs (both prescription charges and over-the-counter remedies) were slightly lower; no patient cost group exhibited a statistically significant difference. The societal cost of productivity losses was similar in the two arms (and statistically consistent with chance), although slightly higher in the 3D approach arm.

QALYs (adjusted for baseline utility scores) were slightly higher for patients and lower for carers in the intervention arm than in the usual care arm; however, the difference was consistent with chance. Although there was a higher number of deaths in the intervention arm, the difference was not statistically significant.

Discussion

No difference was observed between participants offered the 3D intervention and those receiving usual care only for overall costs, resource use of any category, or QALY outcomes. A small improvement in outcome (representing an additional 2.6 days of the best imaginable health over 15 months) coupled with a small increase in costs between arms led to weak evidence for the cost-effectiveness of the 3D approach in the primary analysis from the NHS/PSS perspective. The net monetary benefit was small, but positive, indicating that the costs associated with the intervention are less than society is willing to pay for the benefits that can be achieved. Anticipated reductions in appointment attendances were not achieved, partly because some patients attended 3D reviews in addition to, rather than instead of, their single-condition reviews as intended. Similarly, although cost savings could have been achieved through a reduction in prescriptions issued, this was not observed in the trial. This could be because although the brief for the pharmacists was to seek to simplify patients' medication regimes, the embedded process evaluation[13] suggested that pharmacists appeared to concentrate on adherence to guidelines rather than whether the patient might benefit from stopping some medications.

From the NHS/PSS perspective, costs were slightly higher in the intervention arm when based on the full imputed dataset, but were slightly lower in the intervention arm when only complete cases were examined. This suggests that the participants with missing data were higher users of health and social care than responding participants, which is consistent with the complete cases having higher utility at baseline than those with missing data. Although the complete-case analysis suggested that the 3D approach was dominant (*i.e.* it provided higher gains at lower cost than usual care), the results should be treated with caution given the substantial uncertainty, and the biased sample of healthier participants.

The 3D participants had a mean(sd) utility of 0.558(0.287) at entry to the study, which compares poorly to a UK population mean of 0.779 for ages 65-74[33]. The participants were substantial users of healthcare, with inpatient hospital care and medications both high contributors to overall costs. Participants had a

For peer review only - http://bmjopen.bmj.com/site/about/guidelines.xhtml

small positive increase in QALYs in the intervention arm, while carers had a small decrease in QALYs compared with the usual care arm; it is possible that an analysis taking carer outcomes into account might reach an alternative conclusion. The small contribution to overall costs made by productivity losses is consistent with the predominantly retired study population; at baseline, over 65% of participants described themselves as 'fully retired from work'.

Comparison with other studies is hampered by the variety of definitions used for multimorbidity, and by the heterogeneity in study designs[11]. Evidence concerning the cost-effectiveness of interventions designed to manage patients with multimorbidity is limited; only two of the RCTs identified in the review by Smith *et al* undertook cost-effectiveness analyses[7]. Katon *et al* reported that implementation of a collaborative care intervention for patients with depression and either diabetes or coronary heart disease was justified on cost-effectiveness grounds[34]. However, the authors estimated QALYs based on clinical outcomes rather than by direct measurement, and there was substantial uncertainty around both costs and outcomes. Barley *et al* found that a nurse-led patient-centred intervention was cost-effective up to a willingness-to-pay threshold of approximately £3000 per QALY; however, the study was a pilot RCT, and multimorbidity was defined to include patients with coronary heart disease and depression only[35]. In a feasibility trial, Mercer *et al* found that a whole-system intervention designed for patients with two or more chronic conditions in deprived areas may be considered cost-effective[36]. There is a clear need for further well conducted studies on the cost-effectiveness of interventions for multimorbid patients.

A crude estimate of the budget impact of implementing the intervention in England can be made using the trial results. England had a population of 43.5 million adults in 2016[37]. 3.7% of adults in the trial practices were both eligible for the 3D approach, and considered suitable by their GP, suggesting an eligible population of 1.6 million people. At an incremental cost of £126 over 15 months, the intervention may cost approximately £162 million per year, although there is considerable uncertainty around this sum, and it is probable that the costs of delivering the intervention could be reduced if the intervention became the norm. The estimate represents a comparable investment to the budget of £162 million allocated to a recent UK initiative aiming to avoid unplanned admissions for vulnerable people[38].

Strengths and limitations

This economic evaluation was conducted alongside a large and rigorous RCT. Meticulous data collection practices allowed individual patient data to be measured for all the key cost drivers, a particularly challenging task for a patient group that has high healthcare usage. The study contributes to the growing body of evidence supporting the care of patients with multimorbidity. Patterns of missing resource-use data were similar between arms, and high questionnaire return rates were achieved[39]. Although complete-case analyses suggested a different conclusion from the primary imputed analysis, the results were consistent with the small differences in costs and outcomes observed between arms. Even though

For peer review only - http://bmjopen.bmj.com/site/about/guidelines.xhtml

this is the largest trial of an intervention for multimorbidity ever undertaken, there remains substantial uncertainty surrounding the results. The impact of organisational changes in delivery of primary health care on patients' quality of life is usually very small and the costs are also usually low, but skewed by a small number of patients with high costs in secondary care often unrelated to the intervention. This leads to wide confidence intervals in estimates of cost-effectiveness and illustrates the difficulty of obtaining precise estimates for the economic outcomes of this type of intervention even in large trials.

Medication costs were based on prescriptions issued by the GP, and it is not certain that all scripts were filled by the participant; the medication costs may, therefore, be overestimated. In addition, a number of errors were identified in the medication costs downloaded from GP practice notes; although both high and low outliers were checked carefully and manually corrected where necessary, the volume of medications prescribed to the 3D population rendered it infeasible to check every entry, and it is possible that some errors persisted.

Use of care homes was not included in the economic evaluation. This can be a significant contributor to costs of social care but the funding of care homes within the UK is complex, with patients often paying considerable amounts themselves. However, only 13 participants reported living in a care home and this did not differ by arm. The follow-up period of the 3D trial was 15 months, and longer-term outcomes are unknown; a follow-up period of 2–3 years might have allowed an effect on quality of life or healthcare resource use to be observed. Use of simple mean imputation methods for estimating missing information (such as the cost of a bus fare) in the questionnaire data will have reduced standard errors and underestimated the uncertainty around these costs. The software template used to manage the 3D approach was developed using trial funding, and would not incur significant ongoing costs; however, development costs would be incurred for the intervention to be implemented in other software systems.

QALY outcomes for participants who died were based on an immediate reduction from the previous known EQ-5D-5L value to zero at death. Although this will be accurate for some participants who died suddenly after living previously in a consistent health state, it is likely that some patients would have undergone a decline in health-related quality of life while approaching death. The QALY gains may, therefore, be slightly overstated.

It is possible that the cost-effectiveness of the intervention could be increased by focusing more on patients with the greatest needs, by more attention to de-prescribing, and by ending unnecessary additional disease-specific reviews as the 3D model became normalised.

For peer review only - http://bmjopen.bmj.com/site/about/guidelines.xhtml

Conclusions

The evidence for the cost-effectiveness of the 3D intervention is equivocal; the results suggest that there is just over a 50% chance of cost-effectiveness at the established threshold of £20,000 per QALY from the NHS/PSS perspective. The small differences in costs and outcomes are consistent with chance, and the uncertainty is substantial; therefore, the results should be interpreted with caution. Given the equivocal nature of the cost-effectiveness results, implementation of the intervention cannot be recommended on economic grounds alone; however, given the modest implementation costs, the intervention might be justified on the basis of the improvements observed in participants' experience of patient-centred care[12].

For peer review only

For peer review only - http://bmjopen.bmj.com/site/about/guidelines.xhtml

Figure 1. Cost-effectiveness acceptability curve from the NHS/PSS perspective.†

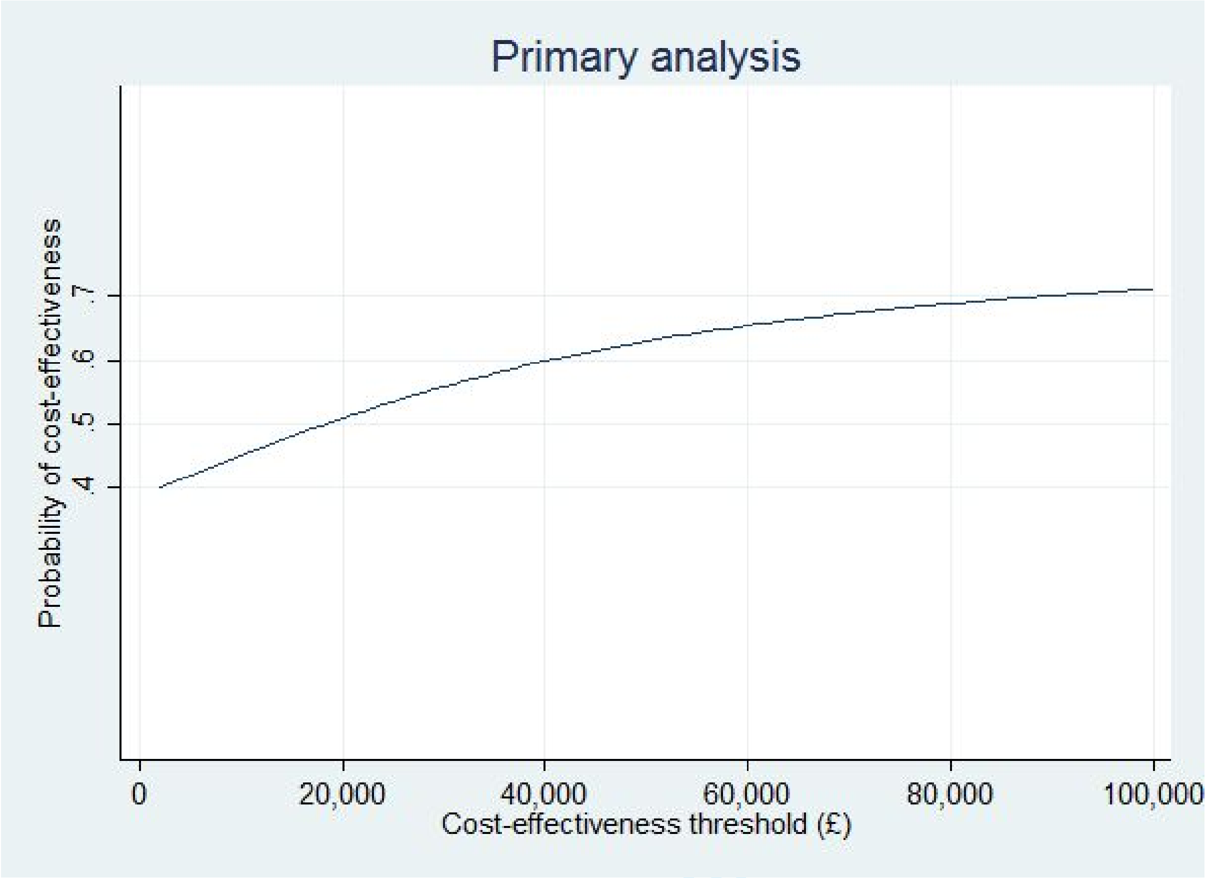

† Salisbury C, Man M-S, Chaplin K, Mann C, Bower P, Brookes S, et al. A patient-centred intervention to improve the management of multimorbidity in general practice: the 3D RCT. *Health Serv Deliv Res* 2019;7(5)

For peer review only - http://bmjopen.bmj.com/site/about/guidelines.xhtml

Table 1. Cost-effectiveness of the 3D approach from an NHS and personal social services perspective

	**Usual care Mean (SE) N=749**	**Intervention Mean (SE) N=797**	**Incremental difference (95% CI)**
**Costs (£)**			
Unadjusted costs from the NHS/PSS perspective	6032 (362)	6124 (317)	
Adjusted costs from the NHS/PSS perspective	6014 (343)	6140 (333)	126 (-739 to 991)
**Outcomes**			
Unadjusted QALYs over 15 months of follow-up	0.651 (0.013)	0.691 (0.012)	
Adjusted QALYs over 15 months of follow-up	0.668 (0.006)	0.675 (0.006)	0.007 (-0.009 to 0.023)
**Cost-effectiveness statistics**			
ICER: £18499			
Net monetary benefit at £20,000 (95%CI): £10 (-956 to 977)			
Net monetary benefit at £30,000 (95%CI): £78 (-974 to 1130)			

ICER, incremental cost-effectiveness ratio; CI, confidence interval; PSS, personal social services; QALY, quality-adjusted life year; SE, standard error. QALY estimates were adjusted for baseline utility.

For peer review only

Table 2. Sensitivity analysis: cost-effectiveness of the 3D approach from an NHS/PSS perspective based on complete cases only

	**Usual care Mean (SE) N=592 complete cases**	**Intervention Mean (SE) N=599 complete cases**	**Incremental difference (95%CI)**
Unadjusted costs from the NHS/PSS perspective	4916 (290)	4757 (222)	
Adjusted costs from the NHS/PSS perspective	4905 (258)	4768 (256)	-137 (-852 to 577)
Unadjusted QALYs over 15 months of follow-up	0.698 (0.014)	0.750 (0.013)	
Adjusted QALYs over 15 months of follow-up	0.722 (0.005)	0.726 (0.005)	0.004 (-0.010 to 0.019)
ICER: Intervention dominates			
Net monetary benefit at £20,000: £222 (-584 to 1028)			

ICER, incremental cost-effectiveness ratio; CI, confidence interval; PSS, personal social services; QALY, quality-adjusted life year; SE, standard error. QALY estimates were adjusted for baseline utility.

For peer review only - http://bmjopen.bmj.com/site/about/guidelines.xhtml

Table 3. Costs and consequences of the 3D approach and usual care

**Costs and outcomes**	**Usual care**	***N***	**Intervention**	***N***	**Difference (95% CI)**
**Mean costs from the NHS perspective (£)**					
Practice-based consultations	627	754	726	715	99 (-7 to 205)
Practice-based investigations	45	755	61	717	15 (-6 to 37)
Community-based healthcare	160	615	167	601	7 (-35 to 49)
Inpatient stays	1867	766	1920	722	52 (-470 to 574)
Outpatient visits and day cases	614	766	613	722	-1 (-168 to 167)
Accident and emergency visits	102	766	99	722	-3 (-24 to 19)
Ambulance trips to hospital	131	615	141	601	10 (-56 to 77)
Prescribed medications	1230	755	1221	717	-8 (-220 to 203)
Pharmacy reviews	0	766	8	722	8 (7 to 9)
Intervention set up	0	797	4	749	4 (3 to 5)
Social services	559	615	403	601	-156 (-476 to 164)
All NHS/PSS	4929	609	4746	598	-183 (-923 to 556)
**Mean costs from the patient/carer perspective (£)**					
Prescription charges	5	755	4	717	-2 (-6 to 2)
Travel to GP practice	24	749	34	711	10 (-4 to 24)
Over-the-counter medications	39	615	35	601	-3 (-16 to 9)
Private healthcare	93	615	122	601	29 (-40 to 97)
All patient/carer	162	608	195	597	33 (-35 to 101)
**Mean societal productivity loss (£)**					
Productivity loss	122	608	161	597	39 (-47 to 125)
**Outcomes**					
QALYs (patient)	0.693	665	0.695	647	0.003 (-0.013 to 0.019)
QALYs (carer)	0.943	41	0.920	50	-0.024 (-0.064 to 0.017)
Deaths	32	749	46	797	p=0.18 (χ^2 test)

All costs and consequences are based on available data; the totals from each perspective are not, therefore, equal to the sum of the components. CI were calculated using standard errors from standard linear regressions adjusted for cluster at the level of the practice. QALYs were adjusted for baseline utility scores.

For peer review only - http://bmjopen.bmj.com/site/about/guidelines.xhtml

References

1. National Institute for Health and Care Excellence. Multimorbidity: clinical assessment and management 2016 [Available from: https://www.nice.org.uk/guidance/ng56.
2. Peters M, Kelly L, Potter CM, et al. Quality of life and burden of morbidity in primary care users with multimorbidity. *Patient Related Outcome Measures* 2018;9:103.
3. Li J, Green M, Kearns B, et al. Patterns of multimorbidity and their association with health outcomes within Yorkshire, England: baseline results from the Yorkshire Health Study. *BMC public health* 2016;16(1):649.
4. Marengoni A, Angleman S, Melis R, et al. Aging with multimorbidity: a systematic review of the literature. *Ageing research reviews* 2011;10(4):430-39.
5. Kasteridis P, Street A, Dolman M, et al. The importance of multimorbidity in explaining utilisation and costs across health and social care settings: evidence from South Somersets Symphony Project, 2014.
6. Wang L, Si L, Cocker F, et al. A Systematic Review of Cost-of-Illness Studies of Multimorbidity. *Applied health economics and health policy* 2017:1-15.
7. Smith SM, Wallace E, O'Dowd T, et al. Interventions for improving outcomes in patients with multimorbidity in primary care and community settings. *Cochrane Database of Systematic Reviews* 2016(3) doi: 10.1002/14651858.CD006560.pub3
8. Navickas R, Petric V-K, Feigl AB, et al. Multimorbidity: What do we know? What should we do? *Journal of comorbidity* 2016;6(1):4.
9. McPhail SM. Multimorbidity in chronic disease: impact on health care resources and costs. *Risk management and healthcare policy* 2016;9:143.
10. Hopman P, De Bruin SR, Forjaz MJ, et al. Effectiveness of comprehensive care programs for patients with multiple chronic conditions or frailty: a systematic literature review. *Health Policy* 2016;120(7):818-32.
11. Xu X, Mishra GD, Jones M. Evidence on multimorbidity from definition to intervention: an overview of systematic reviews. *Ageing research reviews* 2017;37:53-68.
12. Salisbury C, Man M-S, Bower P, et al. Management of multimorbidity using a patient-centred care model: a pragmatic cluster-randomised trial of the 3D approach. *The Lancet* 2018;392(10141):41-50.
13. Salisbury C, Man M-S, Chaplin K, et al. A patient-centred intervention to improve the management of multimorbidity in general practice: the 3D RCT. *Health Serv. Deliv. Res.* 2019;7:5. doi: 10.3310/hsdr07050
14. Man M-S, Chaplin K, Mann C, et al. Improving the management of multimorbidity in general practice: protocol of a cluster randomised controlled trial (The 3D Study). *BMJ open* 2016;6(4):e011261.
15. NHS Employers. Quality and outcomes framework [Available from: http://www.nhsemployers.org/your-workforce/primary-care-contacts/general-medical-services/quality-and-outcomes-framework accessed August 2018.
16. Herdman M, Gudex C, Lloyd A, et al. Development and preliminary testing of the new five-level version of EQ-5D (EQ-5D-5L). *Quality of life research* 2011;20(10):1727-36.
17. Van Hout B, Janssen M, Feng Y-S, et al. Interim scoring for the EQ-5D-5L: mapping the EQ-5D-5L to EQ-5D-3L value sets. *Value in Health* 2012;15(5):708-15.
18. National Institute for Health and Care Excellence. Position statement on use of the EQ-5D-5L valuation set 2017 [Available from: https://www.nice.org.uk/Media/Default/About/what-we-do/NICE-guidance/NICE-technology-appraisal-guidance/eq5d5l_nice_position_statement.pdf.

For peer review only - http://bmjopen.bmj.com/site/about/guidelines.xhtml

19. Curtis L, Burns A. Unit Costs of Health and Social Care 2016. University of Kent, Canterbury: Personal Social Services Research Unit 2016.
20. British National Formulary, March-September 2016. Basingstoke: Pharmaceutical Press/BMJ Group 2016.
21. Department of Health. NHS reference costs 2015 to 2016 2016 [Available from: https://www.gov.uk/government/publications/nhs-reference-costs-2015-to-2016.
22. Office for National Statistics. Annual Survey of Hours and Earnings: 2016 2016 [Available from: https://www.ons.gov.uk/employmentandlabourmarket/peopleinwork/earningsandworkinghours/datasets/agegroupashetable6.
23. HM Revenue and Customs. Travel - mileage and fuel rates and allowances Updated 6 April 2017 [Available from: https://www.gov.uk/government/publications/rates-and-allowances-travel-mileage-and-fuel-allowances/travel-mileage-and-fuel-rates-and-allowances.
24. NICE. Guide to the methods of technology appraisal 2013 2013 [Available from: https://www.nice.org.uk/process/pmg9/chapter/1-foreword.
25. Sterne JA, White IR, Carlin JB, et al. Multiple imputation for missing data in epidemiological and clinical research: potential and pitfalls. *Bmj* 2009;338:b2393.
26. Gomes M, Diaz-Ordaz K, Grieve R, et al. Multiple imputation methods for handling missing data in cost-effectiveness analyses that use data from hierarchical studies: an application to cluster randomized trials. *Med Decis Making* 2013;33(8):1051-63.
27. Díaz-Ordaz K, Kenward MG, Grieve R. Handling missing values in cost effectiveness analyses that use data from cluster randomized trials. *Journal of the Royal Statistical Society: Series A (Statistics in Society)* 2014;177(2):457-74. doi: 10.1111/rssa.12016
28. Stata Statistical Software: Release 14 [program]. College Station, TX: StataCorp LP., 2015.
29. Löthgren M, Zethraeus N. Definition, interpretation and calculation of cost-effectiveness acceptability curves. *Health Econ* 2000;9(7):623-30.
30. Manca A, Hawkins N, Sculpher MJ. Estimating mean QALYs in trial-based cost-effectiveness analysis: the importance of controlling for baseline utility. *Health Econ* 2005;14(5):487-96.
31. Mauskopf JA, Paul JE, Grant DM, et al. The Role of Cost—Consequence Analysis in Healthcare Decision—Making. *Pharmacoeconomics* 1998;13(3):277-88.
32. Husereau D, Drummond M, Petrou S, et al. Consolidated health economic evaluation reporting standards (CHEERS) statement. *Cost Effectiveness and Resource Allocation* 2013;11(1):6.
33. Janssen B, Szende A. Population norms for the EQ-5D. Self-reported population health: An international perspective based on EQ-5D: Springer 2014:19-30.
34. Katon W, Russo J, Lin EH, et al. Cost-effectiveness of a multicondition collaborative care intervention: a randomized controlled trial. *Archives of general psychiatry* 2012;69(5):506-14.
35. Barley EA, Walters P, Haddad M, et al. The UPBEAT nurse-delivered personalized care intervention for people with coronary heart disease who report current chest pain and depression: a randomised controlled pilot study. *PLoS One* 2014;9(6):e98704.
36. Mercer SW, Fitzpatrick B, Guthrie B, et al. The CARE Plus study–a whole-system intervention to improve quality of life of primary care patients with multimorbidity in areas of high socioeconomic deprivation: exploratory cluster randomised controlled trial and cost-utility analysis. *BMC medicine* 2016;14(1):88.
37. Office for National Statistics. Population Estimates for UK, England and Wales, Scotland and Northern Ireland 2016 [Available from: https://www.ons.gov.uk/peoplepopulationandcommunity/populationandmigration/populationestimates/datasets/populationestimatesforukenglandandwalesscotlandandnorthernireland.
38. NHS England. Enhanced service specification: Avoiding unplanned admissions: proactive case finding and patient review for vulnerable people 2016/17 2016 [Available from:

For peer review only - http://bmjopen.bmj.com/site/about/guidelines.xhtml

https://www.england.nhs.uk/commissioning/wp-content/uploads/sites/12/2016/04/aua-serv-spec.pdf.

39. Noble SM, Hollingworth W, Tilling K. Missing data in trial-based cost-effectiveness analysis: the current state of play. *Health economics* 2012;21(2):187-200.

For peer review only

Appendix 1

Table 1 Unit costs

Service		Unit cost	Notes	Source
**Social services**				
Social home care provided for social services		12	Independent sector home care provided for social services, face-to-face, sed on 30 minute visit	1
Daycare		61	Per client attendance	
Social worker		55	Based on 1 hour appointment	
Meals on wheels		7.03		2
**Prescription charges**				
Prescription charge per item		8.40		3
Prescription charge 3mo prepayment certificate		29.10		
Prescription charge 12mo prepayment certificate		104		
**GP practice services**				
GP appointment	Control arm	51.84	Per surgery consultation lasting 14.4 minutes (3D data)	1
	Intervention arm	48.60	Per surgery consultation lasting 13.5 minutes (3D data)	

For peer review only

Service		Unit cost	Notes	Source
GP home visit		100	Based on consultation lasting 15 minutes and 12 minutes travel time[4], plus 5 miles travel cost (NHS travel reimbursed at 56p per mile)	
GP phone call	Control arm	41.40	Based on consultation lasting 11.5 minutes (3D data)	
	Intervention arm	32.40	Based on consultation lasting 9 minutes (3D data)	
GP email consultation		21.60	Based on 6 minute consultation[5]	
Nurse appointment	Control arm	10.85	Based on 15.1 minute appointment (3D data), inflated from 14/15 costs[6]	
	Intervention arm	14.16	Based on 19.7 minute appointment (3D data), inflated from 14/15 costs[6]	
Nurse phone call	Control arm	4.24	Based on 5.9 minute call, inflated from 14/15 costs [6]	
	Intervention arm	5.03	Based on 7 minute call, inflated from 14/15 costs[6]	
Nurse home visit		22.21	Based on 15 minute visit, 12 minutes travelling, 5 miles travel costs (NHS travel reimbursed at 56p per mile)	
HCA face-to-face appointment	Control arm	3.45	Based on 9.4 minute appointment (3D data), band 2 nurse	
	Intervention arm	3.59	Based on 9.8 minute appointment (3D data), band 2 nurse	
HCA phone call (control arm)		5.87	Based on 16 minute call (3D data), band 2 nurse	

For peer review only

For peer review only - http://bmjopen.bmj.com/site/about/guidelines.xhtml

Service	Unit cost	Notes	Source
HCA home visit	12.70	Based on 15 minutes appointment, 12 minutes travelling, 5 miles travel costs, band 2 nurse (NHS travel reimbursed at 56p per mile)	
**Community services**			
Occupational Therapy visit	16	Based on band 5 occupational therapist, 30 minute appointment	1
Occupational Therapy home visit	25.20	Based on band 5 occupational therapist, 30 minute appointment, 12 minutes travelling, 5 miles travel costs (NHS travel reimbursed at 56p per mile)	
Occupational Therapy phone call	5.33	Based on band 5 occupational therapist, 10 minute call	
Speech and Language Therapy (SALT) visit	16	Based on band 5 SALT, 30 minute appointment	
Speech and Language Therapy home visit	25.20	Based on band 5 SALT, 30 minute appointment, 12 minutes travelling, 5 miles travel costs (NHS travel reimbursed at 56p per mile)	
Speech and Language Therapy telephone call	5.33	Based on band 5 SALT, 10 minute call	
Physiotherapy visit	16	Based on band 5 physiotherapist, 30 minute appointment	

For peer review only

Service	Unit cost	Notes	Source
Physiotherapy home visit	25.20	Based on band 5 physiotherapist, 30 minute appointment, 12 minutes travelling, 5 miles travel costs (NHS travel reimbursed at 56p per mile)	
Physiotherapy phone call	5.33	Based on band 5 physiotherapist, 10 minute call	
Chiropody / Podiatry visit	16	Based on band 5 podiatrist, 30 minute appointment	
Chiropody / Podiatry home visit	25.20	Based on band 5 podiatrist, 30 minute appointment, 12 minutes travelling, 5 miles travel costs (NHS travel reimbursed at 56p per mile)	
Chiropody / Podiatry phone call	5.33	Based on band 5 podiatrist, 10 minute call	
Community / District Nurse /Mental health nurse visit	22	Based on band 6 district nurse, 30 minute appointment	
Community / District Nurse /Mental health nurse home visit	46.8	Based on band 6 district nurse, 1 hour appointment including travel time, 5 miles travel costs (NHS travel reimbursed at 56p per mile)	
Community / District Nurse/ Mental health nurse phone call	7.33	Based on band 6 district nurse, 10 minute phone call	
NHS Counselling/Cognitive Behavioural Therapy visit	21	Based on band 6 counsellor, 30 minute appointment	

For peer review only - http://bmjopen.bmj.com/site/about/guidelines.xhtml

Service	Unit cost	Notes	Source
NHS Counselling/Cognitive Behavioural Therapy home visit	32.20	Based on band 6 counsellor, 30 minutes appointment, 12 minutes travelling, 5 miles travel costs (NHS travel reimbursed at 56p per mile)	
NHS Counselling/Cognitive Behavioural Therapy phone call	7	Based on band 6 counsellor, 10 minute call	
NHS 111 phone call	8.06	Maximum call cost of £7.80 in May 2013	[7]
NHS walk-in centre visit	41.74	Weighted mean of type 4 non-admitted, emergency medicine per attendance	[8]
OOH service visit	82.32	Average OOH hourly evening rate was £58.36 in 2005 compared with £36.75 for normal hours. Same differential applied to current GP in hours costs.	[9],[10]
OOH service home visit	158.80		
OOH service phone call	65.74		
**Ambulance services**			
Paramedic at home not involving a hospital visit	184	Ambulance services, see, treat and refer	[1]
Ambulance to hospital	238	See and treat and convey (including carbon 39 kgCO2e)	[1]
**Investigations (in outpatients)**			
MRI	155.64		[8]
DExa	71		
CT scan	104.88		

5

For peer review only

Service	Unit cost	Notes	Source
ECG	72		
Ultrasound	58.47		
Fluoroscopy	147.68		
**Investigations (directly accessed)**			
Cytology	16.88	Directly accessed pathology services	8
Histopathology and histology	30.77	Directly accessed pathology services	
Clinical Biochemistry	1.18	Directly accessed pathology services	
Haematology	3.10	Directly accessed pathology services	
Immunology	6.42	Directly accessed pathology services	
Microbiology	7.63	Directly accessed pathology services	
Other	3.13	Directly accessed pathology services	
MRI scan	147.25	Diagnostic imaging, directly accessed, weighted mean	
CT scan	107.52	Diagnostic imaging, directly accessed, weighted mean	
Ultrasound scan	52.82	Diagnostic imaging, directly accessed, weighted mean	
Bone scan	210.11	Nuclear medicine, directly accessed, weighted mean	
x-ray	30.26	Directly accessed diagnostic services, Plain film	
Dexa scan	68.29	Diagnostic imaging, direct access	
ECG	67.06	Diagnostic imaging, direct access, 19 years and over	
**Secondary care**			

6

For peer review only

Service	Unit cost	Notes	Source
Outpatient attendance	135.27	Weighted mean, consultant led	[8]
Day case	733.31	Weight mean of all daycases	
Elective inpatient	3749.81	Weighted mean	
Non-elective inpatient, long stay	3058.14	Weighted mean	
Non-elective inpatient, short stay	615.83	Weighted mean	
Elective excess bed day	361.67	Weighted mean	
Non-elective excess bed day	298.41	Weighted mean	
Individual HRGs		Mapped directly from Reference Costs file	
**Productivity losses**			
Median hourly wage, age 22-29	10.52	Excluding overtime, for employees on adult wages not affected by absence	[11]
Median hourly wage, age 30-39	13.57		
Median hourly wage, age 40-49	13.92		
Median hourly wage, age 50-59	13.18		
Median hourly wage, age 60+	11.26		

OOH: out-of-hours. HCA: health care assistant.

1. Curtis L, Burns A. Unit Costs of Health and Social Care 2016. University of Kent, Canterbury: Personal Social Services Research Unit 2016.
2. Curtis L. Unit Costs of Health and Social Care 2014. University of Kent, Canterbury: Personal Social Services Research Unit 2014.
3. Department of Health. NHS charges from April 2016 2016 [Available from: https://www.gov.uk/government/speeches/nhs-charges-from-april-2016.

For peer review only - http://bmjopen.bmj.com/site/about/guidelines.xhtml

4. Curtis L, Burns A. Unit Costs of Health and Social Care 2015. University of Kent, Canterbury: Personal Social Services Research Unit 2015.
5. Matheson C. Implementation of WebGP and e-consultations in Wessex GP practices. 2016
6. Bank of England. UK inflation calculator [Available from: http://www.bankofengland.co.uk/education/Pages/resources/inflationtools/calculator/default.aspx.
7. Health Committee. Written evidence from NHS Direct NHS Trust (ES 31) 2013 [Available from: https://www.publications.parliament.uk/pa/cm201314/cmselect/cmhealth/171/171vw25.htm.
8. Department of Health. NHS reference costs 2015 to 2016 2016 [Available from: https://www.gov.uk/government/publications/nhs-reference-costs-2015-to-2016.
9. O'Dowd A. Cost of out of hours care was 22% higher than predicted in England. *BMJ: British Medical Journal* 2006;332(7550):1113.
10. Curtis L, Netten A. Unit costs of health and social care: University of Kent; 2005 [
11. Office for National Statistics. Annual Survey of Hours and Earnings: 2016 2016 [Available from: https://www.ons.gov.uk/employmentandlabourmarket/peopleinwork/earningsandworkinghours/datasets/agegroupashetable6.

For peer review only - http://bmjopen.bmj.com/site/about/guidelines.xhtml

Appendix 2. Mean number of health and social care resource-use contacts over 15 months of follow-up.

Services	Usual care		Intervention	
	mean (SD)	*N*	mean (SD)	*N*
Inpatient stays	0.50 (1.05)	722	0.55 (1.09)	766
Inpatient nights	4.51 (16.6)	722	3.84 (11.94)	766
Outpatient visits	3.60 (4.00)	722	3.64 (3.73)	766
Day cases	0.15 (0.54)	722	0.14 (0.51)	766
Accident and emergency visits	0.63 (1.41)	722	0.61 (1.14)	766
Ambulance journeys	0.55 (1.83)	601	0.60 (3.50)	615
GP face-to-face consultations	7.27 (5.93)	715	8.70 (6.26)	754
GP home visits	0.64 (1.89)	715	0.79 (2.16)	754
GP phone calls	2.88 (5.00)	715	2.99 (4.53)	754
Nurse face-to-face consultations	5.40 (5.70)	715	7.93 (10.09)	754
Nurse home visits	0.06 (0.40)	715	0.09 (0.54)	754
Nurse phone calls	0.60 (1.56)	715	0.76 (1.63)	754
Healthcare assistant face-to-face consultations	2.42 (4.30)	715	3.55 (7.75)	754
Healthcare assistant GP home visits	0.00 (0.05)	715	0.08 (0.28)	754
Healthcare assistant GP phone calls	0.03 (0.17)	715	0.03 (0.17)	754
Prescribed medications	143.44 (130.57)	717	132.23 (117.36)	755
GP investigations	19.60 (16.52)	717	22.61 (16.99)	755
Pharmacist reviews		722	0.78 (0.42)	766
Occupational therapist clinic visits	0.18 (1.10)	601	0.2 (1.28)	615
Occupational therapist home visits	0.24 (1.29)	601	0.08 (0.5)	615
Occupational therapist phone calls	0.06 (0.53)	601	0.01 (0.14)	615
Speech and language therapist clinic visits	0.02 (0.33)	601	0.1 (1.03)	615
Speech and language therapist home visits	0.04 (0.69)	601	0 (0)	615
Speech and language therapist phone calls	0.00 (0.08)	601	0 (0)	615
Physiotherapist clinic visits	0.80 (2.74)	601	1.03 (2.67)	615
Physiotherapist home visits	0.16 (1.34)	601	0.15 (0.92)	615
Physiotherapist phone calls	0.00 (0.06)	601	0.01 (0.11)	615
Podiatrist clinic visits	1.26 (2.83)	601	1.19 (2.53)	615
Podiatrist home visits	0.13 (0.84)	601	0.21 (1.46)	615
Podiatrist phone calls	0.01 (0.13)	601	0.02 (0.49)	615
Community mental health nurse clinic contacts	0.27 (2.59)	601	0.08 (0.42)	615
Community mental health nurse home visits	0.21 (2.45)	601	0.35 (5.16)	615
Community mental health nurse phone calls	0.02 (0.23)	601	0.01 (0.13)	615

For peer review only

District nurse home visits	1.21 (4.08)	601	1.48 (6.54)	615
District nurse phone calls	0.03 (0.30)	601	0.05 (0.84)	615
Counsellor clinic visits	0.24 (2.34)	601	0.14 (0.99)	615
Counsellor home visits	0.01 (0.10)	601	0.01 (0.18)	615
Counsellor phone calls	0.00 (0.08)	601	0.00 (0.06)	615
NHS 111 phone calls	0.21 (0.85)	601	0.18 (0.57)	615
NHS walk-in centre visits	0.08 (0.41)	601	0.09 (0.50)	615
GP out-of-hours clinic visits	0.08 (0.34)	601	0.07 (0.28)	615
GP out-of-hours home visits	0.04 (0.33)	601	0.06 (0.56)	615
GP out-of-hours phone calls	0.02 (0.20)	601	0.03 (0.28)	615
Paramedic attendances at home	0.09 (0.43)	601	0.05 (0.29)	615
Other healthcare clinic visits	0.11 (0.88)	601	0.25 (3.24)	615
Other healthcare home visits	0.06 (0.65)	601	0.04 (0.41)	615
Other healthcare phone calls	0.01 (0.19)	601	0.01 (0.10)	615
Carer contacts	30.93 (148.26)	601	25.27 (135.78)	615
Daycare contacts	2.14 (17.53)	601	1.17 (10.50)	615
Meals on wheels	2.66 (28.32)	601	0.93 (18.98)	615
Social worker contacts	0.77 (11.35)	601	0.45 (3.98)	615

For peer review only

Reporting checklist for economic evaluation of health interventions

.

Based on the CHEERS guidelines.

Instructions to authors

Complete this checklist by entering the page numbers from your manuscript where readers will find each of the items listed below.

Your article may not currently address all the items on the checklist. Please modify your text to include the missing information. If you are certain that an item does not apply, please write "n/a" and provide a short explanation.

Upload your completed checklist as an extra file when you submit to a journal.

In your methods section, say that you used the CHEERS reporting guidelines, and cite them as:

Husereau D, Drummond M, Petrou S, Carswell C, Moher D, Greenberg D, Augustovski F, Briggs AH, Mauskopf J, Loder E. Consolidated Health Economic Evaluation Reporting Standards (CHEERS) statement.

		Reporting Item	Page Number
	#1	Identify the study as an economic evaluation or use more specific terms such as "cost-effectiveness analysis", and describe the interventions compared.	1

For peer review only

	#2	Provide a structured summary of objectives, perspective, setting, methods (including study design and inputs), results (including base case and uncertainty analyses), and conclusions	3
Background and objectives	#3	Provide an explicit statement of the broader context for the study. Present the study question and its relevance for health policy or practice decisions	5
Target population and subgroups	#4	Describe characteristics of the base case population and subgroups analysed, including why they were chosen.	5
Setting and location	#5	State relevant aspects of the system(s) in which the decision(s) need(s) to be made.	5
Study perspective	#6	Describe the perspective of the study and relate this to the costs being evaluated.	5
Comparators	#7	Describe the interventions or strategies being compared and state why they were chosen.	5-6
Time horizon	#8	State the time horizon(s) over which costs and consequences are being evaluated and say why appropriate.	5
Discount rate	#9	Report the choice of discount rate(s) used for costs and outcomes and say why appropriate	7
Choice of health outcomes	#10	Describe what outcomes were used as the measure(s) of benefit in the evaluation and their relevance for the type of analysis performed	6

For peer review only

Meaurement of effectiveness	#11a	Single study-based estimates: Describe fully the design features of the single effectiveness study and why the single study was a sufficient source of clinical effectiveness data	5
Measurement of effectiveness	#11b	Synthesis-based estimates: Describe fully the methods used for identification of included studies and synthesis of clinical effectiveness data	n/a
Measurement and valuation of preference based outcomes	#12	If applicable, describe the population and methods used to elicit preferences for outcomes.	6
	#13a	Single study-based economic evaluation: Describe approaches used to estimate resource use associated with the alternative interventions. Describe primary or secondary research methods for valuing each resource item in terms of its unit cost. Describe any adjustments made to approximate to opportunity costs	6-7
Estimating resources and costs	#13b	Model-based economic evaluation: Describe approaches and data sources used to estimate resource use associated with model health states. Describe primary or secondary research methods for valuing each resource item in terms of its unit cost. Describe any adjustments made to approximate to opportunity costs.	n/a
Currency, price date,	#14	Report the dates of the estimated resource quantities and	7

For peer review only

and conversion		unit costs. Describe methods for adjusting estimated unit costs to the year of reported costs if necessary. Describe methods for converting costs into a common currency base and the exchange rate.	
Choice of model	#15	Describe and give reasons for the specific type of decision analytical model used. Providing a figure to show model structure is strongly recommended.	n/a
Assumptions	#16	Describe all structural or other assumptions underpinning the decision-analytical model.	n/a
Analytical methods	#17	Describe all analytical methods supporting the evaluation. This could include methods for dealing with skewed, missing, or censored data; extrapolation methods; methods for pooling data; approaches to validate or make adjustments (such as half cycle corrections) to a model; and methods for handling population heterogeneity and uncertainty.	8-9
Study parameters	#18	Report the values, ranges, references, and, if used, probability distributions for all parameters. Report reasons or sources for distributions used to represent uncertainty where appropriate. Providing a table to show the input values is strongly recommended.	n/a
Incremental costs and outcomes	#19	For each intervention, report mean values for the main categories of estimated costs and outcomes of interest, as well as mean differences between the comparator groups.	18

For peer review only

		If applicable, report incremental cost-effectiveness ratios.	
Characterising uncertainty	#20a	Single study-based economic evaluation: Describe the effects of sampling uncertainty for the estimated incremental cost and incremental effectiveness parameters, together with the impact of methodological assumptions (such as discount rate, study perspective).	10
	#20b	Model-based economic evaluation: Describe the effects on the results of uncertainty for all input parameters, and uncertainty related to the structure of the model and assumptions.	n/a
Characterising heterogeneity	#21	If applicable, report differences in costs, outcomes, or cost effectiveness that can be explained by variations between subgroups of patients with different baseline characteristics or other observed variability in effects that are not reducible by more information.	10
Study findings, limitations, generalisability, and current knowledge	#22	Summarise key study findings and describe how they support the conclusions reached. Discuss limitations and the generalisability of the findings and how the findings fit with current knowledge.	11-13
Source of funding	#23	Describe how the study was funded and the role of the funder in the identification, design, conduct, and reporting of the analysis. Describe other non-monetary sources of support	1
Conflict of interest	#24	Describe any potential for conflict of interest of study	2

For peer review only

contributors in accordance with journal policy. In the absence of a journal policy, we recommend authors comply with International Committee of Medical Journal Editors recommendations

The CHEERS checklist is distributed under the terms of the Creative Commons Attribution License CC-BY-NC. This checklist can be completed online using https://www.goodreports.org/, a tool made by the EQUATOR Network in collaboration with Penelope.ai

For peer review only

Improving the Management of Multimorbidity using a Patient-Centred Care Model: Pragmatic Cluster Randomised Trial of the 3D Approach

This supplement contains the following items:

Original protocol 2
Summary of changes to protocol and trial registry 55

Please note:

- Versions 1 to 5 of the protocol were for the external pilot study conducted before this trial and not reported here. Version 5.1 is the original protocol used at the beginning of the main trial as reported here.
- Changes to the protocol are described on p55; these were all approved by the Research Ethics Committee.

Some of this material is published in adapted form in ©Man M, Chaplin K, Mann C, et al Improving the management of multimorbidity in general practice: protocol of a cluster randomised controlled trial (The 3D Study) BMJ Open 2016;6:e011261. doi: 10.1136/bmjopen-2016-011261. Reproduced under the terms of the Creative Commons Attribution (CC BY 4.0) license.

For peer review only - http://bmjopen.bmj.com/site/about/guidelines.xhtml

Original protocol

IMPROVING THE MANAGEMENT OF PATIENTS WITH MULTIMORBIDITY IN GENERAL PRACTICE

For peer re

The 3D Study: Improving whole person care

**Funder**	NIHR Health Services and Delivery Research Programme (HS&DR)
**Funder Ref Number**	12/130/15
**Sponsor**	University of Bristol
**Sponsor Ref Number**	2148
**REC number**	14/SW/0011
**ISRCTN number**	ISRCTN06180958
**CSP number**	139520
**Chief Investigator**	Professor Chris Salisbury
**Version number, Date**	V5.1; 13/04/15

For peer review only - http://bmjopen.bmj.com/site/about/guidelines.xhtml

1 History of version changes and amendments

NB Versions 1 to 5 of the protocol were for the pilot study conducted before this trial. This was an external pilot. Version 5.1 is the original protocol used at the beginning of the main trial as reported here. Version 8 was the final protocol.

Version No.	Version Date	Pages	Description of change

See summary of changes to protocol and registry, page 55

For peer review only

For peer review only - http://bmjopen.bmj.com/site/about/guidelines.xhtml

Contents

IMPROVING THE MANAGEMENT OF PATIENTS WITH MULTIMORBIDITY IN GENERAL PRACTICE 2
1 History of version changes and amendments 3
2 Trial Investigators.. 7
3 Abbreviations 8
4 Introduction 10
4.1 Summaries 10
4.1.1 Lay Summary.. 10
4.1.2 Scientific Summary of research.. 11
4.2 Background 14
4.3 Rationale 16
4.4 Study Aims and Objectives... 16
5 Trial Design... 17
5.1 Study Design... 17
5.1.1 Study outline 17
5.1.2 Design... 17
5.1.3 Theoretical/Conceptual Framework 17
5.2 Study setting 18
5.3 Eligibility criteria for Practices.. 18
5.4 Eligibility criteria for participants... 18
5.4.1 Inclusion Criteria 18
5.4.2 Exclusion Criteria.. 19
5.5 Recruitment procedures 20
5.5.1 Recruitment of General Practices 20
5.5.2 Identification and consent of patients... 20
5.5.3 Permission of Patient's Carers 21
5.5.4 Baseline data collection 21
5.6 Randomisation 22
5.6.1 Control group... 22
5.6.2 Blinding 22
5.7 Procedure optimisation and pilot 22
5.8 Follow-up procedures 22
5.9 Withdrawal... 23
5.9.1 Practice Withdrawal... 23

For peer review only - http://bmjopen.bmj.com/site/about/guidelines.xhtml

5.9.2 Participant withdrawal ... 23
6 Intervention ... 24
7 Data Collection ... 26
7.1 Quantitative Outcome Measures ... 26
7.1.1 Primary Outcome ... 26
7.1.2 Secondary Outcomes ... 26
7.1.3 Socio-demographic measures ... 27
7.1.4 Carer outcomes ... 27
7.1.5 Resource use ... 27
7.2 Data Management ... 28
7.2.1 Source data ... 28
7.2.2 Data Storage ... 29
8 Data Analysis of quantitative trial outcomes ... 29
8.1 Sample Size calculation ... 29
Efficacy data analysis ... 29
8.1.1 Primary analysis ... 30
8.1.2 Secondary and subgroup analysis ... 30
8.1.3 Use of anonymised data ... 30
9 Economic Evaluation ... 30
10 Process Evaluation ... 31
10.1 Background ... 31
10.2 Design ... 32
10.3 Methods and Setting ... 32
10.4 Sampling ... 32
10.5 Recruitment ... 33
10.6 Data Collection for Process evaluation ... 33
10.6.1 Quantitative data ... 33
10.6.2 Qualitative data ... 33
10.7 Commissioners ... 35
10.8 Data Analysis ... 36
10.9 Unintended consequences ... 37
11 Research Governance ... 37
11.1 Ethical conduct ... 37
11.2 Safety Assessment and Adverse Events ... 38

For peer review only

For peer review only - http://bmjopen.bmj.com/site/about/guidelines.xhtml

11.2.1 Definitions ... 38
11.2.2 Detecting and recording AEs and SAEs ... 39
11.2.3 Assessment of relatedness and expectedness ... 39
11.2.4 Reporting requirements for SAEs ... 40
11.3 Monitoring ... 40
11.4 Patient and Public Involvement ... 40
11.5 Confidentiality ... 41
11.6 Data Protection ... 41
12 Trial Management ... 41
12.1 Funding ... 41
12.2 Sponsorship ... 41
12.3 Insurance and Indemnity ... 42
12.4 Trial Management Group (TMG) ... 42
12.5 Independent Trial Steering Committee (TSC) ... 42
12.6 Data Monitoring Committee (DMC) ... 42
12.7 Advisory Group ... 42
12.8 Investigator responsibilities ... 42
13 Dissemination and projected outputs ... 43
13.1 Dissemination to Patients ... 44
13.2 Dissemination to Health Care Professionals ... 44
13.3 Dissemination to Commissioners and Policy Makers ... 44
13.4 Dissemination to academics ... 44
14 Study conduct and end of study responsibilities ... 44
14.1 Protocol Amendment ... 45
14.2 Protocol Violations and Deviations ... 45
14.3 End of study Archiving ... 45
14.4 End study ... 45
15 References ... 46
16 Appendix A – Trial Contacts ... 50
Appendix B – Trial Timeline ... 52
17 Appendix C – Study Flow Diagram & Conceptual Framework ... 53
18 Appendix D – Participant flowchart ... 54
Amendments to the study protocol, all approved by ethics committee ... 55
Changes to trial registry: ISRCTN06180958 ... 56

For peer review only

For peer review only - http://bmjopen.bmj.com/site/about/guidelines.xhtml

2 Trial Investigators

See Appendix A for full contact details

Chief Investigator

Chris Salisbury, University of Bristol

Principal Investigators

Stewart Mercer, University of Glasgow

Pete Bower, University of Manchester

Co-applicants

Bruce Guthrie, University of Dundee

Sandra Hollinghurst, University of Bristol

Sara Brookes, University of Bristol

Ali Heawood, University of Bristol

Cindy Mann, University of Bristol

Imran Rafi, Royal College of General Practitioners

Ardiana Gjini, Public Health, NHS England

For peer review only

3 Abbreviations

AE	Adverse Events
BHF	British Heart Foundation
BNF	British National Formulary
CARE	The Consultation And Relational Empathy measure
CBT	Cognitive Behavioural Therapy
CCG	Clinical Commissioning Group
CI	Confidence Interval
CKD	Chronic Kidney Disease
CLRN	Comprehensive Local Research Network
CONSORT	CONsolidated Standards Of Reporting Trials
COPD	Chronic Obstructive Pulmonary Disease
CRF	Case Report Form
CRN	Clinical Research Network
CVD	Cardiovascular Disease
DMC	Date Monitoring Committee
EQ-5D	Euroquol 5D Questionnaire
GCP	Good Clinical Practice
GP	General Practitioner
HADS	Hospital Anxiety Depression Scale
ICC	Intra Cluster Coefficient
LTC	Long Term Conditions
MMAS	Morisky Medication Adherence Scale
NHS	National Health Service
NICE	National Institute for Health and Clinical Excellence
NIHR	National Institute for Health Research
OR	Odds ratio
PACIC	Patient Assessment of Chronic Illness Care measure
PHQ-9	Patient Health Questionnaire
PI	Principal Investigator
PPI	Public and Patient Involvement
PSS	Personal and Social Services
QALY	Quality Adjusted Life Years
QOF	Quality and Outcomes Framework
QoL	Quality of Life
R&D	Research and Development
RCGP	Royal College of General Practitioners
RCT	Randomised Controlled Trial
REC	Research Ethics Committee
RM&G	Research Management and Governance
SAE	Serious Adverse Event
SMI	Serious Mental Illness
SOP	Standard Operating Procedure
SSC	Service Support Costs
TMG	Trial Management Group
TSC	Trial Steering Committee

For peer review only

For peer review only - http://bmjopen.bmj.com/site/about/guidelines.xhtml

WISE study	Whole systems Informing Self-management Engagement study

For peer review only

4 Introduction

4.1 Summaries

4.1.1 Lay Summary

An increasing proportion of the population have long term health conditions. The current approach to managing patients with long term conditions is based on guidelines and treatment for each specific condition. Patients are regularly called to clinics in general practice (eg. diabetes clinics) to see a nurse who follows guidelines on managing that particular disease. However, there is an increasing awareness that many patients have multiple long term health conditions. This is known as multimorbidity. Patients with multimorbidity often have to attend several clinics which can be repetitive, inconvenient and inefficient. They see different nurses and doctors who may give conflicting advice. These patients may have to take a large number of drugs which can be confusing, difficult to remember and some combinations could be potentially dangerous. These patients frequently get depressed and they also sometimes complain that no-one treats them as a 'whole person' or takes their views into account.

To address this problem, this study aims to develop and test a new approach to how GP practices manage patients with multimorbidity. Instead of focussing on each disease in isolation, the aim is to treat the whole patient in a consistent, joined-up manner in order to improve their overall quality of life. GP practices testing this new management system (intervention group) will be compared with GP practices following the current treatment system (usual care or control group).

Specifically, patients in general practices allocated to the intervention group will be identified and flagged on their GP computer systems. A named nurse and doctor will be allocated to them to manage their care. These patients will be given a card to identify them to reception staff who will offer longer appointment times. Patients will also be invited for a comprehensive '3D' health review every 6 months designed to cover all of their health issues. The patient will first see their named nurse who will identify the patients' concerns and priorities (**Dimensions of health**), as well as perform any routine checks required by each of the patients' conditions. The patients' **Drugs** will be reviewed to check they are being correctly prescribed (without dangerous interactions), taken properly and where possible, to try to simplify the patients prescriptions e.g. by arranging for all of them to be taken once a day. The named GP will check for and treat symptoms of **Depression**. The practice will also have a linked 'general physician' at the local hospital whom they can contact easily for advice about patients with complex problems.

Patients in general practices allocated to the usual care group will continue having their care managed by their GPs and practice nurses using current management practices of multiple appointments and clinics for each separate condition.

We will first try out the new approach in three practices and use this experience to improve it. Then we aim to recruit 32 GP practices from in and around Bristol, Manchester and Glasgow to take part in the main study. These practices will be randomly assigned to the intervention or usual care group. A target of 1383 multimorbid patients from these practices will be followed up over a period of 12 months.

Before the practice randomisation and at 6 and 12 months into the study, participants from both groups will be asked to fill in questionnaires about their wellbeing, illnesses and treatments, their experience of their care, and what health resources they use. The research team will review the

For peer review only - http://bmjopen.bmj.com/site/about/guidelines.xhtml

notes of patients in both groups to record the number, type, duration and quality of consultations within general practice as well as their use of other health and social services.

We will compare the cost of the old and new approaches (both the cost to the NHS and the cost to the patient) and relate this to the benefit in a cost-effectiveness analysis.

We will check whether the care provided in the intervention practices actually changed the way intended. Through interviews with patients and care staff, and by direct observation, we will explore how well the approach was implemented and how it could be improved.

4.1.2 Scientific Summary of research

AIM: The aim is to optimise, implement and evaluate an intervention to improve the management of patients with multimorbidity in general practice

Design: Pragmatic cluster randomised controlled trial with nested process evaluation and economic analysis of cost effectiveness.

Setting: 3 practices (2 in Bristol and 1 in Manchester) will participate in a pilot phase followed by approximately 32 GP practices in Bristol, Glasgow and Manchester. Only practices with a minimum of 3 GP partners and minimum practice list size of 4,500 patients will be approached in order to ensure an adequate number of participants per practice.

Participants:

A target of 1383 participants from 32 GPs will be recruited to the study. Eligible participants will be 18 or over with multimorbidity, defined as having three or more long term conditions (LTC) from a predefined list of conditions.

Interventions

GP practices will be randomised to the intervention or usual care groups.

A. Intervention group:

This is also described as the 'new approach' group. The intervention is designed to address the problems of illness burden (poor quality of life, depression) treatment burden (multiple unco-ordinated appointments, polypharmacy, poor primary/secondary care co-ordination) and lack of patient-centred care (low continuity, disregard of patients' priorities) experienced by patients with multimorbidity.

There are 4 main components of the intervention

1. Identification and prioritisation of patients with multimorbidity – given '3D' card + flagged records.
2. Improving continuity of care – defined usual GP and usual practice nurse. Offered longer appointment times.
3. Reducing the burden of illness and treatment – comprehensive assessment every 6 months. Each 3D assessment consists of 2 appointments approximately 1 week apart. At the first appointment, the patients' usual nurse will complete a bespoke computerised template to address all of the 3D elements, collect relevant data in relation to the

For peer review only - http://bmjopen.bmj.com/site/about/guidelines.xhtml

patient's combination of LTCs, and organise necessary tests. At the second appointment, the usual GP will review all the information, conduct a thorough review of medication and agree an written care plan with the patient for them to take away. The '3D' label provides a mnemonic for health professionals. It refers to:

- Dimensions of Health – patient's concerns and priorities, addressing quality of life issues first
- Depression – Assessment and treatment
- Drugs – Doctors will seek to simplify treatment regimes and improve medication adherence, aided by prior pharmacist review of patient's medication regime

4. Improving integration – each practice has a designated general physician who is readily available to discuss multimorbidity patients with complex needs and help co-ordinate hospital investigations.

To ensure the intervention is effectively implemented, it will be incentivised as if it were an Enhanced Service or included in the Quality and Outcomes Framework (QOF), with payment against targets for completion of two 3D assessments per annum. Practices will train in local collaboratives to share ideas. We will allow local adaptation, appoint a practice champion, provide training and monthly feedback on performance e.g. number of patients with care plans.

B. Control group:

Practices in the care as usual group, will continue their usual practice following disease specific computerised protocols focussing on data related to QOF targets.

<u>Outcomes</u>

Outcomes will be collected at baseline, 6 months and 12 month post-randomisation.

The primary outcome will be Health Related Quality of Life as measured by the EQ-5D-5L at 12 months post randomisation.

Secondary outcome measures will include:

Burden of illness measures:

- Self-rated health
- Bayliss measure of illness burden in multimorbidity
- Quality of disease management (composite measure of QOF achievement)
- Hospital Anxiety Depression Scale (HADS)

Burden of Treatment measures:

- Brief Treatment Burden Questionnaire
- Morisky Medication Adherence Scale (MMAS, 8 item)
- Number of drugs prescribed
- Reduction in high risk drug combinations

For peer review only - http://bmjopen.bmj.com/site/about/guidelines.xhtml

Experience of patient centred care:

- CARE measure and how well you know your doctor
- Coordination of care and care related to patients priorities (questions from LTC6 QIPP)
- Management of LTCs (PACIC)
- Overall satisfaction (single item)

Use of resources:

- Hospital admission and outpatient rates
- Use of health services in primary, community and secondary care
- Investigations
- Prescribed medication

Socio-demographic measures (measured at baseline only):

- Number of long term conditions
- Age
- Gender
- Education
- Ethnicity
- Deprivation status
- Work status

Measures of strain on patient's carers:

- Carer Experience Scale
- EQ-5D-5L completed by carers.
- Brief Treatment Burden Questionnaire for carers

For peer review only

Alongside the trial, a parallel mixed methods process evaluation combining quantitative and qualitative methods will be conducted in order to provide understanding of trial delivery, intervention implementation, current management practices and the responses of targeted participants.

An economic evaluation will be undertaken from the perspectives of (a) NHS and personal social services (PSS) and (b) patients. In a cost-consequences analysis we will relate the cost of the intervention or usual care to changes in a range of outcomes. In a cost-effectiveness analysis from the NHS and PSS perspective we will estimate the incremental cost per QALY gain. Uncertainty will be addressed in sensitivity analyses and by using bootstrapping to estimate the net monetary benefit and a cost-effectiveness acceptability curve.

Sample size: based on 32 general practices, 108 eligible patients per practice, 40% providing consent, we aim to recruit 1383 patients (1555 including pilot practices). With 80% follow-up at 12

For peer review only - http://bmjopen.bmj.com/site/about/guidelines.xhtml

months, and ICC at practice level of 0.03, the study will have 90% power to detect a difference between trial arms of 0.274 SDs in the EQ-5D.

Benefits and potential impacts: There is widespread interest in how to improve care for patients with multimorbidity. If successful, this intervention could improve the quality of life of patients and their experience of care and reduce NHS and patient costs. By working with RCGP, we will ensure that if the intervention is effective, it can be implemented widely, quickly and effectively.

4.2 Background

In an attempt to improve the quality of care of individual long term conditions in general practice, care has become increasingly driven by protocols delivered using computerised templates by practice nurses. These nurses often have extra training in specific diseases and provide care within disease-specific clinics (e.g. diabetic clinics) which focus on one disease at a time. Primary care clinicians are incentivised through the QOF to achieve against targets relating to a limited range of specific long term conditions. Disease pathways for specific long term conditions are being developed to improve vertical integration across primary and secondary care.

These developments fail to take account of the fact that many people have multiple long term conditions (multimorbidity) [1,2]. Sometimes these co-morbid conditions have a bigger impact on the patient's quality of life than the specific condition being addressed at a nurse led chronic disease management clinic. The priorities and incentives for the health professionals dealing with a specific disease may or may not align with the priorities of the patient [3].

As the population ages this problem is becoming increasingly important, since multimorbidity is much more common in older people [1,2]. Multimorbidity matters because people who have multiple conditions have poor quality of life [4], increased morbidity [5], reduced life-expectancy [6], and increased rates of depression [7]. Patients with multimorbidity are also a priority for the health service because they account for a high proportion of resource use in both primary and secondary care (including having high rates of hospital admissions) [8-10].

Failing to improve care for multimorbidity will also lead to increased inequalities in health. Multimorbidity is more common in deprived areas [2], and patients with fewer material and personal resources are particularly disadvantaged by having to attend multiple appointments for each of their long term conditions, and being expected to follow a series of different care plans [11,12]. Their care is also more likely to be complicated by other medical and social factors, such as poor mental health, poor housing, and smoking [13].

The consequences of a single disease approach for the health service potentially include duplication and also gaps in services (e.g. conditions included in the QOF are prioritised but others are neglected) [14], inefficiency (because the same topics are addressed repeatedly by different specialist practice nurses), and waste (because of non-adherence to medication and non-attended appointments) [15]. If taken to its logical conclusion, the disease pathways approach would mean that one patient with multimorbidity would have their care managed by several specialist services (each crossing primary and secondary care), but there would be little co-ordination between these specialist services and no one professional who has an overview and takes responsibility for the patient as a whole.

For patients with multimorbidity, the single disease approach is inconvenient and inefficient, because they are repeatedly invited to different disease-focused appointments in general practice, where they are asked the same questions and given the same advice (or sometimes conflicting

For peer review only - http://bmjopen.bmj.com/site/about/guidelines.xhtml

advice which can be confusing) [3,12,16,17]. Patients may receive less good quality care if the specialist nurse is not aware of the impact of the treatment of one disease on other diseases, which can be a particular problem with drug interactions. Alternatively, the disease-focused nurse or doctor may slavishly follow guidelines without recognising that the evidence underlying those guidelines is not necessarily applicable to the individual patient in front of them with multimorbidity (since most guidelines are based on research which excluded patients with comorbidities) [18,19].

If all the recommendations for each long term condition are considered in isolation and followed, patients with multimorbidity are likely to have numerous investigations and to be prescribed large numbers of drugs [20,21]. This polypharmacy can be burdensome for patients, increases the likelihood of interactions and adverse effects (including those causing hospital admissions), and may reduce medication adherence [19,22-24].

It is also well recognised that multimorbidity is associated with an increased prevalence of depression [7]. This association is strongest in deprived areas [2]. The relationship between physical and mental health works in both directions – people with chronic illnesses are more likely to be depressed, and those who are depressed are less likely to manage their long term conditions well, leading to worse disease control and poorer health outcomes [25]. People with multimorbidity and depression are also more likely to have unplanned hospital admissions [9]. The prevalence of comorbid depression and physical health problems is much higher in deprived areas than affluent areas [2]. Therefore improving mental health in people with multimorbidity is also a priority.

Most importantly, patients with multimorbidity can feel that no one treats them as a ‘whole person’ rather than ‘a disease’ [3]. Patients say that they want to have a relationship with one health professional that they can trust, and who listens to them, helping them make appropriate decisions in the context of their life circumstances and values [3]. Given the large number of health problems they face and the number of potentially relevant investigations and treatments, they may want to set priorities and make trade-offs so that medication regimes are not excessively burdensome and they are not over-investigated. For patients, improving quality of life (which might include not spending too much time in contact with the health service or suffering side-effects of medication) might be a higher priority than achieving improved indicators of disease control with a view to greater longevity.

In summary, patients with multimorbidity experience problems of illness burden (poor quality of life, depression) treatment burden (multiple unco-ordinated appointments, polypharmacy) and lack of person-centred care (low continuity, little attention paid to patients’ priorities). This research is designed to test the hypothesis that an intervention in general practice designed to address the needs and priorities of patients with multimorbidity will improve their health related quality of life (primary outcome), reduce their burden of illness and treatment and improve their experience of care, while being more cost-effective than conventional service models. This will be tested using a cluster RCT, with economic evaluation and mixed methods process evaluation.

For peer review only - http://bmjopen.bmj.com/site/about/guidelines.xhtml

4.3 Rationale

There is a growing body of literature and commentary on the scale and consequences of multimorbidity [1,2,5,13,15,19,21,26]. Commissioners, professional bodies, academics and other stakeholders have all recognised the growing tension between the single disease focus of medicine and the needs of patients with multiple long term conditions [27]. This is evidenced by recent reports from the Royal College of General Practitioners [28], the Royal College of Physicians [29], NICE [30], and others [31]. There is a long term challenge to redesign the NHS to reflect the needs of patients with multiple long term conditions[32] in light of the ageing population.

A recent Cochrane review however, highlighted the paucity of research on interventions to improve the outcomes of patients with multimorbidity in primary care[33]. Ten studies were identified examining a range of complex interventions which demonstrated mixed effects. The interventions that proved most effective were organisational interventions focussed on areas of concern for patients or where they have difficulties, such as functional ability and medication management. No studies included an economic analysis of cost effectiveness although a trend towards improved prescribing and medication adherence suggests the potential for cost savings. The conclusions of the systematic review called for further pragmatic studies based in primary care settings, using clear definitions of participants and appropriate outcomes.

There has been considerable research on the scale of the problem and the needs of patients with multimorbidity [3,11,26,34-37]. Qualitative studies of patients with multimorbidity have identified several barriers to their self management (including depression, poor patient-clinician communication, poor physical function and greater financial constraints [11])as well as identifying what services these patients desire (including greater access, individualised care plans, continued care by a single coordinator of care who will listen and respond to their shifting priorities [3]).

There are isolated examples of individual general practices trying out some of the ideas described above, e.g. co-ordinated assessments, although these typically focus on rationalising appointments rather than the extended assessment and holistic approach proposed here[38]. There is a pressing need for rigorous research to test the benefits and costs of a new approach to managing multimorbidity.

The issues identified by the qualitative studies were confirmed by patient and carer forum meetings held prior to the development of the current study proposal and which informed the design of the intervention for the current study. The resulting complex healthcare intervention requires a large scale randomised controlled trial to evaluate the clinical effectiveness, cost effectiveness and implementation of this service.

4.4 Study Aims and Objectives

AIM: The aim is to optimise, implement and evaluate an intervention to improve the management of patients with multimorbidity in general practice

HYPOTHESIS: An intervention in general practice designed to improve the management of multimorbidity will improve patients' health related quality of life, reduce burden of illness and treatment and improve their experience of care whilst being more cost-effective than conventional service models.

OBJECTIVES:

For peer review only - http://bmjopen.bmj.com/site/about/guidelines.xhtml

- To optimise an intervention to improve the management of multimorbidity in general practice through **piloting** in three practices.
- To implement this intervention in a representative range of general practices
- Through a **cluster randomised controlled trial** and **economic evaluation**, to assess the impact on health related quality of life, illness burden, treatment burden, patient experience, carer's burden and quality of life, and cost-effectiveness.
- Through **mixed methods process evaluation**, to explore how and to what extent the intervention was implemented, the advantages and disadvantages of different models of care for patients with multimorbidity, and how and why the intervention was or was not beneficial. We will also characterise usual care and explore any changes to management practices over the duration of this study in usual care GP practices.
- To **design educational materials and commissioning guides** to ensure that the intervention is delivered consistently in practices in the trial and that if beneficial, it can be speedily rolled out nationally.

5 Trial Design

5.1 Study Design

5.1.1 Study outline

This is a multi-centre pragmatic, two-arm, practice-level cluster randomised controlled trial, with parallel mixed methods process evaluation and economic analysis of cost effectiveness.

5.1.2 Design

The overall design is a pragmatic cluster randomised controlled trial comparing a new approach to the management of multimorbidity in general practice versus usual care, with process evaluation and economic analysis of cost-effectiveness.

In line with the MRC framework for the evaluation of complex interventions [39], we will first conduct a pilot and optimisation phase and then conduct a parallel mixed methods process evaluation alongside the trial to examine how the intervention is implemented by intervention practices and to characterise usual care. This will help to gain understanding about why the intervention worked (or did not work), and will inform and facilitate commissioning and other implementation of the intervention if appropriate.

Economic evaluation will be undertaken from the perspectives of (a) NHS and personal social services (PSS) and (b) patients. In a cost-consequences analysis we will relate the cost of the intervention or usual care to changes in a range of outcomes; cost-effectiveness analysis from the NHS and PSS perspective will estimate the incremental cost per QALY gain.

5.1.3 Theoretical/Conceptual Framework

For peer review only

For peer review only - http://bmjopen.bmj.com/site/about/guidelines.xhtml

The underlying theoretical basis for the intervention is the Patient Centred Care Model, described by Stewart et al [40]. This is strongly valued by patients[41] and there is some evidence that it is associated with improved health outcomes [42-45]. A recent report from the American Geriatric Society has recommended the Patient Centred Care Model to improve care for multimorbidity [31].

The concept of patient centred care has been reviewed and developed by later authors [46,47] but it broadly includes four key components:

- A focus on the patient's *individual disease and illness experience*: exploring the main reasons for their visit, their concerns and need for information.
- A *biopsychosocial perspective*: seeking an integrated understanding of the whole person, including their emotional needs and life issues.
- Finding *common ground* on what the problem is and mutually agreeing management plans.
- Enhancing the *continuing relationship* between the patient and doctor (the therapeutic alliance).

As shown in the flow diagram in Appendix C, the conceptual framework for our intervention draws on the existing research evidence about the main types of problems experienced by patients with multimorbidity and their preferences for care, and uses strategies based on the Patient Centred Care Model to seek to address these problems. For example, there is evidence that patients with long term conditions particularly value relational continuity of care[48], therefore the intervention includes strategies to improve relational continuity and one of the outcome measures (CARE) collects data about whether this is achieved.

Our conceptual framework also draws on the Chronic Care model[49] and experience in related initiatives such as Year of Care [50], for example the importance of promoting patient engagement in self-care through care plans, improving communication between primary and secondary care, and the recognition that organisational change needs to be accompanied by attitudinal change amongst clinicians, enhanced by training, appointment of local GP champions, collaborative working, incentives, support and feedback.

5.2 Study setting

This study is based in primary care involving General Practices serving different patient populations in 3 geographical areas; in and around Bristol, Manchester and Glasgow. Practices will be selected from areas within a range of socioeconomic deprivation levels as well as urban, suburban and rural areas.

5.3 Eligibility criteria for Practices

General practices with the following criteria will be approached:

- A minimum of three GP partners
- Minimum practice list size of 4,500 patients
- Uses EMIS GP computer system

5.4 Eligibility criteria for participants

5.4.1 Inclusion Criteria

- Aged 18 and over (on date of invitation to participate)

For peer review only - http://bmjopen.bmj.com/site/about/guidelines.xhtml

- Three or more long term conditions from the following list of conditions:
  - Cardiovascular disease (CVD) or Chronic kidney disease (CKD): including coronary heart disease, hypertension, heart failure, peripheral arterial disease, chronic kidney disease stage 3 to 5.
  - Stroke
  - Diabetes
  - COPD or asthma
  - Epilepsy
  - Atrial fibrillation
  - Severe mental health problems (eg. Schizophrenia, psychotic illnesses)
  - Depression
  - Dementia
  - Learning disability
  - Rheumatoid arthritis

In the above list we have grouped a number of cardiovascular conditions together, along with CKD, as management of them is very similar, and similarly grouped asthma and COPD together. Therefore these only count for one disease if patients are coded with more than one disease within these categories.

The research team will maintain awareness over developments concerning updates to QOF clinical indicators and any changes to the QOF list will be reflected in the inclusion criteria for the study.

The 2015/16 QOF rules have removed osteoporosis and Chronic kidney disease from the disease list. However, given that the management of CKD is similar to the management of hypertension, it is clinically appropriate to still include CKD as an inclusion criteria, but to include this as counting towards one disease condition of 'CVD or CKD' as a single entity.

5.4.2 Exclusion Criteria

In order to maximise generalizability of this intervention, the exclusion criteria have been kept to a minimum. These patients are often excluded from other research and it is these patients with the greatest burden of illness that have the most to gain from this intervention. Therefore the very elderly, patients in nursing homes or housebound, those with mental health problems and those with multiple comorbidities, are all included.

In **England only**, where patients lack capacity, we will seek consent from their legal guardians or consultees. The Scotland A REC board ruled that under Scottish Law, adults who lack capacity to consent should not be included in research studies if not essential. Therefore for practices in **Scotland only**, adults lacking capacity to consent will be excluded. If participants are unable to complete questionnaires themselves, carers can do so on their behalf. We will undertake sensitivity analysis of the impact of including or excluding responses provided by carers on the main results.

Patients who will be excluded:

For peer review only - http://bmjopen.bmj.com/site/about/guidelines.xhtml

- Life expectancy of less than 12 months
- Serious suicidal risk
- Known to be leaving practice within 12 months
- Cannot complete questionnaires in English (themselves or with the help of carers, which may include excluding those with visual or hearing impairment)
- If actively taking part in other research involving extra visits to GP or other health services.
- Adults lacking capacity to consent (Scotland only)

For Peer review only

5.5 Recruitment procedures

5.5.1 Recruitment of General Practices

Local contacts from the Clinical Research Networks (CRN), Primary care and Scottish Primary Care Network will be approached to assist in identifying and accessing GP practices who are interested in taking part in the trial.

Interested practices will be contacted by local researchers who will arrange a meeting(s) with the practice manager, GP partners and practice nurse(s). It is important to get the acceptance and understanding of all the key stakeholders at the practice since the study involves randomisation at the practice level, which therefore requires a commitment to organisational and procedural change. Practice representatives will be asked to sign a practice agreement consenting to the practice taking part in the study.

Since this is a cluster randomised trial, all eligible patients within one practice will be treated in the same way, subject to their consent. Patients will be asked to give consent to being offered care based on the new 3D approach, if their practice is allocated to the intervention arm, and also to complete questionnaires and to allow researchers access to their medical records.

We plan to recruit 32 practices for the main trial: 12 near Glasgow, 10 near Bristol and 10 near Manchester. However we will seek to recruit at least two more practices in each of these areas to act as 'reserves' in case a practice drops out after initially agreeing to take part. In this way we can ensure that practices are blind to which arm of the trial they will be in at the time they are recruited.

5.5.2 Identification and consent of patients

General practices agreeing to participate in the study will be asked to search their practice database using an electronic search strategy devised by the research team based on pre-defined Read-Codes to identify potentially eligible patients who have three or more long term conditions as defined above. GPs will be given the opportunity to screen the resultant list for the above exclusion criteria. The practice will then send out patient invitation packs which will contain an invitation letter from the GP, a patient information sheet explaining about the study, an acceptance/consent form, a baseline questionnaire, and a freepost envelope. Patients will be asked to respond by returning the consent form and completed baseline questionnaire to the research team, using the freepost envelope. Patients who do not wish to participate will be invited to return a blank questionnaire.

Practice staff will also send one postal reminder to individuals who have not responded approximately 10 days after the initial mailing. This may be supplemented with a telephone reminder by a member of practice staff or a researcher with an honorary contract acting on behalf of the practice if necessary.

Maximum statistical efficiency within a cluster trial is achieved if all clusters are of equal size; large variation in the number of patients participating across practices will therefore reduce the power of the trial. For this reason the maximum number of patients within a practice invited to participate will be restricted. This will also reduce the burden on larger practices of potentially having to re-organise care for a large number of patients, and hence limit the cost of the study. The sample size calculation estimated the average number of eligible patients to be invited per practice to be 108 (see section 5.1). We will initially select a maximum of 150 patients in each practice, randomly selecting those to invite from all potentially eligible patients. After GPs have screened and excluded further ineligible patients we anticipate that we will invite up to about 130 patients in larger practices, with the aim of recruiting about 43 patients per practice. These numbers are provisional and may be modified in the light of our experience about the number of potentially eligible patients and the proportion that return questionnaires. Those patients who provide consent to participate in the study form the group of patients who will receive the intervention in the intervention arm practices. If patients wish to talk to someone about the study, they can contact a research contact at their GP practice or a member of the local research team whose details are on the patient information sheet and invitation letter.

This is an individual-cluster trial where the unit of experimentation and the unit of observation is at the level of the individual. Therefore individual level consent is sought to participate in the study, to complete questionnaires and to allow the research team to collect relevant information from their medical notes.

5.5.3 Permission of Patient's Carers

We are also interested in the views and experiences of carers. Therefore as part of the invitation pack to patients, we will also include a form for carers requesting contact details, permission to contact them and consent to complete a separate carers questionnaire at baseline, 6 months and 12 months.

If the GP assesses that a patient lacks capacity to consent, in England, we will obtain the assent of the patient's carer, legal guardian or consultee on behalf of the patient for them to take part in the study.

5.5.4 Baseline data collection

Participants will be sent a postal baseline questionnaire as part of the invitation mailing. The questionnaire will include questions on socio-demographic factors (age, gender, ethnicity, marital status, work status and deprivation status (via postcode) and details of health conditions) as well as baseline measures for all study outcomes. Personal ID and contact details will also be elicited so that the research team can acknowledge receipt of completed questionnaires and provide a £5 gift token in appreciation of the patient's help.

On receipt of a completed questionnaire, the researcher will mail a letter confirming receipt and a £5 gift token in appreciation of the patient's help. This will be followed this up 1 week later with a

For peer review only - http://bmjopen.bmj.com/site/about/guidelines.xhtml

telephone call from the local research team to confirm receipt of the letter and voucher and to provide an opportunity for any questions from the participant.

5.6 Randomisation

GP practices will be the unit of allocation. Practices will be randomised using an automated web randomisation system run from the Bristol Randomised Trials Collaboration (BRTC, UKCRC registration ID: 2) on a 1:1 ratio to receive either the intervention or continue care as usual (control group). Randomisation will be stratified by area (Bristol, Manchester, Glasgow) and minimised by deprivation level and practice size.

In order to minimise post-randomisation selection bias, practices will not be randomised until after patients have been identified and after the initial patient invitations have been mailed.

5.6.1 Control group

Practices allocated to the control arm will continue care as usual. In most practices this will mean patients are recalled to different clinics to see different practice nurses to review each of their long term conditions. The nurses will usually follow disease specific computerised protocols for their management, and will mainly focus on collecting data related to QOF targets rather than quality of life or patients' priorities.

5.6.2 Blinding

Is it not possible to mask participants or health care professionals to the group allocation of their practice. It is also not feasible to blind all members of the study team actively involved in the execution of the study. However, as far as possible, analysis of outcomes will be performed blind.

5.7 Procedure optimisation and pilot

Prior to commencing the main trial in 32 practices we will recruit 3 practices (2 in Bristol and 1 in Manchester) and will use this to optimise the intervention and pilot procedures for the trial. We will implement the intervention as described in all 3 practices. Through direct observation of the training events, observation of some 3D patient reviews and interviews with a sample of patients, GPs, practice nurses, receptionists and practice managers we will seek to improve the training programme, learn from stakeholders about how the intervention can be improved, refine the intervention to improve the likelihood of it being implemented as planned in the main trial, and ensure that the IT infrastructure (computerised search routines and new 3D data collection template) works smoothly in a range of practices. This phase will also enable us to pilot aspects of the trial, including checking that our estimates about the recruitment rate are reasonable, test our patient documentation, pilot data collection mechanisms, and assess follow-up rates. Although the main trial will have started before the patients in pilot practices complete the 6 month follow-up measures, there will be an opportunity to improve follow-up procedures for the main trial because of the time lag between pilot and main trial practices.

5.8 Follow-up procedures

Follow-up data will be collected at 6 and 12 months post randomisation with the primary outcome time-point being at 12 months. The primary method of self-reported data collection will be via postal questionnaires, however alternative completion methods including online, phone or via a home visit will be offered in order to maximise response rates.

For peer review only - http://bmjopen.bmj.com/site/about/guidelines.xhtml

Prior to being sent follow-up questionnaires, the research team will attempt to telephone or email participants to pre-notify them. This is particularly important for those participants reliant on carers/guardians or representatives, if they lack capacity or require help completing the questionnaires. It provides the opportunity for participants to ask questions, remind participants that they are taking part in research (e.g. if they are in a control practice group) and pre-notification has been shown to improve response rates [51].

Two reminders, the first by letter or e-mail (approximately 10-14 days after posting the questionnaire) and the second by phone (approximately 10-14 days after the first reminder), will be conducted for participants who have not returned their questionnaire.

Patients will be given £5 gift vouchers for completion of questionnaires. However, the research team would like the option of performing a sub-study examining the timing of providing vouchers pre- and post questionnaire completion. We envisage submitting this substudy as a substantial amendment to the protocol for REC approval at a later date.

5.9 Withdrawal

5.9.1 Practice Withdrawal

A practice can decline to take part at any time during the initiation and set-up phase of practice recruitment.

Once a practice has been randomised, they may withdraw from the study only under extreme circumstances. If a practice wishes to withdraw the situation should be discussed with the local team, TMG members and CI. All avenues should be explored to try to resolve the problems and concerns of the practice.

If a practice must be withdrawn, then time permitting an additional site will be allocated to the same randomisation group from the 'reserve' practices originally recruited. The new site will be from the same area (Bristol, Manchester, Glasgow) and if possible the same deprivation level (high/low) and practice size (small/large). If there is more than one feasible practice, the replacement practice will be randomly selected.

If a practice must be withdrawn after having signed the study agreement and after their patients have given consent to participate, we will still seek to obtain outcome data on the practice's patients if at all possible. It is likely that withdrawal will be due to practice difficulties in delivering the intervention rather than an unwillingness to allow us to collect data from their patients' records.

Any practice withdrawing, which is replaced, will not be included in the primary intention to treat analysis, but will be included in sensitivity analyses if outcome data are available.

5.9.2 Participant withdrawal

If the participant has already consented, but later decides to drop out, they have several options:

a) Withdrawing from just the data collection (i.e. completing questionnaires and/or allowing researchers to access their medical records), whilst still continuing to receive the intervention if their practice is in the intervention arm.

For peer review only - http://bmjopen.bmj.com/site/about/guidelines.xhtml

b) Withdrawing from receiving the intervention from their practice, if their practice is in the intervention arm, but still completing the data collection

c) Withdrawing from both data collection and intervention if their practice is in the intervention arm.

Any data collected from the patient prior to withdrawal will still be included in the final analysis of the data. Withdrawal from the study does not affect the patients' treatment or access to NHS services.

A notification of withdrawal can be directly from the patient, their carer, GP, or other practice staff. On receipt of such notification, a member of the research team will contact the patients to confirm the request and explain the withdrawal options.

Patients do not have to give a reason for withdrawal if they choose not to although possible reasons could include being too busy, or does not wish to complete any more questionnaires. If the withdrawal is due to the participant experiencing an adverse event, a worsening of their symptoms or has died, the researcher will follow the standard operating procedures (SOP) for adverse events in addition to the withdrawal SOP.

This information will be passed onto the participants' GP in order for their records to be updated.

6 Intervention

This is a complex intervention, with four main components:

1) Identification and prioritisation of patients with multimorbidity: These patients will be identified on practice database systems and 'flagged' in order to prioritise them for a different model of care. These patients will also be provided with a '3D' card in order to identify them with practice staff (see below).

2) Improving patient-centred care: Patients will be prioritised for enhanced continuity of care. Each patient will be allocated a named usual GP and usual practice nurse, who will be identified in the patient's notes and between them will have responsibility for co-ordinating their care. Patients will identify their 3D status to receptionists when booking appointments and will be offered longer appointments with their usual doctor or nurse whenever possible. Other elements of the review of their long term conditions described below (for example the emphasis on care planning) will also enhance patient centred care.

3) Reducing the burden of illness and treatment: '3D' patients will be offered a comprehensive assessment every 6 months, instead of separate reviews for each of their LTCs. Each 3D assessment consists of two appointments a week apart, the first with the patient's usual nurse and the second with their usual doctor. Both will follow the same '3D' assessment structure, supported by a bespoke computerised template:

DIMENSIONS of health. First elicit patients' concerns and priorities for improving their quality of life (e.g.mobility, pain) before collecting data about disease based indicators e.g. weight, BP.

DEPRESSION. The template will include questions to identify depression (PHQ9).

For peer review only - http://bmjopen.bmj.com/site/about/guidelines.xhtml

DRUGS. Before the baseline assessment, a pharmacist will review the patient's medication list to try to identify ways to simplify the patient's drug regime, potentially unsafe drug combinations, drugs which are indicated according to guidelines but are not being prescribed, and drugs which may be indicated but are potentially inappropriate or are low priority for this patient in view of their comorbidities. They will be supported in this by computerised 'rules' applied to the patient's electronic medical record. A note of the pharmacist's recommendations will appear in the 3D review template, for consideration by the GP. This pharmacist review will occur once a year i.e. at alternate 3D reviews. In addition, the template will prompt the nurse and GP to check at every 3D review whether patients are taking the drugs they have been prescribed, using questions designed to detect problems with adherence, and whether they understand what the drugs are for.

At the first 30 minute appointment, the practice nurse will collect information to complete the template and organise all relevant blood tests/investigations. Following the nurse appointment the patient will be given a letter summarising their assessment which details their top priorities for change. This will set the agenda for the second appointment, approximately one week later, with the patient's usual GP. At this 20 minute appointment the GP will review all the information collected by the nurse and from the test results, undertake a through medication review with the help of the pharmacist's recommendations, use evidence based strategies to address the patient's priorities and problems identified in the assessment, and agree a written care plan for the patient to take away. For example, if depression was identified, treatment will be based on stepped care to antidepressants and/or psychological intervention according to severity [52]. An important focus of the GP appointment will be evidence-based approaches to simplifying medication regimes, stopping high risk or low priority drugs and promoting medication adherence [53,54]. Doctors will be trained in these strategies and will be guided by the pharmacist's recommendations [55].

Following the 3D review, follow-up will be determined by the patient's needs and plan.

4) Improving integration. Each practice will have a designated 'general physician' whom they can contact to discuss individuals with complex problems,[56] and to help co-ordinate use of hospital investigations and appointments where patients are attending numerous different specialist clinics or having multiple hospital based tests on different days.

Supporting practices to provide the intervention

Working with commissioners, we will use financial incentives to encourage clinicians to attend training and complete 3D assessments, as if the intervention was included in a Local Enhanced Scheme[57] or part of the QOF.

We will provide training to help practices to implement the intervention as intended. All GPs and all practice nurses involved in LTC management will be asked to attend two half days of training, with practices training together to share ideas. Practice nurses and GPs will be trained to use the 3D assessment template, to focus on addressing issues relating to quality of life, depression, and medication adherence as well as disease management, and how to create a written care plan with the patient. A substantial element of the training will be devoted to promoting attitudinal change amongst clinicians towards identifying and responding to patients' own priorities and problems with broader quality of life, as organisational change is unlikely to be effective unless clinicians 'buy into' the underlying philosophy of the new approach [50].

Following initial training, we will appoint a local GP champion in each practice to monitor and maintain adherence with the intervention amongst practice staff and to promote the research. GP

For peer review only - http://bmjopen.bmj.com/site/about/guidelines.xhtml

champions from intervention practices in each area will meet together in local collaboratives to share good ideas and experiences. We will allow local adaptation of the intervention to reflect local context while ensuring the key elements of the conceptual framework are maintained.[58] We will provide regular feedback about implementation e.g. the number of patients who have had a 3D assessment (which we will monitor through regular searches in the intervention practices, with the help of practice champions).

GP Receptionists will be offered training at their own practice. Receptionists will have a key role in ensuring that patients are offered longer appointments with their usual nurse or GP. They will be trained in strategies and forms of words to achieve this.

7 Data Collection

Participant study data will primarily comprise of self-reported questionnaires and GP patient records. Research teams at each site will be responsible for data collection including sending the questionnaires, collating, logging the data and entering the data onto the trial database and collecting data from GP records. Letter and email reminders will be sent to participants who do not return questionnaires within 14 days. Participants may receive a further phone reminder or may be called if the primary outcome is incomplete.

7.1 Quantitative Outcome Measures

All outcomes are at the level of the individual patient and will be collected at baseline, 6 months and 12 months post randomisation.

7.1.1 Primary Outcome

The primary outcome is health related quality of life, measured using the EQ-5D-5L [59] at 12 months. This is a self-report measure which will be collected by paper questionnaires.

7.1.2 Secondary Outcomes

These patient reported outcome measures will be collected at baseline and 6 and 12 months post randomisation from patient questionnaires.

Burden of illness measures to assess quality of life, disease control and depression:

- Self-rated health (single item)
- Bayliss measure of illness burden in multimorbidity [60]
- Quality of disease management (composite measure of QOF achievement) [61]
- Hospital Anxiety Depression Scale (HADS) [62]

Burden of Treatment measures to assess the problems of poor coordination of care, multiple appointments and polypharmacy:

- Brief Treatment Burden Questionnaire
- Morisky Medication Adherence Scale (8 item) [64]
- Number of drugs prescribed

For peer review only - http://bmjopen.bmj.com/site/about/guidelines.xhtml

- Reduction in high risk drug combinations[65]

Experience of patient centred care, to assess the lack of holistic patient centred care:

- CARE measure and how well you know your doctor[66]
- Coordination of care and care related to patient's priorities (questions from LTC6 QIPP)
- Management of LTCs (PACIC) [67]
- Overall satisfaction (single item)

7.1.3 Socio-demographic measures

The following socio-demographic measures will be collected from self-reported paper questionnaires at the baseline timepoint only (prior to GP practice randomisation). These measures will allow comparison of population composition between study groups and study centres, and will also be used for subgroup analyses.

- Number of long term conditions
- Age (on date of invitation to participate)
- Gender
- Education
- Ethnicity
- Deprivation status – based on postcode which maps onto the index of multiple deprivation.
- Work status

7.1.4 Carer outcomes

The role of the carer in supporting a patient with multimorbidity is often neglected. In order to assess whether the change in GP management affects other aspects of the participant's care we request that the participant's carer also complete questionnaires about their experience and how it affects their wellbeing. Carer contact details and consent to complete brief surveys will be requested in a form which will be included as part of the initial patient invitation mailing pack. Carers will be requested to complete the survey at baseline, 6 and 12 months follow up.

Measures of strain on patient's carers:

- Carer Experience Scale [68]
- EQ-5D-5L [59] completed by carers
- Brief Treatment Burden Questionnaire for carers

7.1.5 Resource use

For peer review only - http://bmjopen.bmj.com/site/about/guidelines.xhtml

Resource use will be compiled from a combination of self-report measures in participants' follow up questionnaires at 6 and 12 months and electronic GP records examined at the end of the study. Participants will be asked to provide information concerning:

- Hospital admission and outpatient attendance.
- Use of health services in primary, community and secondary care

To simplify data collection, the questionnaires will ask whether participants have been admitted to hospital. If so, the researcher may phone the participant to obtain more detailed information about reason, length and place of stay if the information is not available in their GP notes.

GP records will confirm participants' resource use over the course of the 12 months that they were in the study. Researchers will request access to patients' records after 12 month follow-up data collection has been completed. In addition to the above data, the following information will be collected from GP records:

- Any health investigations the participant underwent
- All medications prescribed for the patient over the course of the study

Resource use information is required for the economic evaluation of the service. It is also a way of demonstrating changes in the process of care.

7.2 Data Management

7.2.1 Source data

Data will primarily be in the form of participant self-report paper questionnaires, logs collated from GP/nurse 3D computer template usage, extractions from patient records and case report forms. Research teams at each recruitment site will be largely responsible for the collection and monitoring of data from participants and practices within their areas. Overall responsibility of the study data will be with the Chief Investigator which can be delegated to the Trial Manager and Data Manager.

Patient, carer and practice staff contact details needed for day-to-day trial management will be held on a bespoke management database, designed and managed by the BRCT and held on secure servers at the University of Bristol. Since ID numbers will be accessible from this system, researchers will have unique logins and restricted access to the appropriate recruiting centre data only.

All paper questionnaires and records will be entered into a separate tailor-made database held on a central university server. The data is entered via a securely authenticated, web-based, commercial software system called REDCap. This database will only store anonymised questionnaire and CRF data. Study researchers will be given a unique login in order to access and input on this database. At least 10% of questionnaire data from each site will be subject to double entry and second checking to ensure quality and inter-rater reliability.

Qualitative data collection will be conducted and analysed by the qualitative researcher (CM) and will involve a variety of mixed media. Full details are provided in the Process Evaluation section of this document (section 7.6)

For peer review only - http://bmjopen.bmj.com/site/about/guidelines.xhtml

In accordance with REC requirements, regulatory authorities including monitors and auditors from NHS Trusts may request access to source data and documents for cross checking. This will be explained in the participant information sheet and a statement included as part of the written consent form to be signed by the participant.

7.2.2 Data Storage

Where possible, personal identifiable details will be removed from hard-copy documents and replaced with the participant's unique trial identification number. During the study, all hard copy documents containing patient identifiable data (e.g. consent forms) will be stored in (as a minimum) locked filing cabinets within alarmed, access restricted University buildings of each of the research centres. Only local research teams will have access to these locked cabinets.

Electronic data will only be accessible via a password protected database held on a secure server.

See section 11.3 for long term storage procedures at the end of the study.

8 Data Analysis of quantitative trial outcomes

8.1 Sample Size calculation

The study is designed to detect an effect size of 0.274 standard deviations in the primary outcome of the EQ5D-5L. Data about the variability of the new 5 level (5L) version of the EQ5D is currently more limited than for the well-established 3 level (3L) version. The standard deviation of the EQ5D-3L in the UK general population is 0.23, rising to 0.27 in the oldest respondents (aged over 75).[69]Hence an effect size of 0.274 would equate to a detectable difference of (0.274*0.27) = 0.074 on the EQ5D-3L, previously deemed to be the minimum important difference.[70] Although there are less data about the variability in the 5L version of the EQ5D than the 3L version, it seems wise to use this latest version of the EQ5D as it is likely to have greater sensitivity to change.

Based on data available from our previous studies, we estimate that 2.3% of adult patients will have multimorbidity in terms of 3 or more LTCs as defined in this study. This equates to about 108 patients in an average sized practice of 6000 patients i.e. 3456 potentially eligible patients in 32 practices. Assuming 40% of patients agree to participate (n=1382), 80% are followed up to 12 months, and an ICC of 0.03 for clustering at the practice level (based on the WISE trial)[31] this sample provides around 90% power at a 5% significance level to detect an effect size of 0.274 standard deviations in the EQ5-D measure between the intervention and control groups. This is considered a small effect size.

In practices with large numbers of potentially eligible patients we will randomly select a maximum of 150 potential participants, as described in section 2.5.2. After GPs have excluded ineligible patients we anticipate inviting about 110 patients per practice, on average. The number of patients initially selected will be reviewed following the optimisation phase

Efficacy data analysis

Data will be analysed in accordance with CONSORT principles and its extension for cluster randomised trials. Descriptive statistics will be used to summarise characteristics of both practice and patients and compare the balance across groups. We will take account of clustering by practice

For peer review only - http://bmjopen.bmj.com/site/about/guidelines.xhtml

in all analyses using multi-level regression models. A full statistical analysis plan will be developed and agreed by the Data Monitoring Committee (DMC) and Trial Steering Committee (TSC) after the pilot phase and prior to undertaking any analyses of the main trial.

8.1.1 Primary analysis

Intention to treat analysis will compare groups using a linear multi-level regression model adjusted for baseline values, stratification/minimisation variables, clustering and other important co-variates such as age, number of long-term conditions, deprivation, depression. The results will be presented as the difference between group means and corresponding 95% confidence intervals will be derived.

8.1.2 Secondary and subgroup analysis

Multi-level regression models will be applied to evaluate differences between groups for the pre-planned analyses of secondary variables. These will be based on logistic or linear regression as appropriate.

We will use sensitivity analysis to examine the effect of missing data on outcomes based on different assumptions. We will conduct subgroup analyses using appropriate interactions terms to explore the effectiveness of the intervention in relation to age, number of long term conditions, index of deprivation and presence or absence of depression alongside physical health problems. These subgroup analyses will be hypothesis generating and will focus on interpretation of 95% confidence intervals rather than P-values. Subgroup analyses are likely to be insufficiently powered since the trial was not powered to specifically test these effects.

8.1.3 Use of anonymised data

Anonymised data will be used for the following:

i) Comparison of descriptive data for consenting vs. non-consenting patients

ii) Comparison of QOF performance in patient with LTCs with and without multimorbidity. This is to assess for any unintended consequences, ie. whether concentrating effort on patients with multimorbidity has any positive or negative impact on the care of other patients.

iii) *iii)* Comparison of hospital admissions and deaths. We will explore the feasibility of obtaining data via anonymised records linkage (HES data and death registry) in order to examine the impact of the intervention on all eligible patients in participating practices, and also to compare the impact on patients with or without multimorbidity, without needing individual patient consent or access to identifiable patient data. Without these data, we will only be able to collect this information for patients who return questionnaires, and this analysis would lack power since the events (particularly deaths) are relatively rare.

9 Economic Evaluation

Economic analysis will be undertaken from the perspectives of:

a) NHS and personal social services (PSS)

For peer review only - http://bmjopen.bmj.com/site/about/guidelines.xhtml

b) Patients

We will also measure and value the time off work and usual activities.

Resource use data will be collected from self-reported patient questionnaires from questions of utilisation of primary, community and secondary care services and personal costs (including travel, loss of earnings and dependent care costs). These data will be supplemented by GP records and trial records where appropriate. Unit costs will be valued using national published sources such as Curtis [71] , NHS reference costs and the British National Formulary (BNF).

The set-up and training costs of the intervention will be identified and estimated separately from the running costs. The costs of each component of the intervention will also be estimated separately from each perspective and when related to changes in a range of outcomes will give the cost consequences.

Cost effectiveness analysis from the NHS and PSS perspective will estimate the incremental cost per Quality Adjusted Life Year (QALY) gain. A QALY is a composite measure of health combining quantity and quality of life. QALYs will be estimated using the EQ-5D-5L.

Uncertainty will be addressed in sensitivity analyses and by using bootstrapping to estimate the net monetary benefit and a cost-effectiveness acceptability curve.

10 Process Evaluation

10.1 Background

Process evaluations of complex intervention trials are recommended by the MRC Framework [39] and have many potential purposes [72], but there is relatively little guidance on how best to design them. One of the applicants has recently published a framework for process evaluation design which emphasises the importance of explicitly stating the aims of the evaluation and the choice of processes to examine [72]. The aims of the mixed methods process evaluation in this project are:

- If the intervention is effective, to identify and document examples of good practice and strategies for successful implementation, and also practical difficulties in implementation and maintenance, in order to inform commissioning and other long-term implementation.

- If the intervention is ineffective, to identify if this is due to problems with the intervention (failure of intervention concept despite the intervention being delivered as intended) or problems with intervention delivery (implementation failure, which may occur at multiple points) [73].

The process evaluation will therefore explore issues of representativeness, intervention implementation, reach at patient level, nature and fidelity of intervention delivery, maintenance of the intervention over time, and patient perceptions of how care has changed. This will help to gain understanding about why the intervention worked (or did not work), and will inform and facilitate commissioning and other implementation of the intervention if appropriate.

The process evaluation will also include practices in the usual care group, in order to clarify what are the current practices in the management of multimorbidity patients. Given the recent changes in the NHS and GP contracts and structures, it is necessary to characterise what usual care is at the start of the trial and whether this changes over the intervention period so that we define what we are

For peer review only - http://bmjopen.bmj.com/site/about/guidelines.xhtml

comparing the intervention to. This can also help to inform of ways of future delivery of the intervention.

10.2 Design

The process evaluation will be mixed methods, combining analysis of quantitative process data (such as patient characteristics, number of consultations, number of drugs prescribed) from all practices with an in-depth, comparative case study [74] in up to five purposively selected intervention practices, chosen to represent those in different settings and with different prior levels of organisation of care for patients with LTCs. Data will be supplemented by interviews with commissioners.

There will be an optimisation study preceding the process evaluation of the main trial. The aim of the optimisation study is to help the main trial team optimise the intervention. This will be achieved by evaluating implementation of the different elements of the intervention to identify any implementation problems or divergence from the intentions of the trial. It will also allow the process evaluation methods to be used in the main trial to be piloted and evaluated. The methods will be as described below for the main trial but in smaller numbers i.e. 2 case study practices and some supplementary interviews and observations in the other 2 pilot practices

10.3 Methods and Setting

Up to five purposively selected case study practices will be chosen, to represent different settings and with different prior levels of organisation of care for patients with LTCs. Additional data regarding some processes may be collected from other practices participating in the 3D trial depending on fidelity and implementation issues identified during the course of the process evaluation. Information regarding usual care will be collected from all practices at the start of the trial and at the end of the intervention period, by questionnaire supplemented by interview if necessary.

Quantitative methods will involve the analysis of routinely available data on practice characteristics and data collected as part of the trial.

Qualitative methods will involve semi-structured interviews, focus groups and direct observation of these case study practices. If necessary, qualitative data will be collected from some other practices taking part in the 3D trial in response to particular issues that arise during the trial. These methods are described in detail below.

10.4 Sampling

Practices: Up to 5 study practices will be purposively selected to take into account geographical region, socioeconomic deprivation levels and different prior levels of organisation of care for patients with LTCs. Practices will be the main unit of sampling and within these case practices, we will sample a range of people (patients and staff) and events (consultations, appointment bookings) over a period of 12 months.

Practice staff: We will undertake approximately 40 interviews sampled purposively to reflect a range of relevant professionals including receptionists, practice nurses, GPs and Practice Managers. These will mainly be in the case study practices but may also include some interviewees from other practices taking part in the 3D trial.

For peer review only - http://bmjopen.bmj.com/site/about/guidelines.xhtml

Patients: Approximately 6 patients per case study practice and some from other practices participating in the trial (n= 30 total approx.), will be purposively sampled for heterogeneity in age, sex, socio-economic status and LTCs.

10.5 Recruitment

Staff: We will write to practice staff in case study practices and some other participating practices asking for consent to participate in an interview, and in some cases, to participate in recording and observation of a consultation with a patient. We will include an information sheet and consent form.

Patients: When consenting to participate in the trial, participants will be asked if they are willing to be approached by a member of the research team at a later stage to consider taking part in an individual or focus group interview and in the observation and recording of a consultation. If they agree, we will write to them with an information sheet and relevant consent form if they are sampled for interview.

10.6 Data Collection for Process evaluation

10.6.1 Quantitative data

Quantitative data on practice recruitment and representativeness, reach at patient level (whether or not patients receive the intervention), implementation of intervention elements and whether or not the actual process of care is different from usual care from the patient's perspective will be gathered as part of the main trial data collection (Box 1). Data on recruitment will be used to examine representativeness of participating practices to inform judgements about generalisability.

Box 1 Process evaluation quantitative questions and methods

PROCESS: RESEARCH QUESTION	DATA COLLECTION
**Recruitment:** How representative are recruited practices?	Analysis of routinely available quantitative data on practice characteristics (comparing characteristics of recruited and non-recruited practices)
**Reach at patient level:** What proportion of targeted patients receive the intended assessment?	Analysis of quantitative data collected as part of trial (for practices in the intervention arm):<br><br>Number of patients given a 3D card, receiving a comprehensive 3D assessment and components of this (e.g. quality of life discussion, mental health assessment, care plan, treatment for depression).
**Evidence of intended changes in care.** Is the process of care in intervention practices different from that in control practices?	Analysis of quantitative data collected as part of the trial (comparing practices in the intervention and control arms) e.g. number of appointments per patient; Continuity of care (COC measure of longitudinal continuity [75]); Number of contacts with hospital general physician; provision of pharmacy advice, number of drugs prescribed.

10.6.2 Qualitative data

We will collect qualitative data in the following ways see Box 2.

For peer review only - http://bmjopen.bmj.com/site/about/guidelines.xhtml

1. Observation/recording of training sessions for intervention practices
2. Interviews and focus groups involving patients/carers
3. Observation of practice setting and reception
4. Observation/recording of 3D consultations
5. Interviews with practice staff

Semi structured interviews of lead GPs and practice managers will occur at two time-points (approximate total n=20 interviews).

i) Early in the implementation, approximately 4 weeks after initial training to reveal whether and how practices plan to reorganise care to deliver the different elements of the intervention.

ii) Towards the end of the trial in order to elucidate whether and how practices maintain the reach and fidelity of the intervention over time. In addition what where the barriers and facilitators of successful maintenance?

Additional practice staff including receptionist, practice nurses and GPs (will also undergo semi structured interviews at 2 time points (approximate total n = 20).

i) Soon after initial training to appraise care at baseline and to evaluate the adequacy of the training and attitudes towards the change in care processes

ii) After 3D reviews have been begun to appraise how the intervention was delivered in the practice and how it affected their individual roles. For nurses and GPs, also to evaluate a recently recorded 3D review

Approximately 6 patients per case study practice and some from other participating practices (approximate total n = 30) will be invited to one focus group per practice before the intervention to assess how they perceive existing care. After the intervention has been implemented, they will undergo a semi-structured interview, which may be after a recorded consultation to assess how patients perceive the changes in their care and to evaluate a recently recorded consultation. These interviews will be conducted at a time and place agreed by the participant and can include the patient's home or University building. At the end of the interview, participants will be offered a £5 gift voucher as a goodwill gesture for their time.

Non-participant observation of the training delivered to practices and completion of evaluation forms by participants and trainers will assess the response of practice staff to the intervention and the adequacy of the training.

Non-participant observation of appointment booking at the reception desk and by telephone will examine if and how the appointment system has been reorganised for participating patients.

A sample of 3D assessment appointments in case study practices and some in other intervention practices (approximate total n=20) will be audio or video recorded and/or observed to evaluate the nature and fidelity of this aspect of intervention delivery.

For peer review only - http://bmjopen.bmj.com/site/about/guidelines.xhtml

Box 2 Process evaluation qualitative questions and methods	
**PROCESS: RESEARCH QUESTION**	**DATA COLLECTION**
**Usual care:** How does organisation of care for patients with multi-morbidity vary across practices and over the timeframe of the trial?	Descriptive data about organisation of care and appointment arrangements collected from informal interviews at time of recruitment and by contact with practice manager later in trial
**Initial response of practices to training:** What is the response of practice staff to the intervention training? Whether and how do practices plan to or initially reorganise their care to deliver the different elements of the intervention?	Participant and trainer evaluation of all training sessions. Non-participant observation of some training sessions. Semi-structured interview with lead GP and practice manager in up to 5 case study practices and some other participating practices, in the 4 weeks after the initial training.
**Nature and fidelity of the intervention:** How is the appointment system reorganised (or not) to deliver continuity? What is the actual content of 3D assessments, and does it match what was intended by the research team? What aspects of the intervention seem to be most (and least) effective, and how does this vary with patient circumstances and practice context? How could the intervention be improved?	Non-participant observation of appointment booking at the reception desk and by telephone in case study practices.<br><br>Audio or video recordings and/or observation of a sample of 3D assessment appointments in case study practices and some in other participating practices (approximate total n=20).<br><br>Semi-structured interviews at 1 or 2 time points with practice manager, receptionist, practice nurses and GPs (approx. 8 interviews per case study practice, plus some in other participating practices approx. 40 total).<br><br>Review of log of contacts with hospital physician supplemented by brief interview if indicated
**Maintenance:** Whether and how do practices maintain the reach and fidelity of the intervention over time? What are the barriers to, and facilitators of successful maintenance?	Semi-structured interview with lead GP and practice manager towards the end of trial. Interview with practice champions. (Number included in interviews above)
**Patient perspectives:** How do patients perceive the changes to their care and what is their experience of the 3D consultations?	Semi-structured interviews and focus groups with up to 6 patients per case study practice and some other practices participating in the trial (n= 30 total approx.), purposively sampled for heterogeneity in age, sex, socio-economic status and LTCs. Interviews will in some cases follow observed consultations in order to evaluate the consultation.

10.7 Commissioners

We recognise that implementation of findings from health services research can be problematic, but will seek to facilitate implementation by designing our intervention to be highly pragmatic and potentially commissionable. We will therefore additionally interview a sample of 6 commissioners (2

For peer review only - http://bmjopen.bmj.com/site/about/guidelines.xhtml

from each study area) both before and after the intervention is introduced. Before, we will interview them about ways to ensure that the intervention is introduced in a way which lends itself to commissioning, to identify barriers and facilitators to commissioning this type of service, how the new approach would fit with current commissioning models which are likely to be mainly in disease-specific service silos, and the advantages and disadvantages of different approaches to incentives (e.g. QOF-type targets, Local Enhanced Services etc.). Towards the end of the study we will interview the commissioners again, showing them the findings from our research, in order to discuss with them the most important messages, potential barriers to implementation and how to mitigate them, current commissioning priorities and policy changes and implementation tools to help to ensure that (if effective) the intervention is commissioned in future. This may be supplemented by interviews with additional commissioners at later workshop/dissemination events.

10.8 Data Analysis

Data analysis for the process evaluation will initially focus on the individual datasets. Quantitative recruitment/ representativeness from all practices will be analysed using chi-squared tests for categorical variables and t-tests or non-parametric tests for continuous variables, and will inform judgements about the generalisability of the findings. Failure to deliver the intervention to target patients is an important potential cause of lack of effectiveness for any intervention, and will be examined by determining what proportion of the target population actually received any or all of the intervention components, and how this varies between practices. Differences between intervention and control practices in terms of process of care will be analysed using the same regression analysis as for main trial outcomes (described above). These data (for example about number of appointments, continuity of care, patient centred care) will also help to characterise 'usual care' in the control arm practices.

For the optimisation phase, the focus of the qualitative data collection and analysis will be on practices' response to the intervention, any issues they have with implementation, and fidelity of their intervention delivery to patients. Analysis will employ the 'framework' method [76] in order to quickly feed into improvements to the intervention and analysis that may be required for the main trial.

For the qualitative data from the case study practices and other participating practices, analysis will take place alongside data collection to allow early analytic insights to inform ongoing data collection. Field notes from observation of training, appointment bookings and consultations will be coded alongside transcripts from recorded 3D consultations, interviews and focus groups, and used to characterise organisation and delivery of care in different contexts, as well as responses to, and implementation to the intervention. Emerging themes and theoretical ideas will be discussed and refined at team meetings throughout the research. The qualitative data from multiple sources, alongside the quantitative data, will allow us to build up a 'thick description'[77] of each case study site, helping us to understand whether, how and why the intervention worked in each practice. Analysis of these data will enable us to elucidate the internal processes and interactions (e.g. staff-staff, staff-patient) that impact the delivery, implementation and maintenance of the intervention. Across the cases, analysis of both qualitative and quantitative data will allow us to identify factors which are plausibly and/or consistently related to successful or unsuccessful delivery of the components of the intervention and changes to the experience of care by patients.[73,74] As analysis progresses, the fit of the data with models of patient centre care[40] will be evaluated, for example, giving attention to whether and how the intervention enhances the therapeutic alliance between patient and doctor. The final stage of analysis will be to draw together the findings from

For peer review only - http://bmjopen.bmj.com/site/about/guidelines.xhtml

the broader quantitative analysis and the in-depth case studies to create an understanding of why the intervention did (or did not) work, why this happened, and identify implications for longer-term implementation if appropriate.

10.9 Unintended consequences

There is a theoretical concern that focusing effort on one group patients (in this case multimorbidity) could lead to reduced efforts and reduced quality of care in the other patients.[14] In order to compare performance in terms of the QOF in patients with and without multimorbidity, we will collect anonymous data about the performance against QOF targets for all patients in participating practices with *any* of the index conditions (individually or in combination) that are included in our definition of multimorbidity, using electronic download from medical records. We will compare QOF performance in patients with and without multimorbidity in the year before and the year after the intervention, to check whether concentrating effort on patients with multimorbidity has any positive or negative impact on the care of other patients.

Similarly, we will explore whether it is feasible to obtain anonymised data about deaths and hospital admissions for all patients in participating practices, as this would enable us to compare these outcomes in patients with and without multimorbidity in intervention and control practices (see section 5.1.3)

11 Research Governance

The trial will be conducted to protect the human rights and dignity of the participant as reflected in the 1996 version of the Helsinki Declaration. Patients will not receive any financial inducement to participate. In order to protect the trial participants the following provisions will be made/upheld; the trial has been designed to minimise pain, discomfort and fear and any foreseeable risk in relation to the treatments involved; the explicit wishes of the participant will be respected including the right to withdraw from the trial at any time; the interest of the patient will prevail over those of science and society; provision will be made for indemnity by the investigator and sponsor.

The trial will be registered with a publicly accessible trial register (Trial registration number: ISRCTN06180958)

This study will be conducted in accordance with principles of good clinical practice (GCP) and a favourable ethical opinion from the appropriate Research Ethics Committee and local NHS R&D approvals from the appropriate trusts will be obtained prior to commencement of the study.

11.1 Ethical conduct

We are aware that patients with multimorbidities require quite complex care. However patients will not be denied any form of care that is currently available in the NHS by participating in this study. Patients from usual care practices will still have access to NICE recommended treatments and local provision of services. Whereas patients from intervention practices will still have full access to their GP and secondary care services in addition to their 6 monthly 3D assessments.

This study raises the following ethical issues:

(i) Cluster level randomisation: This is a complex organisational intervention, therefore randomisation needs to be at the level of the organisation, ie the practice. If one tried to randomise by individual patients, there would be problem of contamination (GP trained in

For peer review only - http://bmjopen.bmj.com/site/about/guidelines.xhtml

the new way of working would change their 'usual' care practices and the study would not be able to test the effect of practice re-organisation).

(ii) The role of individual consent: Cluster randomised trials have the issue that all eligible patients in 'intervention' practices will receive the intervention even though they will not be asked to give individual consent. This is justified because the intervention has few risks, and it is normal for general practices to try out new ways of working with the aim of improving patient care, without needing individual patient consent. However, patients will be asked to give consent to complete questionnaires and to allow researchers to have access to their notes, and only consenting patients will be included in the analysis of the main trial outcomes.

(iii) Use of anonymised data: Anonymised data will be used for some analyses e.g. comparisons of consenting and non-consenting patients.

(iv) Patients lacking capacity to consent: Because we wish to have broad inclusion criteria we will ask carers to help with completion of questionnaires where appropriate. In England, we will seek assent from legal guardians or consultees where potential participants do not have capacity to give consent themselves. In Scotland, adults lacking capacity to consent will be excluded from the study

For peer review only

11.2 Safety Assessment and Adverse Events

The study will monitor the occurrence of any serious adverse event which arises whilst the participant is taking part in the trial.

11.2.1 Definitions

An **adverse event** is any **unexpected** effect of an untoward clinical event affecting the participant. This is classed according to severity i.e.

a) Non-serious adverse event (AE) – which includes discomfort or a slight worsening of symptoms

b) Serious Adverse Events (SAE) – which may be particular harmful, dangerous or require hospitalisations.

An SAE is defined as one of the following:

1. results in death;
2. is life threatening;
3. requires hospitalisation or prolongation of existing hospitalisation;
4. results in persistent or significant disability or incapacity;
5. consists of a congenital anomaly or birth defect;
6. is otherwise considered medically significant by the investigator.

For peer review only - http://bmjopen.bmj.com/site/about/guidelines.xhtml

Given that patients with multimorbidity may be heavy users of secondary care services, new medical diagnoses, hospital admissions and deaths are expected and will not be considered as SAEs unless it appears that they may have been related to the intervention of the research process. Hospitalisations and deaths will be counted as part of the service use data and reported as part of the trial analysis.

11.2.2 Detecting and recording AEs and SAEs

Adverse events may be reported by several methods:

1. Directly by the participant (ie by email, phone call or voice mail message)
2. From the participants follow up questionnaires
3. Indirectly from family members, carers, guardians or representatives
4. From the participants GP practice (following the patient's 6 monthly assessment or information passed on from hospitals).

Participants and GP practice staff will be asked to notify any adverse event which they believe may have occurred **as a result of** the trial intervention or the research process. On notification of an such an adverse event which may be related to the research process or intervention, a researcher should complete an adverse event reporting form within 5 working days, paying specific attention to information regarding the timescale of events i.e. when the event started, were there any specific changes to medication or behaviour preceding the event. Further information should be requested from the participant or GP/practice nurse as necessary.

A completed form should be securely sent to the Trial Manager who will pass it onto the trial clinician to review.

11.2.3 Assessment of relatedness and expectedness

The trial clinician will make the following decisions

1. Whether the adverse event is an AE or SAE
2. How related is the event to the study intervention and/or research processes, according to the following definitions:

Unrelated – where an event is not considered to be related to the study intervention or research processes

Possibly – although a relationship to the study intervention and/or research processes cannot be completely ruled out, the nature of the event, the underlying disease, concomitant medication or temporal relationship make other explanations possible

Probably – the temporal relationship and absence of a more likely explanation suggest the event could be related to the study intervention and/or research processes

Definitely – Known effects of the study intervention and/or research processes, or based on challenge testing, suggest that study intervention is the most likely cause.

For peer review only - http://bmjopen.bmj.com/site/about/guidelines.xhtml

3. Expectedness of the event. Is the event an anticipated medical event even if the research had not been taking place?

4. Is further action required?

11.2.4 Reporting requirements for SAEs

All reporting of SAEs of a related and unexpected nature will follow regulatory reporting requirements as set out in article 17 of the EU directive 2001. These will be reported to the sponsor immediately and will be reported to the REC within 7 days of the Trial Manager becoming aware of the event. Any relevant further information will be subsequently communicated within 8 days. In addition all investigators will be notified.

The TSC will be notified immediately of all SAEs thought to be treatment or research related. Potential SAEs which after review are not thought to be treatment or research related will be brought to the TSC's attention at their next scheduled meeting. The numbers and details of all AEs and SAEs will be reported to the Trial Management Group, Trial Steering Committee and Data Monitoring Committee.

11.3 Monitoring

Research procedures and progress will be constantly monitored by the Trial Management Group.

The research team will establish processes to enable practices to regularly monitor the extent to which they are implementing the intervention as planned. This will include regular audits of the number of patients who have been invited to a 3D assessment, the proportion that have attended both assessments, and the continuity of care provided. In addition, each practice will have a 'GP champion' who will informally monitor progress in order to ensure that the intervention is delivered as intended.

By participating in the trial, patients will receive a more intensive level of monitoring than that normally received in primary care.

All aspects of the study will undergo regular external monitoring by the independent Trial Steering Committee (see section 9.5) and Data Monitoring Committee (section 9.6). It may also be open to audit and monitoring from local NHS R&Ds.

11.4 Patient and Public Involvement

During the development of the proposal, two patient and carer open forum meetings were held whereby several support groups for various LTCs, carers and patients groups from GP practices were invited. Patient and Public Involvement (PPI) coordinators facilitated discussions aimed at asking patients directly what their health issues and concerns were and what services they would like in order to improve management of their conditions. This helped to inform the design of the intervention.

15 people were willing to continue being involved in the research and these will form a patient involvement group. The aim would be to meet approximately 4 times a year to advise on study patient information leaflets, questionnaire design, ethical issues, recruitment approaches, dissemination of results and impact. This group will be facilitated by CM and will be funded to attend PPI training workshops. Reimbursement will be provided in line with INVOLVE guidance.

For peer review only - http://bmjopen.bmj.com/site/about/guidelines.xhtml

Two members of this patient group, along with Prof James Goodwin, Head of research at Age UK will sit on a research advisory group and Trial Steering Committee whose role is to oversee the management of the research. Another member will be invited to contribute to the qualitative data analysis by helping to identify themes and commenting on findings. Therefore service users and patients will be directly involved in the research design, management, and analysis of the study.

We will take advice and use contacts within long term conditions groups to help reporting of results and dissemination of the research findings on their newsletters and websites, which will greatly enhance the relevance and reach of the study.

11.5 Confidentiality

All eligible participants will be allocated a unique trial identification number. This number will be used to identify patients throughout the study. Where possible, personal identifiable data will be removed from all collected data and replaced with the trial id number, thereby providing a level of pseudo-anonymisation. Only researchers needing to make direct contact will have access to the password protected database which links patients' personal details with their identifier.

Clinical information about identifiable patients will not be released without written permission from the participant. The exceptions are a) if the researcher is concerned for the participant's safety or well-being or b) if required for monitoring and auditing by the sponsor, regulatory authorities or by the REC or c) release of information to clinicians who are entitled to be aware of it as part of the patient's direct clinical team e.g. their GP or GP practice staff.

11.6 Data Protection

All collection, storage, processing and disclosure of personal information will be performed in compliance with the Data Protection Act 1998. All investigators and study staff will uphold the Act's core principles. Any communications, reports or published results will not contain any personal data that could allow identification of individual participants.

All computers used to collate data will have limited access measures via user names and passwords. Electronic mobile devices used to collect data will be encrypted. Databases and servers are stored in right-restricted areas with limited access. All data will be stored in locked facilities within secure offices.

12 Trial Management

12.1 Funding

Research funding has been secured from NIHR Health Services and Delivery Research Programme.

12.2 Sponsorship

The University of Bristol will act as sponsor for the trial. The sponsors contact is Dr Birgit Whitman, University of Bristol, Research and Enterprise Development, Senate House, Bristol, BS8 1TH.

For peer review only - http://bmjopen.bmj.com/site/about/guidelines.xhtml

12.3 Insurance and Indemnity

Normal NHS indemnity procedures will apply. The University of Bristol will also provide relevant public liability cover, as detailed in the certificate of insurance.

12.4 Trial Management Group (TMG)

The Trial Management Group will meet regularly (every 6 -8 weeks) in order to ensure that the three study centres are working consistently, meeting study targets and adhering to the study protocol. The group will consist of the CI, Trial Manager, PI and researchers from each of the recruiting centres with input from other members of the research team where necessary. Regular progress regarding study recruitment, retention, issues or complaints and adverse events will be reported and discussed.

12.5 Independent Trial Steering Committee (TSC)

An independent Trial Steering Committee will be convened comprising of an external academic chair (who is also an academic GP), at least two other independent members (including an independent statistician or trial methodologist and an independent clinician with relevant experience or interests), two patient representatives, the CI, PIs and other key members of the study research team. The TSC will meet at least annually (face-to-face or by teleconference) or more frequently at the request of the chair. The TSC will provide external supervision to the study and monitor the overall trial progress, adherence to the protocol and the implications of any new information (e.g. research articles or policy changes).

12.6 Data Monitoring Committee (DMC)

This committee will comprise of an independent chair and at least two other independent members including an independent statistician and a clinician with relevant interests. The CI, Trial Manager and Trial statistician will report to the DMC. The remit of the DMC is to monitor the trial data, in particular to quality control and quality assurance of data collected; the progress of the trial, including recruitment and retention rates and adherence to the trial protocol. A key role is to ensure that the dignity, rights, safety and well-being of the study participants are maintained at all stages of the trial. All adverse events will be reported to the committee which can have direct access to source data and documentation. Where possible the DMC will convene prior to the TSC and will report their recommendations to the TSC.

For peer review only

12.7 Advisory Group

An advisory group will be convened to ensure the research takes account of developments in the NHS. Prof James Goodwin (Head of Research, Age UK), Ms Jane Whittome (Head of Programme for Long Term Conditions for NHS Improving Quality), and Dr Kevin Gruffydd –Jones (RCGP) have agreed to join this advisory group, which will also include the chair and lay members of the TSC and will meet on the same day as the TSC to minimise costs and make best use of people’s time.

12.8 Investigator responsibilities

Prof Chris Salisbury is the Chief Investigator with overall responsibility for the conduct of the trial as well as specific responsibility for the local running of the lead recruiting site. In addition, principal

For peer review only - http://bmjopen.bmj.com/site/about/guidelines.xhtml

investigators will be responsible for the local running of the study at the remaining two recruiting centres (Pete Bower and Steward Mercer).

Sara Brookes (Senior Lecturer in Medical Statistics) will supervise the statistical analysis and provide trials methodology expertise. Sandra Hollinghurst (Senior Lecturer in Health Economics) will lead on economic methods. Cindy Mann (Senior Research Nurse) will conduct the process evaluation under supervision of Ali Heawood (Senior Research Fellow) and Bruce Guthrie, and will co-ordinate the PPI and advisory groups.

Stewart Mercer (Professor of Primary Care, Univ. of Glasgow) is an academic GP who will act as PI for Glasgow practices. Bruce Guthrie (Professor of Primary Care, Univ. of Dundee) is an academic GP with an interest in multimorbidity, guidelines and polypharmacy. He will co-lead the process evaluation with Ali Heawood.

Pete Bower is Professor and Head of the Centre for Primary Care at University of Manchester. He has expertise in trials and mixed methods research and a focus on mental health, multimorbidity, and service delivery. He will be PI for Manchester practices.

Other co-applicants Imran Rafi (Chair of Clinical Innovation & Research Centre, sits on RCGP council) and Adriana Gjini (NHS England, Public Health Consultant) will advise on the direction and progress of the study.

Investigator responsibilities (such as obtaining informed consent, investigator documentation and data collection) may be delegated to an appropriate member of study site staff such as Trial Manager or Trial Coordinators. Coordination between the sites is the responsibility of the Chief Investigator and Trial Manager.

13 Dissemination and projected outputs

This project will demonstrate whether or not the new approach to managing multimorbidity in general practice is effective and cost-effective. Our hypothesis is that the new approach will improve patient outcomes in terms of health related quality of life, reduced illness and treatment burden and improved patient experience of well-coordinated person centred care. We anticipate that the intervention will be broadly cost neutral or even reduce costs for the NHS as a whole. There will be an increased cost from longer consultations in general practice and the financial incentive to practices to provide these, but this may be offset by fewer recall appointments for different individual conditions, fewer prescriptions and possibly fewer hospital appointments or admissions.

The project will seek to maximise the impact of the research by adopting a model of knowledge transfer based on the WHO knowledge transfer framework for ageing and health. This identifies 6 critical factors which are known to influence research impact. These factors will be addressed by constructing an advisory group consisting of academic leads from the study and potential users, to create an early and continuous dialogue between investigators and beneficiaries. Age UK have agreed to support this group. The academic members of this group will have expertise in the presentation of evidence to a wide range of audiences at national and international conferences focused on multi-morbidity; they will be complemented by users from the health and social care sectors, interested charities and the policy sector who have experience in the care of those with multi-morbidity, plus older people themselves, in accordance with the guidelines of the NIHR Age

For peer review only - http://bmjopen.bmj.com/site/about/guidelines.xhtml

and Ageing Group. The group will identify and develop opportunities for the dissemination and exploitation of research findings and insights from the study, to a wide variety of audiences as described above.

As part of this research project we will produce guides for commissioners and for practices to enable them to implement the new approach speedily and widely throughout the country, if it is effective. Our collaboration with the RCGP Clinical Innovation and Research Centre will facilitate wide dissemination to practices, and the backing of the RCGP will help to generate momentum to encourage practices to take up this new approach.

We will therefore seek to disseminate the findings from this research to the following audiences:

13.1 Dissemination to Patients

We will produce a short newsletter of the study results which can be distributed to all trial participants. The study results will be made available on the study website. With the help of the University of Bristol press office, we will design press releases and distribute then to local and national newspapers, websites and patient organisations for long term conditions (e.g. Diabetes UK, British Heart Foundation). We will take advice from our patient representatives an engage their support in disseminating findings. This includes the use of social media such as Twitter which can point people to the results on the study website.

13.2 Dissemination to Health Care Professionals

One of the co-applicants (IR) is a member of the Royal College of General Practitioners (RCGP) Clinical Innovation and Research Centre (CIRC). In addition to helping to identify practices to participate and advising on the development of the intervention, CIRC will also help to publicise the study findings to the RCGP's 42,000 members. This can be achieved through its communications network, hosting a page about the study on the RCGP website and the production of paper and online good practice aids and tools. In addition, a national workshop on Multimorbidity will be hosted by CIRC to highlight the research and disseminate the study findings.

13.3 Dissemination to Commissioners and Policy Makers

Commissioners and policy makers will be invited to the CIRC national workshop as described above. They will be made aware of articles in the lay, professional and academic press. Policy and key decision makers will be targeted using co-applicants individual networks. A guide for commissioners will be produced and published with the help of the RCGP. The full NIHR project report will include an executive summary and a set summary powerpoint slides aimed at NHS managers.

13.4 Dissemination to academics

The results of this study will be submitted to peer reviewed, high impact medical journals and presented at academic conferences. We envisage, as a minimum, research papers on the study protocol, the randomised trial outcomes, an economic analysis of cost effectiveness, patient experience of the new intervention and usual care, and the process evaluation. It is the intention of the trial team to publish the main study results within a year of study completion. The final project report will be made available on the NIHR website and published in the NIHR journal series.

14 Study conduct and end of study responsibilities

For peer review only - http://bmjopen.bmj.com/site/about/guidelines.xhtml

14.1 Protocol Amendment

Any changes in research activity procedures, except those necessary to remove an apparent, immediate hazard to the participant, will be reviewed and approved by the Chief Investigator. Amendments to the protocol will be submitted to the REC and NHS R&Ds for approval. The sponsor will also be notified at this point.

Protocol amendments may be substantial (requiring full review and favourable ethical opinion from the REC) or minor (not requiring review). Only once the amendment has been approved by REC and trust R&Ds (or acknowledged in the case of a minor amendment) can the amended protocol be implemented.

14.2 Protocol Violations and Deviations

Researchers or investigators should not implement any deviation from the protocol without agreement from the Chief Investigator and with REC and R&D approval, except where necessary to eliminate an immediate hazard to trial participants.

In the event that a researcher inadvertently or needs to deviate from the protocol, the nature and reasons for the deviation will be recorded in a CRF or filenote to be kept in the study site file and a copy sent to the trial manager for the Trial Master File. If this necessitates a subsequent protocol amendment, this will be submitted to the REC and trust R&Ds for review and approval if appropriate.

14.3 End of study Archiving

All study documentation will be kept for a minimum of 5 years after the end of the final analysis of the study. All paper records will be stored in secure university storage facilities. Personal identifiable paper records (hard copy consent forms) will be kept separate from anonymised paper records (questionnaires) and will be stored in locked filing cabinets in locked offices. All electronic records will be stored on password protected servers on secure computer networks in the University of Bristol.

14.4 End study

Investigators and/or the TSC have the right at any time to terminate the study for clinical or administrative reasons.

The investigators, with the advice of the DMC will establish a set of criteria for stopping the study prematurely.

The end of the study will be reported to the REC within 90 days, or 15 days if the study is stopped early. The investigators will inform participants and ensure that the appropriate follow-up is arranged for all involved.

A draft final report and a final summary report is required by the funder within 14 days after the completion date of the programme or date of termination.

For peer review only - http://bmjopen.bmj.com/site/about/guidelines.xhtml

15 References

1. Salisbury C, Johnson L, Purdy S, Valderas JM, Montgomery AA (2011) Epidemiology and impact of multimorbidity in primary care: a retrospective cohort study. British Journal of General Practice 61.
2. Barnett K, Mercer SW, Norbury M, Watt G, Wyke S, et al. (2012) Epidemiology of multimorbidity and implications for health care, research, and medical education: a cross-sectional study. Lancet 380: 37-43.
3. Bayliss EA, Edwards AE, Steiner JF, Main DS (2008) Processes of care desired by elderly patients with multimorbidities. Family Practice 25: 287-293.
4. Fortin M, Lapointe L, Hudon C, Vanasse A, Ntetu AL, et al. (2004) Multimorbidity and quality of life in primary care: a systematic review. Health Qual Life Outcomes 2: 51.
5. Huntley AL, Johnson R, Purdy S, Valderas JM, Salisbury C (2012) Measures of Multimorbidity and Morbidity Burden for Use in Primary Care and Community Settings: A Systematic Review and Guide. Annals of Family Medicine 10: 134-141.
6. Perkins AJ, Kroenke K, Unutzer J, Katon W, Williams JW, Jr., et al. (2004) Common comorbidity scales were similar in their ability to predict health care costs and mortality. J Clin Epidemiol 57: 1040-1048.
7. Gunn JM, Ayton DR, Densley K, Pallant JF, Chondros P, et al. (2012) The association between chronic illness, multimorbidity and depressive symptoms in an Australian primary care cohort. Soc Psychiatry Psychiatr Epidemiol 47: 175-184.
8. Glynn LG, Valderas JM, Healy P, Burke E, Newell J, et al. (2011) The prevalence of multimorbidity in primary care and its effect on health care utilization and cost. Fam Pract 28: 516-523.
9. Payne RA, Abel GA, Guthrie B, Mercer SW (2013) The effect of physical multimorbidity, mental health conditions and socioeconomic deprivation on unplanned admissions to hospital: a retrospective cohort study. CMAJ 185: E221-228.
10. Marengoni A, Angleman S, Melis R, Mangialasche F, Karp A, et al. (2011) Aging with multimorbidity: a systematic review of the literature. Ageing Res Rev 10: 430-439.
11. Bayliss EA, Ellis JL, Steiner JF (2007) Barriers to self-management and quality-of-life outcomes in seniors with multimorbidities. Annals of Family Medicine 5: 395-402.
12. Noel PH, Frueh BC, Larme AC, Pugh JA (2005) Collaborative care needs and preferences of primary care patients with multimorbidity. Health Expect 8: 54-63.
13. O'Brien R, Wyke S, Guthrie B, Watt G, Mercer S (2011) An 'endless struggle': a qualitative study of general practitioners' and practice nurses' experiences of managing multimorbidity in socio-economically deprived areas of Scotland. Chronic Illn 7: 45-59.
14. Doran T, Kontopantelis E, Valderas JM, Campbell S, Roland M, et al. (2011) Effect of financial incentives on incentivised and non-incentivised clinical activities: longitudinal analysis of data from the UK Quality and Outcomes Framework. BMJ 342: d3590.
15. Salisbury C (2012) Multimorbidity: redesigning health care for people who use it. Lancet 380: 7-9.
16. Burgers JS, Voerman GE, Grol R, Faber MJ, Schneider EC (2010) Quality and coordination of care for patients with multiple conditions: results from an international survey of patient experience. Eval Health Prof 33: 343-364.
17. Bayliss EA, Steiner JF, Fernald DH, Crane LA, Main DS (2003) Descriptions of barriers to self-care by persons with comorbid chronic diseases. Ann Fam Med 1: 15-21.
18. Fortin M, Dionne J, Pinho G, Gignac J, Almirall J, et al. (2006) Randomized controlled trials: do they have external validity for patients with multiple comorbidities? Ann Fam Med 4: 104-108.
19. Guthrie B, Payne K, Alderson P, McMurdo MET, Mercer SW (2012) Adapting clinical guidelines to take account of multimorbidity. British Medical Journal 345.
20. Boyd CM, Darer J, Boult C, Fried LP, Boult L, et al. (2005) Clinical practice guidelines and quality of care for older patients with multiple comorbid diseases: implications for pay for performance. JAMA 294: 716-724.

For peer review only - http://bmjopen.bmj.com/site/about/guidelines.xhtml

21. Hughes LD, McMurdo ME, Guthrie B (2013) Guidelines for people not for diseases: the challenges of applying UK clinical guidelines to people with multimorbidity. Age Ageing 42: 62-69.
22. Marcum ZA, Gellad WF (2012) Medication adherence to multidrug regimens. Clin Geriatr Med 28: 287-300.
23. Bourgeois FT, Shannon MW, Valim C, Mandl KD (2010) Adverse drug events in the outpatient setting: an 11-year national analysis. Pharmacoepidemiol Drug Saf 19: 901-910.
24. Leendertse AJ, Egberts AC, Stoker LJ, van den Bemt PM, Group HS (2008) Frequency of and risk factors for preventable medication-related hospital admissions in the Netherlands. Arch Intern Med 168: 1890-1896.
25. Mercer SW, Gunn J, Bower P, Wyke S, Guthrie B (2012) Managing patients with mental and physical multimorbidity. British Medical Journal 345.
26. Bower P, Macdonald W, Harkness E, Gask L, Kendrick T, et al. (2011) Multimorbidity, service organization and clinical decision making in primary care: a qualitative study. Family Practice 28: 579-587.
27. Salisbury C (2013) Multimorbidity: time for action rather than words. Br J Gen Pract 63: 64-65.
28. Howe A (2012) Medical generalism. Why expertise in whole person medicine matters. London: Royal College of General Practitioners. 1-68 p.
29. Physicians RCo (2012) Hospitals on the Edge. London: Royal College of Physicians. 1-8 p.
30. NICE (2012) Nice should consider multimorbidity in guidelines.
31. American Geriatrics Society Expert Panel on the Care of Older Adults with M (2012) Patient-centered care for older adults with multiple chronic conditions: a stepwise approach from the American Geriatrics Society: American Geriatrics Society Expert Panel on the Care of Older Adults with Multimorbidity. J Am Geriatr Soc 60: 1957-1968.
32. (2010) Improving the health and well-being of people with long term conditions. In: Health Do, editor. pp. 1-67.
33. Smith SM, Soubhi H, Fortin M, Hudon C, O'Dowd T (2012) Managing patients with multimorbidity: systematic review of interventions in primary care and community settings. British Medical Journal 345.
34. Cowie L, Morgan M, White P, Gulliford M (2009) Experience of continuity of care of patients with multiple long-term conditions in England. J Health Serv Res Policy 14: 82-87.
35. Fried TR, Tinetti ME, Iannone L (2011) Primary care clinicians' experiences with treatment decision making for older persons with multiple conditions. Arch Intern Med 171: 75-80.
36. Fortin M, Soubhi H, Hudon C, Bayliss EA, van den Akker M (2007) Multimorbidity's many challenges. British Medical Journal 334: 1016-1017.
37. Smith SM, O'Kelly S, O'Dowd T (2010) GPs' and pharmacists' experiences of managing multimorbidity a 'Pandora's box'. British Journal of General Practice 60.
38. Gruffydd-Jones K, Poduval S (2013) Managing multimorbidity in practice: what lessons can be learnt from the care of people with COPD and co-morbidities? Leicester: NHS Improvement. 1-24 p.
39. Craig P, Dieppe P, Macintyre S, Michie S, Nazareth I, et al. (2008) Developing and evaluating complex interventions: the new Medical Research Council guidance. BMJ 337: a1655.
40. Stewart M (2001) Towards a global definition of patient centred care. BMJ 322: 444-445.
41. Little P, Everitt H, Williamson I, Warner G, Moore M, et al. (2001) Preferences of patients for patient centred approach to consultation in primary care: observational study. BMJ 322: 468-472.
42. Little P, Everitt H, Williamson I, Warner G, Moore M, et al. (2001) Observational study of effect of patient centredness and positive approach on outcomes of general practice consultations. BMJ 323: 908-911.
43. Stewart M, Brown JB, Donner A, McWhinney IR, Oates J, et al. (2000) The impact of patient-centered care on outcomes. J Fam Pract 49: 796-804.

For peer review only - http://bmjopen.bmj.com/site/about/guidelines.xhtml

44. Stewart MA (1995) Effective physician-patient communication and health outcomes: a review. CMAJ 152: 1423-1433.
45. Mead N, Bower P (2002) Patient-centred consultations and outcomes in primary care: a review of the literature. Patient Educ Couns 48: 51-61.
46. Mead N, Bower P (2000) Patient-centredness: a conceptual framework and review of the empirical literature. Soc Sci Med 51: 1087-1110.
47. Hudon C, Fortin M, Haggerty JL, Lambert M, Poitras ME (2011) Measuring patients' perceptions of patient-centered care: a systematic review of tools for family medicine. Ann Fam Med 9: 155-164.
48. Nutting PA, Goodwin MA, Flocke SA, Zyzanski SJ, Stange KC (2003) Continuity of primary care: to whom does it matter and when? Ann Fam Med 1: 149-155.
49. Wagner EH, Austin BT, Von Korff M (1996) Improving outcomes in chronic illness. Manag Care Q 4: 12-25.
50. Young B, Roberts S (2011) Year of Care: Report of findings from the pilot programme. NHS Networks. 1-158 p.
51. Edwards PJ, Roberts I, Clarke MJ, Diguiseppi C, Wentz R, et al. (2009) Methods to increase response to postal and electronic questionnaires. Cochrane Database Syst Rev: MR000008.
52. Bower P, Gilbody S (2005) Stepped care in psychological therapies: access, effectiveness and efficiency. Narrative literature review. Br J Psychiatry 186: 11-17.
53. Patterson SM, Hughes C, Kerse N, Cardwell CR, Bradley MC (2012) Interventions to improve the appropriate use of polypharmacy for older people. Cochrane Database Syst Rev 5: CD008165.
54. Viswanathan M, Golin CE, Jones CD, Ashok M, Blalock SJ, et al. (2012) Interventions to improve adherence to self-administered medications for chronic diseases in the United States: a systematic review. Ann Intern Med 157: 785-795.
55. Avery AJ, Rodgers S, Cantrill JA, Armstrong S, Cresswell K, et al. (2012) A pharmacist-led information technology intervention for medication errors (PINCER): a multicentre, cluster randomised, controlled trial and cost-effectiveness analysis. Lancet 379: 1310-1319.
56. Wachter RM, Bell D (2012) Renaissance of hospital generalists. BMJ 344: e652.
57. Board NC (2012) Enhanced Services Commissioning Fact Sheet.
58. Hawe P, Shiell A, Riley T (2004) Complex interventions: how "out of control" can a randomised controlled trial be? BMJ 328: 1561-1563.
59. EQ5D; A standardised instrument for use as a measure of health outcome.
60. Bayliss EA, Ellis JL, Steiner JF (2009) Seniors' self-reported multimorbidity captured biopsychosocial factors not incorporated into two other data-based morbidity measures. Journal of Clinical Epidemiology 62: 550-557.
61. Reeves D, Campbell SM, Adams J, Shekelle PG, Kontopantelis E, et al. (2007) Combining multiple indicators of clinical quality: an evaluation of different analytic approaches. Med Care 45: 489-496.
62. Zigmond AS, Snaith RP (1983) The hospital anxiety and depression scale. Acta Psychiatr Scand 67: 361-370.
63. Boyd CM, Wolff JL, Giovannetti E, Reider L, Weiss C, et al. (2014) Healthcare Task Difficulty Among Older Adults with Multimorbidity. Medical Care 52: S118-S125.
64. Morisky DE, Green LW, Levine DM (1986) Concurrent and Predictive-Validity of a Self-Reported Measure of Medication Adherence. Medical Care 24: 67-74.
65. Guthrie B, McCowan C, Davey P, Simpson CR, Dreischulte T, et al. (2011) High risk prescribing in primary care patients particularly vulnerable to adverse drug events: cross sectional population database analysis in Scottish general practice. BMJ 342: d3514.
66. Mercer SW, McConnachie A, Maxwell M, Heaney D, Watt GCM (2005) Relevance and practical use of the Consultation and Relational Empathy (CARE) Measure in general practice. Family Practice 22: 328-334.

For peer review only - http://bmjopen.bmj.com/site/about/guidelines.xhtml

For peer review only

67. Rick J, Rowe K, Hann M, Sibbald B, Reeves D, et al. (2012) Psychometric properties of the patient assessment of chronic illness care measure: acceptability, reliability and validity in United Kingdom patients with long-term conditions. Bmc Health Services Research 12.
68. Al-Janabi H, Flynn TN, Coast J (2011) Estimation of a preference-based carer experience scale. Med Decis Making 31: 458-468.
69. Kind P, Hardman G, Macran S (1999) Population norms for EQ-5D. York: The University of York. Discussion paper 172 Discussion paper 172. 1-98 p.
70. Walters SJ, Brazier JE (2005) Comparison of the minimally important difference for two health state utility measures: EQ-5D and SF-6D. Qual Life Res 14: 1523-1532.
71. Curtis L (2012) Unit costs of Health and Social Care. Canterbury, Kent: Personal Social Services Research Unit. 1-272 p.
72. Grant A, Treweek S, Dreischulte T, Foy R, Guthrie B (2013) Process evaluations for cluster-randomised trials of complex interventions: a proposed framework for design and reporting. Trials 14: 15.
73. Oakley A, Strange V, Bonell C, Allen E, Stephenson J, et al. (2006) Process evaluation in randomised controlled trials of complex interventions. BMJ 332: 413-416.
74. Yin RK (2003) Case Study Research: Design and Methods. London: Sage.
75. Bice TW, Boxerman SB (1977) A quantitative measure of continuity of care. Med Care 15: 347-349.
76. Ritchie J, Spencer L. (1994) Qualitative data analysis for applied policy research. In Analyzing qualitative data. Edited by Bryman A and Burgess RG. London: Routledge
77. Miles MB, Boxerman SB (1994) Qualititative Data Analysis. Thousand Oaks: Sage.

For peer review only

For peer review only - http://bmjopen.bmj.com/site/about/guidelines.xhtml

16 Appendix A – Trial Contacts

(Alphabetical by surname)

**Professor Pete Bower**<br><br>Professor of Health Services Research<br><br>Centre for Primary Care<br>Institute of Population Health<br>University of Manchester<br>Williamson Building<br>Oxford Road<br>Manchester M13 9PL<br>Tel: (0)161 275 7638<br><br>Email: peter.bower@manchester.ac.uk	**Dr. Sara Brookes**<br><br>Senior Lecturer in Medical Statistics,<br><br>School of Social and Community Medicine,<br><br>Canynge Hall<br><br>39 Whatley Road<br><br>Bristol BS8 2PS<br><br>Tel: (0117) 928 7218<br><br>Email: Sara.T.Brookes@bristol.ac.uk
**Dr. Ardiana Gjini**<br><br>Consultant in Public Health:<br><br>NHS England Bath, Gloucestershire, Swindon & Wiltshire Area Team<br><br>Tel:<br><br>Email: a.gjini@nhs.net	**Professor Bruce Guthrie**<br><br>Professor of Primary Care Medicine<br><br>Quality, Safety & Informatics Research Group,<br><br>Mackenzie Building,<br><br>Kirsty Semple Way,<br><br>Dundee DD2 4BF<br><br>Tel:<br><br>Email: b.guthrie@dundee.ac.uk
**Dr. Ali Heawood**<br><br>Senior Research Fellow in Primary Care<br><br>School of Social and Community Medicine,<br><br>Canynge Hall<br><br>39 Whatley Road<br><br>Bristol BS8 2PS<br><br>Tel: (0117) 331 3934<br><br>Email: Ali.Heawood@bristol.ac.uk	**Dr. Sandra Hollinghurst**<br><br>Senior Lecturer in Health Economics<br><br>School of Social and Community Medicine,<br><br>Canynge Hall<br><br>39 Whatley Road<br><br>Bristol BS8 2PS<br><br>Tel: (0117) 331 3901<br><br>Email: S.P.Hollinghurst@bristol.ac.uk
**Cindy Mann**<br><br>Research Associate<br><br>School of Social and Community Medicine,	**Professor Stewart Mercer**<br><br>Chair in Primary Care Research (General Practice and Primary Care)<br><br>Institute of Health and Wellbeing

For peer review only - http://bmjopen.bmj.com/site/about/guidelines.xhtml

Canynge Hall<br>39 Whatley Road<br>Bristol BS8 2PS<br>Tel: (0117) 928 7372<br>Email: Cindy.Mann@bristol.ac.uk	College of Medical, Veterinary and Life Sciences<br>University of Glasgow<br>1 Horslethill Road<br>Glasgow G12 9LX<br>Tel: 01413308330<br>Email: Stewart.Mercer@glasgow.ac.uk
**Dr. Imran Rafi**<br>Chair of RCGP Clinical Innovation and Research<br>Royal College of General Practitioners<br>30 Euston Square<br>London<br>NW1 2FB<br>Tel: 020 3188 7400<br>Email: imran.rafi@rcgp.org.uk	**Professor Christopher Salisbury**<br>Professor in Primary Health Care<br>School of Social and Community Medicine,<br>Canynge Hall<br>39 Whatley Road<br>Bristol BS8 2PS<br>Tel: (0117) 3314530<br>Email: C.Salisbury@bristol.ac.uk
**Dr. Joanna Thorn, Health Economist**<br>Research Associate in Health Economics<br>School of Social and Community Medicine,<br>Canynge Hall<br>39 Whatley Road<br>Bristol<br>BS8 2PS<br>Tel: (0117)3314526<br>Email: Joanna.Thorn@bristol.ac.uk	

For peer review only

Appendix B – Trial Timeline

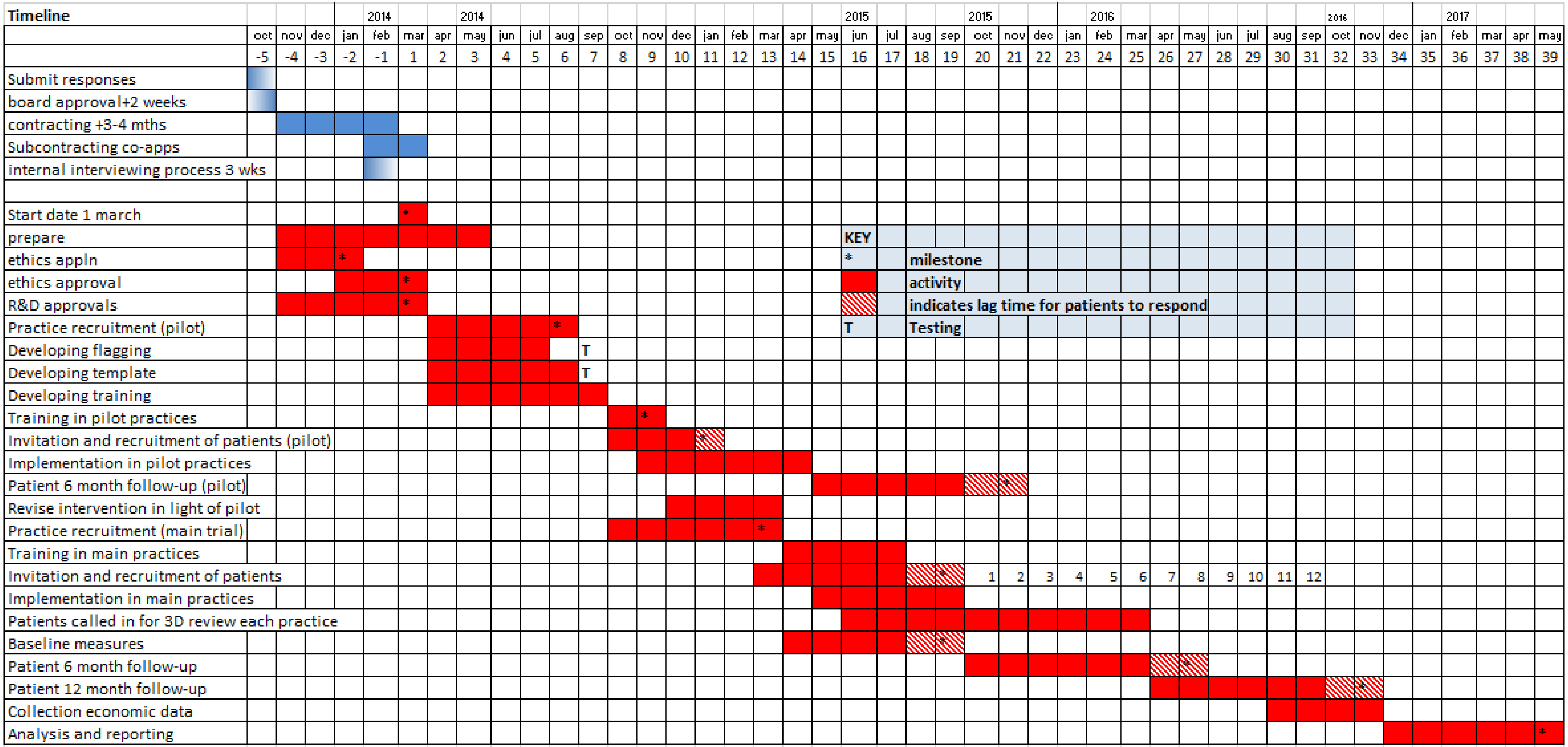

17 Appendix C – Study Flow Diagram & Conceptual Framework

Flow Diagram. HS & DR project 12/130/15

Improving the management of patients with multimorbidity in general practice. Salisbury et al.

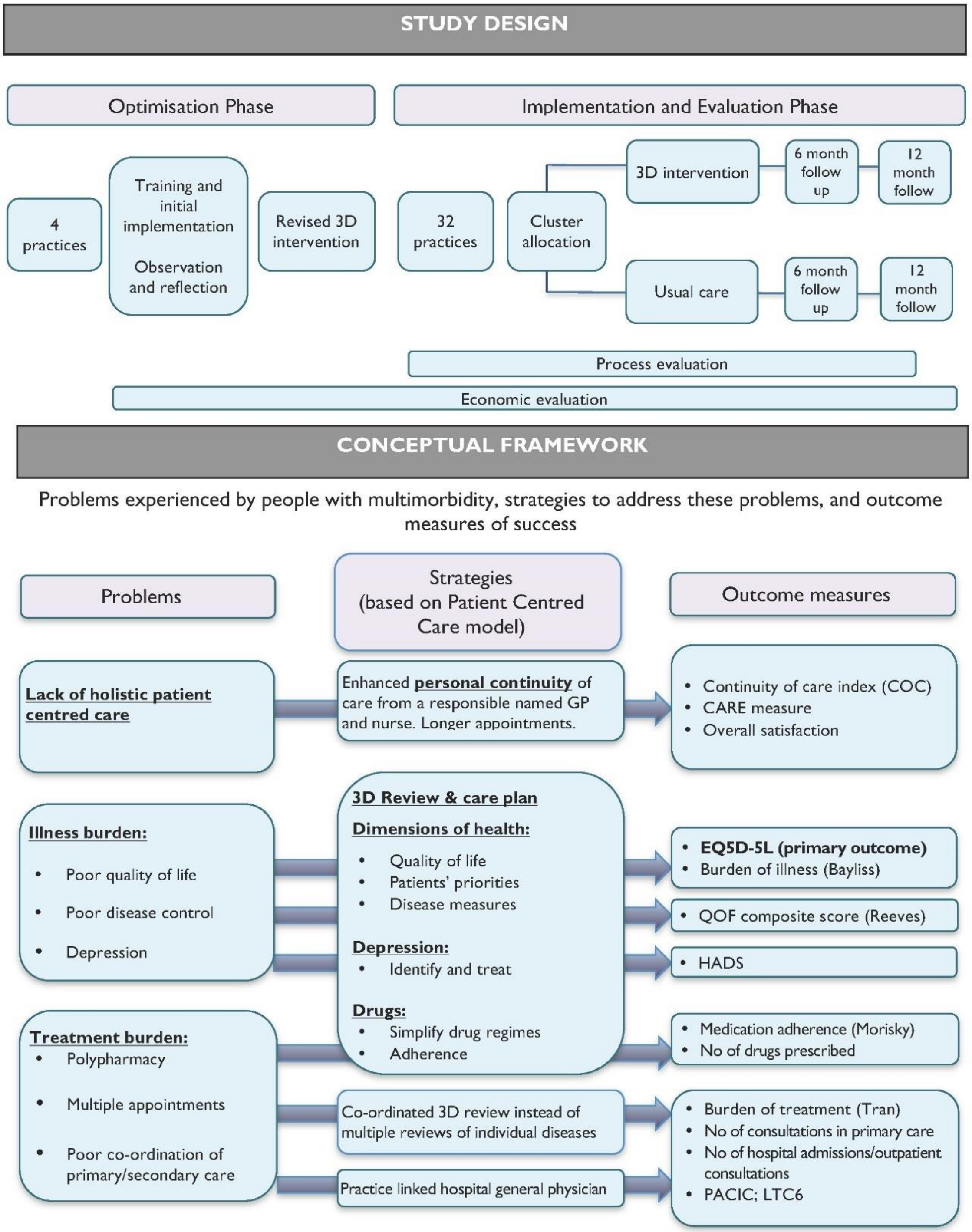

18 Appendix D – Participant flowchart

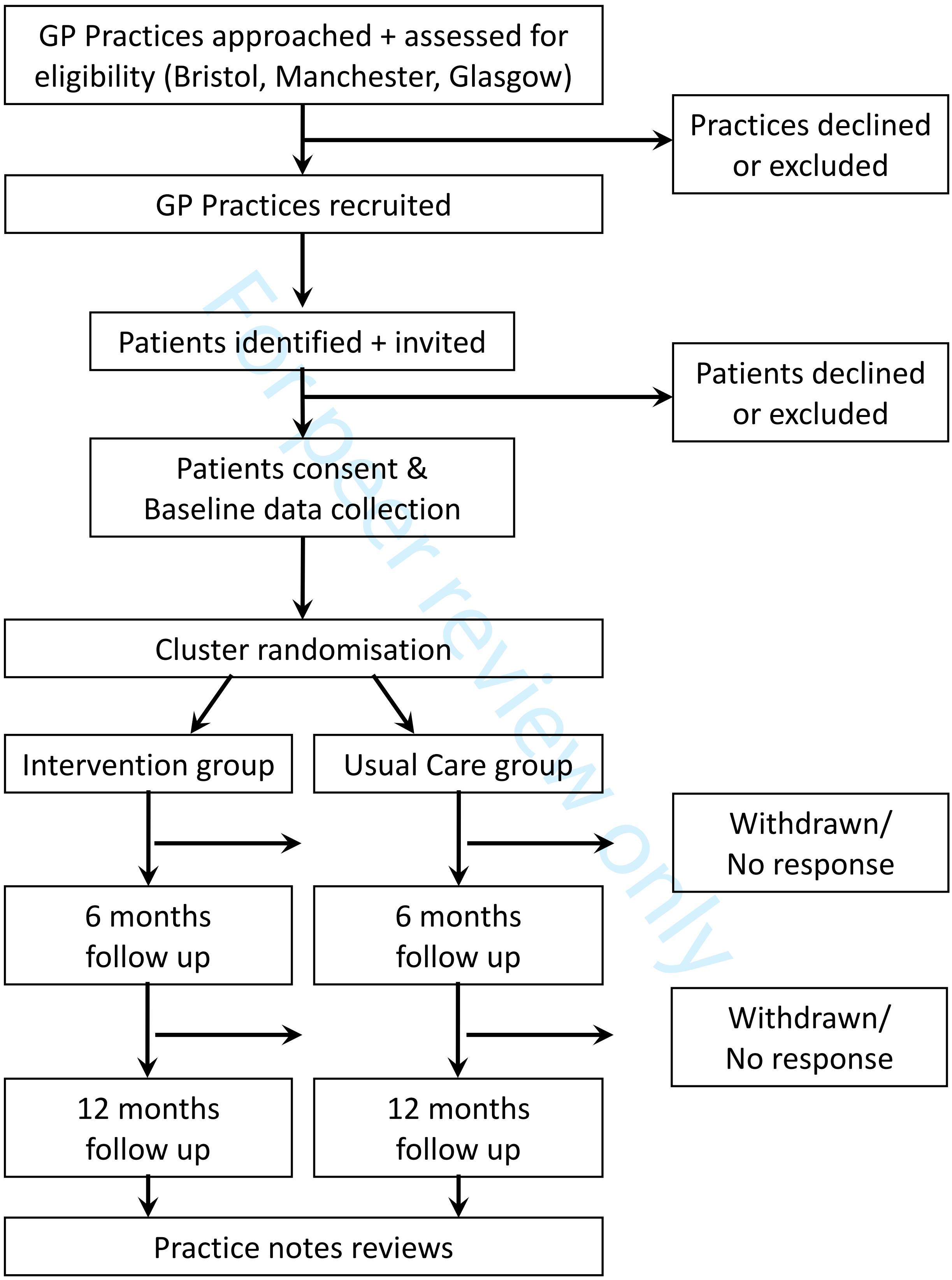

For peer review only - http://bmjopen.bmj.com/site/about/guidelines.xhtml

Summary of changes to protocol and trial registry

Amendments to the study protocol, all approved by ethics committee.

REC Amendment/ Protocol Number	Trial Design	Description of change
Amendment 10, Protocol v6.0	Follow-up timepoints	Originally 6 and 12 months post recruitment in pilot practices, but changed to 9 and 15 months in main study. This was to allow for the fact that it took approximately 3 months after practice randomisation before the practice began to offer the intervention to patients due to delays in arranging staff training. This change was made after the main study began but before the final protocol was published and before any patients reached their first follow-up time-point.
Amendments 2, 7, 11, Protocol 6.1	Process Evaluation	(Not relevant to this paper) Increase number and clarify optional observation/audio or video recording of review consultations with patients and health care professionals in intervention and usual care practices to enhance assessment of variation in reviews.
Amendment 12, Protocol v7.1	Pharmacy review substudy	(Not relevant to this paper). Inclusion of additional qualitative substudy exploring GPs and pharmacists' views of the 3D pharmacy reviews

For peer review only - http://bmjopen.bmj.com/site/about/guidelines.xhtml

Changes to trial registry: ISRCTN06180958

Date	Stage of trial	Changes
28/04/2015	Following pilot study	• Adults lacking capacity to consent in Scotland were added as an exclusion criterion, due to ethical committee advice.<br>• Inclusion criterion were modified following the pilot study to remove osteoporosis and to combine chronic kidney disease within the cardiovascular disease group<br>• Change in measure of treatment burden from Tran to MTBQ<br>• Omission of two questions from LTC6 questionnaire about care being joined up and patient discussing their most important problems (administrative error in updating registry; reinstated later)
19/01/2016	During trial recruitment, before any follow-up or analysis	• Follow-up time points changed from 6 and 12 months to 9 and 15 months, in light of experience of lag time before practices started delivering intervention.<br>• EQ5D at 9 months specified as a secondary outcome (EQ5D at 15 months is primary outcome)
02/02/2017	On completing statistical analysis plan, before any analysis.	• More detailed specification of measures used for each outcome, due to updated requirements of registry.<br>• Two questions about care coordination and care relating to patient's priorities from LTC6 reinstated as outcomes (omitted from registry during changes of 28/04/15 by mistake)<br>• Continuity of care (an important aim of the intervention and specified in the SAP but not previously specified in registry due to oversight)<br>• Number of high risk prescribing indicators added (always intended and specified in protocol but reliable measures only became possible during the trial)<br>• Cost-effectiveness. Already included in the protocol, and aims of the study in the trial registry, but now specified as an outcome rather than as an approach to analysis (because of recent experience of publishing another trial, where a journal required that cost-effectiveness be listed as an outcome in the registry).

For peer review only

For peer review only

For peer review only - http://bmjopen.bmj.com/site/about/guidelines.xhtml

BMJ Open

Cost-effectiveness of a patient-centred approach to managing multimorbidity in primary care: a pragmatic cluster randomised controlled trial

Journal:	*BMJ Open*
Manuscript ID	bmjopen-2019-030110.R1
Article Type:	Original research
Date Submitted by the Author:	24-Jul-2019
Complete List of Authors:	Thorn, Joanna; University of Bristol, Population Health Sciences<br>Man, Mei-See; University of Bristol School of Social and Community Medicine,<br>Chaplin, Katherine ; University of Bristol Centre for Academic Primary Care, Bristol Medical School<br>Bower, Peter; University of Manchester, NPCRDC<br>Brookes, Sara; Institute of Cancer and Genomic Sciences, University of Birmingham, Cancer Research UK Clinical Trials Unit ; University of Bristol, Population Health Sciences<br>Gaunt, Daisy; University of Bristol Faculty of Medicine and Dentistry, Bristol Randomised Trials Collaboration & School of Social and Community Medicine; University of Bristol<br>Fitzpatrick, Bridie; University of Glasgow, Institute of Health and Wellbeing<br>Gardner, Caroline; University of Manchester Faculty of Biology, Medicine and Health<br>Guthrie, Bruce; University of Dundee, Population Health Sciences Division, Medical Research Institute<br>Hollinghurst, Sandra; University of Bristol, Community Based Medicine<br>Lee, Victoria; University of Manchester Faculty of Biology, Medicine and Health<br>Mercer, Stewart; University of Glasgow, Institute of Health and Wellbeing<br>Salisbury, Chris; University of Bristol, Academic Unit of Primary Health Care
**Primary Subject Heading**:	Health economics
Secondary Subject Heading:	General practice / Family practice
Keywords:	Multimorbidity, PRIMARY CARE, patient-centred care, economic evaluation

SCHOLARONE™
Manuscripts

Cost-effectiveness of a patient-centred approach to managing multimorbidity in primary care: a pragmatic cluster randomised controlled trial

Joanna C Thorn[1], Mei-See Man[1,2], Katherine Chaplin[1], Peter Bower[3], Sara T Brookes[2], Daisy Gaunt[2], Bridie Fitzpatrick[4], Caroline Gardner[3], Bruce Guthrie[5], Sandra Hollinghurst[1], Victoria Lee[3], Stewart W Mercer[6], Chris Salisbury[1]

[1] Centre for Academic Primary Care, Population Health Sciences, Bristol Medical School, University of Bristol, Canynge Hall, 39 Whatley Road, Bristol, BS8 2PS, UK
[2] Bristol Randomised Trials Collaboration, Population Health Sciences, University of Bristol, Canynge Hall, 39 Whatley Road, Bristol, BS8 2PS, UK
[3] NIHR School for Primary Care Research, Centre for Primary Care and Health Services Research, Faculty of Biology, Medicine and Health, University of Manchester, Manchester Academic Health Science Centre, UK
[4] Institute of Health and Wellbeing, University of Glasgow, 1 Lilybank Gardens, Glasgow, G12 8RZ, UK
[5] Division of Population Health Sciences, Medical Research Institute, University of Dundee, Dundee, UK
[6] Usher Institute, University of Edinburgh, Old Medical School, Teviot Place, Edinburgh, EH8 9AG, UK

Corresponding author: Dr Joanna Thorn, Population Health Sciences, University of Bristol, 1-5 Whiteladies Road, Bristol, BS8 1NU, UK; joanna.thorn@bristol.ac.uk; Tel. +44 (0) 117 4283125

3969 words

Funding: This project was funded by the National Institute for Health Research (NIHR) Health Services and Delivery Research Programme (project number 12/130/15). This study was designed and conducted in collaboration with the Bristol Randomised Trials Collaboration (BRTC), a UKCRC Registered clinical trials unit (CTU) in receipt of NIHR CTU support funding. CS is partly supported by The NIHR Collaboration for Leadership in Applied Health Research and Care West (CLAHRC West) and by Bristol Clinical Commissioning Group.

The views and opinions expressed in this report are those of the authors and do not necessarily reflect those of the NIHR, the NHS or the Department of Health.

Keywords: Multimorbidity, primary care, economic evaluation, patient-centred care

Running title: Multimorbidity management in primary care

For peer review only - http://bmjopen.bmj.com/site/about/guidelines.xhtml

Acknowledgments: We would like to thank the patients and practices who participated in the study, the patients and carers who contributed to the advisory group and the other members of the 3D study team (Cindy Mann, Polly Duncan, Keith Moffatt, Emma Moody, Imran Rafi, Becca Robinson, Ali Heawood, Bryar Kadir, John McLeod). We would also like to thank Professor Joanna Coast and Dr Padraig Dixon for valuable guidance. This article is an abbreviated version of the final report to the funders (Salisbury C, Man M-S, Chaplin K, Mann C, Bower P, Brookes S, et al. A patient-centred intervention to improve the management of multimorbidity in general practice: the 3D RCT. *Health Serv Deliv Res* 2019;7(5)).

Author contributions

CS, PB, BG, STB, SH and SWM conceived, designed and interpreted results from the 3D trial. SH and JCT designed the economic evaluation. M-SM (trial manager), KC, BF, CG and VL contributed to local economic data collection and interpretation of results. DG (statistician) conducted the multiple imputation. JCT carried out the analysis and wrote the paper, and all authors revised it for important intellectual content.

Competing interests statement

CS reports grants from CLAHRC West and Bristol CCG outside the submitted work. No other competing interests are declared.

For peer review only - http://bmjopen.bmj.com/site/about/guidelines.xhtml
For peer review only

ABSTRACT

Objective Patients with multiple chronic health conditions are often managed in a disjointed fashion in primary care, with annual review clinic appointments offered separately for each condition. This study aimed to determine the cost-effectiveness of the 3D intervention, which was developed to improve the system of care.

Design Economic evaluation conducted alongside a pragmatic cluster-randomised trial.

Setting General practices in three centres in England and Scotland.

Participants 797 adults with three or more chronic conditions were randomised to the 3D intervention, while 749 participants were randomised to receive usual care.

Intervention The 3D approach: comprehensive 6-monthly GP consultations, supported by medication reviews and nurse appointments.

Primary and secondary outcome measures The primary economic evaluation assessed the cost per quality-adjusted life year (QALY) gained from the perspective of the NHS and personal social services (PSS). Costs were related to changes in a range of secondary outcomes (QALYs accrued by both participants and carers, and deaths) in a cost–consequences analysis from the perspectives of the NHS/PSS, patients/carers and societal productivity.

Results

Very small increases were found in both QALYs (adjusted mean difference 0.007 [-0.009 to 0.023]) and costs (adjusted mean difference £126 [£-739 to £991]) in the intervention arm compared with usual care after 15 months. The incremental cost-effectiveness ratio was £18,499, with a 50.8% chance of being cost-effective at a willingness-to-pay threshold of £20,000 per QALY (55.8% at £30,000 per QALY).

Conclusions

The small differences in costs and outcomes were consistent with chance, and the uncertainty was substantial; therefore, the evidence for the cost-effectiveness of the 3D approach from the NHS/PSS perspective should be considered equivocal.

For peer review only

Article summary

Strengths and limitations of this study

- This economic evaluation was conducted alongside the largest randomised controlled trial of an intervention for managing multimorbid patients in primary care.
- Data collection was meticulous and high questionnaire return rates were achieved.
- Data on the use of care homes, which may contribute significantly to social care costs, were not available to include in the economic evaluation.
- Estimates of healthcare costs for this type of intervention have wide confidence intervals, even with a substantial sample size.

For peer review only

Data sharing statement

Requests for data sharing should be submitted to the Professor C Salisbury (c.salisbury@bristol.ac.uk) for consideration. Access to anonymised data might be granted following review via the University of Bristol Research Data Storage Facility.

For peer review only - http://bmjopen.bmj.com/site/about/guidelines.xhtml

INTRODUCTION

The number of patients living with multiple chronic health conditions (multimorbidity) is increasing in developed countries as the population ages[1]. Multimorbidity is associated with poor health-related quality of life[2 3], higher use of health services and higher costs[4 5]. Multimorbidity is common for older adults (with a prevalence of up to 98%[4]), leading to a substantial economic burden[6].

Care pathways for patients with multimorbidity are often poorly co-ordinated and burdensome for patients. Each condition may be treated separately, which fails to take account of interactions between diseases[1]. There have been relatively few randomised controlled trials (RCTs) of interventions designed to address issues affecting multimorbid patients, with mixed findings leading to substantial uncertainty about the effectiveness of such interventions[7]. Evidence on the cost-effectiveness of interventions remains even more limited and the evidence that is available is inconsistent[7-9]. A recent review of comprehensive care programmes for multimorbidity found no evidence that such interventions reduce healthcare costs or primary care visits[10]. Several reviews have highlighted the need for research on new interventions, including assessment of cost-effectiveness[1 7 11].

The ‘3D’ intervention was developed to address the issues associated with managing patients with multimorbidity in primary care in the UK. The intervention was based on a patient-centred care model and aimed to improve continuity of care with caregivers in GP surgeries, reduce patient burden in accessing healthcare and increase patient involvement in decision-making about their care (see Box 1). Based on the seminal work of Stewart[12], the concept of patient-centred care includes a focus on the patient’s individual illness experience, an integrated biopsychosocial perspective, seeking to find common ground with the patient and agreeing management plans, and enhancing a continuing relationship between the patient and doctor. We sought to apply these principles within the 3D intervention. The 3D randomised trial assessed the effectiveness of the 3D approach compared with usual care, and incorporated an embedded process evaluation. The trial did not find any differences in health-related quality of life over 15 months of follow-up, but the intervention did lead to improved patient-centred care[13 14]. Here, we report the results of the economic evaluation conducted alongside the 3D trial.

For peer review only - http://bmjopen.bmj.com/site/about/guidelines.xhtml

Box 1.

The 3D model

3D is a name used to allude to the concept of wholeness, while also acting as a mnemonic for ‘dimensions of health, depression and drugs’.

- Based on a patient centred care model.
- Practice level changes to improve continuity of care and to replace disease-focused reviews of each health condition with one six-monthly comprehensive 3D review with a named GP.
- Each 3D review consisted of three elements
    1) Nurse consultation to identify health problems most important to the patient, issues with quality of life, screening for depression, collecting health data e.g. blood pressure and information relevant to the patient’s specific conditions.
    2) Pharmacist review of medication from medical records, aiming to simplify and optimise drug treatment. Pharmacists were asked to identify non-essential drugs that could be stopped and essential drugs that should be started, and to seek ways to simplify drug treatment regimes e.g. by making all doses once daily.
    3) GP reviewed data from nurse and pharmacist, and agreed a health plan with the patient, which was given to them as a printed copy.
- An interactive computerised template enabled sharing of data between the clinicians, tailored the review to the patient’s specific conditions, contained automated checks, and generated printed summaries to enable sharing of information and plans with the patient.
- Strategies to encourage implementation were used, including training, monthly feedback about implementation, and financial incentives for completed reviews.

Usual care in the UK

- Review of chronic conditions is mainly carried out by nurses in primary care using disease-specific data-entry screens or templates.
- Nurses commonly specialise in particular conditions and review each disease separately.
- Chronic disease reviews mainly focus on meeting the requirements of the QOF pay-for-performance scheme.

For peer review only - http://bmjopen.bmj.com/site/about/guidelines.xhtml

METHODS

Design: The 3D trial

Full details of the study design, eligibility criteria, recruitment process and clinical effectiveness results are given elsewhere[14 15]. A favourable ethical opinion was granted by the South-West (Frenchay) National Health Service (NHS) Research Ethics Committee (14/SW/0011), and the trial was prospectively registered (ISCRTN06180958). In brief, the pragmatic 3D cluster randomised trial compared the effectiveness and cost-effectiveness of a complex intervention for multimorbidity with usual care delivered in 33 general practices in Scotland and England. The target patients were adults with multimorbidity, defined as having three or more chronic conditions from those included in the NHS Quality and Outcomes Framework[16]. Clinical staff in intervention practices attended two half-day 3D training sessions. Participants in the intervention arm were offered six-monthly holistic reviews of their health problems. Each review consisted of a nurse review appointment to collect relevant clinical information, a medication review conducted by a pharmacist and an extended appointment for the patient to discuss their conditions with a named GP. An individual health plan was negotiated with patients, detailing their own priorities for managing their conditions. The primary outcome measure for effectiveness was health-related quality of life at 15 months after randomisation, measured using the EQ-5D-5L[17].

The primary economic cost–utility analysis was conducted from the NHS and personal social services (NHS/PSS) perspective over 15 months of follow-up. A secondary analysis was conducted from the perspective of patients and carers.

Patient and public involvement

An active group of up to 14 patients and carers provided a service user perspective, contributing to refinement of the research questions, design of the intervention, design of outcome measures, analysis of qualitative data, patient newsletters, the study website and interpretation of findings. All participants were given a summary of the findings and a link to the published paper describing the main results. One of the outcome measures in the study was treatment burden and this was compared between trial arms.

Measures

Outcome measurement

The primary economic outcome measure was quality-adjusted life years (QALYs). EQ-5D-5L measurements were collected by postal questionnaires at baseline, 9 months and 15 months post-recruitment, supplemented by telephone collection for non-responders. Utility scores based on a UK population were derived from responses to the EQ-5D-5L cross-mapped to valuations obtained for the EQ-5D-3L instrument[18]. This was a change (approved by the Independent Data Monitoring Committee) to the analysis originally planned, as the National Institute for Health and Care Excellence (NICE) issued a

For peer review only - http://bmjopen.bmj.com/site/about/guidelines.xhtml

position statement recommending this approach[19] subsequent to the design of the study but prior to analysis. QALYs over the 15-month period were formed from these valuations by means of linear interpolation and an area under the curve calculation. Patients who died during follow-up were treated as if their most recent utility score was relevant until death, and set to zero immediately at death. Carers also completed the EQ-5D-5L instrument at the same timepoints as patients.

Resource-use measurement

As the trial population had multimorbidity, resource use related to any health condition experienced by the participant was considered relevant.

Staff training attendance records were kept to track resources used to deliver the training programmes for GPs, nurses and receptionists, including trainee and trainer delivery time (and preparation time), travel costs, and course materials. 3D GP appointments, nurse appointments and pharmacist reviews were captured by downloading routine GP records, supplemented by manual data capture by researchers reviewing participants' medical records at the end of the trial.

Data on medications prescribed and tests/investigations in primary care were extracted from downloads of routine GP records. Details of the number and duration of primary care consultations (other than 3D reviews) were similarly extracted. These included face-to-face, telephone and home consultations with doctors, nurses or health care assistants based in general practice. Duration details were not available for practices in Scotland. Therefore, an average duration for each type of consultation by each staff type in each arm was derived using available data (practices in England only) and applied to all relevant consultations. The research team collected NHS secondary care data from participants' GP records.

To assess travel costs, the patient's normal transport method for GP appointments and the cost (for public transport) or mileage (for private transport) were collected in the patient-reported questionnaire at baseline, along with usual practice with regards to time off work, and details of whether the participant paid prescription charges or not. NHS community care, care from social services, time off work for patients and carers to attend hospital appointments and over-the-counter medication expenditure were captured in the patient-reported questionnaires at 9 and 15 months, as were private use of treatments and therapies.

Valuation of resource use

Unit costs for NHS staff time for training and delivery of the intervention were based on the most recently available national estimates[20]. Actual expenses relating to training materials, refreshments and staff travel were recorded. Because only some GPs and a small proportion of patients in each practice participated in the trial, costs were adjusted to estimate the cost of training a full practice, divided by the

For peer review only - http://bmjopen.bmj.com/site/about/guidelines.xhtml
For peer review only

number of eligible patients in the practice and annualised over an estimated 5-year period of relevance. Medication costs were downloaded directly as part of the routine GP records, supplemented by estimates from the British National Formulary where necessary[21]. Patient prescription charges were subtracted from the NHS perspective medication costs. Community and primary care costs were based on recent national estimates[20].

Secondary care contacts were assigned to a healthcare resource group (HRGs, groups of events that have been judged to consume similar levels of resources) and costed based on national reference costs[22]. Productivity costs for both patients and carers were estimated based on average weekly earnings stratified by age group[23]. Mileage costs were estimated using UK government allowances[24]. Costs for over-the-counter medication and private therapies and treatments were all reported directly by patients. Unit costs used in the analyses are detailed in appendix 1.

All costs were reported in 2015/2016 pounds sterling, adjusted for inflation where necessary[25]. Costs and outcomes occurring during the final three months of follow-up were discounted in line with NICE guidance (currently 3.5%)[26]. Dates were not available for all types of resource use measured in the trial; in these cases, 50% of the costs incurred in the final six months of follow-up were subjected to discounting.

Data cleaning and missing costs and outcomes

Data cleaning was undertaken prior to unblinding. Questionnaires were not classed as 'missing data' for the cost analysis unless the questionnaire was not returned or the majority of responses were uninterpretable. Medication costs downloaded from practices were manually amended where they were clearly wrong on visual inspection (*e.g.* a prescription for a salbutamol inhaler with a recorded cost of over £1000). The primary analysis included all participants using imputation to predict missing costs and outcomes[27]. Data imputed (using chained equation multiple imputation methods) for the main statistical analysis were used for the economic evaluation[13 28 29]. To facilitate convergence of the imputation model, costs were imputed using aggregated cost categories (medications, pharmacy reviews, secondary care, primary care, social care and other types of care) rather than at the level of individual resource-use items.

Economic analyses

Statistical analyses were conducted using Stata 14.2[30]. All analyses were by 'intention to treat' (comparing the two groups as randomised and including all patients in the primary analysis). A glossary of economic terms is given in Appendix 2, and more details may be found in Morris *et al*[31].

A cost–utility analysis was conducted from the NHS/PSS perspective corresponding to the NICE reference case[26]. The incremental mean difference in QALYs between the two arms of the trial and 95% confidence intervals were derived. Overall mean NHS/PSS costs, and standard errors for both arms of

For peer review only - http://bmjopen.bmj.com/site/about/guidelines.xhtml

the trial were calculated. The incremental mean difference in total costs between the two arms of the trial and 95% confidence intervals were estimated.

Cost and QALY data were combined to calculate an incremental cost-effectiveness ratio (ICER) and net monetary benefit (NMB) statistic[32] from the NHS/PSS perspective. In the primary analysis we estimated whether the 3D approach was cost-effective at the established NICE thresholds of £20,000 and £30,000 per QALY gained. The probability that the 3D approach was cost-effective at various societal 'willingness to pay for a QALY' thresholds was depicted using a cost-effectiveness acceptability curve (CEAC). All measures of cost-effectiveness (ICER, CEAC and NMB) and confidence intervals were derived parametrically using the output of seemingly-unrelated regression analysis to account for the correlation between costs and outcomes, and controlling for baseline imbalance in utility for the QALY equation[33]. Clustering within GP practices was accounted for by including the randomisation variables in the regression.

As the broader societal perspective also has relevance, the costs of each component of the intervention were estimated separately from the NHS/PSS perspective (i.e. including all resources supplied by the healthcare provider), the patient/carer perspective (i.e. including all costs borne by the participants themselves) and the societal productivity perspective (i.e. taking into account time off work) and related to changes in a range of secondary outcomes in a cost–consequences analysis[34]. Consequences included QALYs accrued by both participants and carers, and deaths. The cost-consequences analysis was based on available cases, which differed in number for each type of healthcare resource or outcome; an available case was defined as an individual having complete data for each relevant timepoint. Linear regression output was used to derive confidence intervals parametrically, accounting for clustering within practices.

Sensitivity analyses

One-way sensitivity analyses were used to judge the potential impact of sources of uncertainty. This included an analysis excluding participants who died to assess the impact of the imbalance in deaths between arms, and an analysis without discounting either costs or outcomes (i.e. a discount rate of zero) to assess the impact of the discount rate. A complete-case analysis was also conducted to assess the impact of the imputation process; a complete case was defined as a participant for whom full resource-use data and full outcome data were available.

RESULTS

For peer review only - http://bmjopen.bmj.com/site/about/guidelines.xhtml

The results are reported according to the specifications of the Consolidated Health Economic Evaluation Reporting Standards (CHEERS) statement[35]. Baseline characteristics of the practices and patients are given in Table 1, and the flow of patients through the study is illustrated in Figure 1[13].

Missing data

16 practices (797 participants) were randomised to be offered the 3D approach, while 17 practices (749 participants) were randomised to receive usual care. Missing data occurred for a number of reasons, including withdrawal from the trial, or leaving the practice. 12 participants (1.5%) in the 3D arm and six (0.8%) in the control arm had no information on secondary care use because it was not possible to locate their medical records (p=0.2). Practice downloads of medication and investigation data failed for 18 participants (2.3%) in the intervention arm and eight (1.1%) in the usual care arm (p=0.07), while 19 (2.4%) and 10 (1.3%) 3D and usual care participants respectively were missing consultation data from practice downloads (p=0.13). Not all participants returned all questionnaires at all timepoints. 165 participants (20.7%) in the intervention arm and 125 (16.7%) in the control arm did not return a questionnaire at one or more follow-up points (p=0.04). Not all those who did return questionnaires completed the resource-use questions; in total, 181 (22.7%) in the intervention arm and 146 (19.5%) in the control arm were missing resource-use data from questionnaires at one or more follow-up points (p=0.12). Complete datasets were available for 1191 participants in total (599 (75.2%) in the 3D arm and 592 (79%) in the control arm, p=0.07). Participants with missing data were in a significantly poorer health state at baseline (mean (95%CI) EQ-5D-5L score: 0.453 (0.422 to 0.485)) than participants with full datasets (mean (95%CI) EQ-5D-5L score: 0.589 (0.574 to 0.605)).

Primary analysis

Outcomes and resource use

The primary analysis using imputed data showed that participants in the intervention arm gained a mean of 0.007 (95%CI: -0.009 to 0.023) additional QALYs over 15 months compared with participants in the usual care arm. Total costs per patient from the NHS/PSS perspective were £126 (95%CI: -£739 to £991) higher in the intervention arm than in the usual care arm. Wide variability was observed in both costs and outcomes, with both confidence intervals overlapping zero. Disaggregated resource-use data are in appendix 3.

Cost-effectiveness of 3D

Cost-effectiveness statistics from the NHS/PSS perspective are given in Table 2. The ICER was £18,499, and the net monetary benefit at a societal willingness-to-pay value of £20,000 was £10 (95%CI: -£956 to £977). At this willingness-to-pay value, the probability that the 3D approach is cost-effective was 0.508, while at £30,000, the probability of cost-effectiveness was 0.558. A cost-effectiveness

For peer review only - http://bmjopen.bmj.com/site/about/guidelines.xhtml

acceptability curve depicting the probability of cost-effectiveness at a range of willingness-to-pay values is given in Figure 2.

Sensitivity analyses

Results from an analysis restricted to complete cases only are given in Table 3. The complete-case analysis suggested that the 3D approach was associated with both lower costs and better outcomes, with a probability of cost-effectiveness of 0.705 at a willingness-to-pay threshold of £20,000. A sensitivity analysis excluding participants who died suggested that the probability of cost-effectiveness of the 3D approach at £20,000 was 0.561. A further sensitivity analysis using undiscounted costs and outcomes did not suggest that the discount rate affected the conclusions.

Cost–consequences analysis

Costs and selected outcomes (on an available case basis) are presented in Table 4 from the primary perspective of the NHS/PSS and the secondary perspective of the patient/carer themselves alongside an estimate of the societal loss of productivity.

Costs from all perspectives were very similar between arms and no cost group differed significantly (other than those associated with the intervention itself). Other than for daycase/outpatient care, emergency care and medications, costs to the NHS were higher in the intervention arm than in the usual care arm, while social services usage was higher in the usual care arm. Overall costs (based on available data) from the NHS/PSS perspective were slightly higher in the usual care arm although the difference was consistent with a chance finding.

Costs borne by patients and carers were higher overall in the intervention arm, although the medication costs (both prescription charges and over-the-counter remedies) were slightly lower; no patient cost group exhibited a statistically significant difference. The societal cost of productivity losses was similar in the two arms (and statistically consistent with chance), although slightly higher in the 3D approach arm.

QALYs (adjusted for baseline utility scores) were slightly higher for patients and lower for carers in the intervention arm than in the usual care arm; however, the difference was consistent with chance. Although there was a higher number of deaths in the intervention arm, the difference was not statistically significant.

DISCUSSION

For peer review only - http://bmjopen.bmj.com/site/about/guidelines.xhtml

Principal findings

No significant difference was observed between participants offered the 3D intervention and those receiving usual care only for overall costs, resource use of any category, or QALY outcomes. The participants were substantial users of healthcare, with inpatient hospital care and medications both high contributors to overall costs. A small improvement in outcome (representing an additional 2.6 days of the best imaginable health over 15 months) coupled with a small increase in costs between arms led to weak evidence for the cost-effectiveness of the 3D approach in the primary analysis from the NHS/PSS perspective. The net monetary benefit was small, but positive, indicating that society is willing to pay for the benefits that can be achieved.

Strengths and limitations

This economic evaluation was conducted alongside a large and rigorous RCT. Meticulous data collection practices allowed individual patient data to be measured for all the key cost drivers, a particularly challenging task for a patient group that has high healthcare usage. The study contributes to the body of evidence supporting the care of patients with multimorbidity. Patterns of missing resource-use data were similar between arms, and high questionnaire return rates were achieved[36]; however, the recall period for resource-use was quite long at 9 months, which may have led to patients omitting to include some resources. Medication costs were based on prescriptions issued by the GP, and it is not certain that all scripts were filled by the participant; the medication costs may, therefore, be overestimated. Although the medication data were checked carefully, the volume of medications prescribed to the 3D population rendered it infeasible to check every entry and some errors may have persisted.

Use of care homes, which can be a significant contributor to costs of social care, was not included in the economic evaluation. The funding of care homes within the UK is complex, with patients often paying considerable amounts themselves. However, only 13 participants reported living in a care home and this did not differ by arm. The follow-up period of the 3D trial was 15 months, and longer-term outcomes are unknown; a follow-up period of 2–3 years might have allowed an effect on quality of life or healthcare resource use to be observed. Use of simple mean imputation methods for estimating missing information (such as the cost of a bus fare) in the questionnaire data will have reduced standard errors and underestimated the uncertainty around these costs.

Comparison with other studies

Comparison with other studies is hampered by the variety of definitions used for multimorbidity, and by the heterogeneity in study designs[11]. Evidence concerning the cost-effectiveness of interventions designed to manage patients with multimorbidity is limited; only two of the RCTs identified in the review by Smith *et al* undertook cost-effectiveness analyses[7]. Katon *et al* reported that implementation of a collaborative care intervention for patients with depression alongside either diabetes or coronary heart

For peer review only - http://bmjopen.bmj.com/site/about/guidelines.xhtml
For peer review only

disease was justified on cost-effectiveness grounds[37]. However, the authors estimated QALYs based on clinical outcomes rather than by direct measurement, and there was substantial uncertainty around both costs and outcomes. Barley *et al* found that a nurse-led patient-centred intervention was cost-effective up to a willingness-to-pay threshold of approximately £3000 per QALY; however, the study was only a pilot RCT, and multimorbidity was defined to include patients with coronary heart disease and depression only[38]. In a feasibility trial, Mercer *et al* found that a whole-system intervention designed for patients with two or more chronic conditions in deprived areas may be considered cost-effective[39]. There is a clear need for further well conducted studies on the cost-effectiveness of interventions for multimorbid patients.

Implications of study

This is the largest trial of an intervention for multimorbidity ever undertaken; however, there remains substantial uncertainty surrounding the results. The impact of organisational changes in delivery of primary health care on patients' quality of life is usually very small and the costs are also usually low, but skewed by a small number of patients with high costs in secondary care often unrelated to the intervention. This leads to wide confidence intervals that cross zero (i.e. substantial uncertainty) for estimates of cost-effectiveness and illustrates the difficulty of obtaining precise estimates for the economic outcomes of this type of intervention even in large trials.

The 3D participants had a mean(sd) utility of 0.558(0.287) at entry to the study, which compares poorly to a UK population mean of 0.779 for ages 65-74[40]. Participants had a small positive increase in QALYs in the intervention arm, while carers had a small decrease in QALYs compared with the usual care arm; it is possible that an analysis taking carer outcomes into account might reach a different conclusion. The small contribution to overall costs made by productivity losses is consistent with the predominantly retired study population; at baseline, over 65% of participants described themselves as 'fully retired from work'.

Anticipated reductions in appointment attendances were not achieved, partly because some patients attended 3D reviews in addition to, rather than instead of, their single-condition reviews. Similarly, although cost savings could have been achieved through a reduction in prescriptions issued, this was not observed in the trial; although the brief was to seek to simplify patients' medication regimes, the embedded process evaluation[14] suggested that pharmacists appeared to concentrate on adherence to guidelines rather than whether the patient might benefit from stopping some medications. There was substantial variation between practices in the extent to which they implemented the variation and this may had contributed to variation in costs in the trial arm. Three practices barely implemented the intervention at all because of practice or staffing problems unconnected with the intervention, and this will have diluted the potential effects of the intervention overall. It is possible that the cost-effectiveness of the intervention could be increased by focusing more on patients with the greatest needs, by more attention to de-

For peer review only - http://bmjopen.bmj.com/site/about/guidelines.xhtml

prescribing, and by ending unnecessary additional disease-specific reviews as the 3D model became normalised.

Future work

The EQ-5D instrument was used because it is recommended by NICE in the UK[26], and there was little evidence on alternatives at the trial outset. However, it is difficult to pick up small changes in health-related quality of life arising from service reconfigurations, and it may be that a measure capturing outcomes beyond health would be more appropriate. In future work, researchers should consider including alternative economic outcome measures alongside the EQ-5D; for example, the ICECAP suite of capability wellbeing instruments[41]. The effect of the intervention on carers is being considered further, and it would be interesting to explore the reasons for the errors observed in the medication records downloaded from GP practice systems.

CONCLUSIONS

The evidence for the cost-effectiveness of the 3D intervention is equivocal; the results suggest that there is just over a 50% chance of cost-effectiveness at the established threshold of £20,000 per QALY from the NHS/PSS perspective. The small differences in costs and outcomes are consistent with chance, and the uncertainty is substantial; therefore, the results should be interpreted with caution. Given the equivocal nature of the cost-effectiveness results, implementation of the intervention cannot be recommended on economic grounds alone.

For peer review only

For peer review only - http://bmjopen.bmj.com/site/about/guidelines.xhtml

Table 1. Baseline characteristics of practices and patients.

	Usual care (n=17 practices, 749 patients)	Intervention (n=16 practices, 797 patients)
**Patients**		
Age: mean (SD)	70·7 (11·4)	71·0 (11·6)
Female: no. (%)	377 (50%)	406 (51%)
White ethnicity: no./total no. (%)	729/739 (99%)	775/780 (99%)
Fully retired from work: no./total no. (%)	512/721 (71%)	525/759 (69%)
No. of long term conditions from QOF: median (IQR)	3·0 (3·0, 3·0)	3·0 (3·0, 3·0)
No. of self-reported conditions: median (IQR), n	7·0 (5·0, 10·0), 748	7·0 (5·0, 9·0), 795
Long term conditions.[b] No. (%) with:		
Cardiovascular disease or chronic kidney disease[c]	698 (93%)	747 (94%)
Stroke or Transient Ischemic Attack	241 (32%)	286 (36%)
Diabetes	401 (54%)	411 (52%)
Chronic Obstructive Pulmonary Disease or Asthma	382 (51%)	388 (49%)
Epilepsy	35 (5%)	41 (5%)
Atrial fibrillation	249 (33%)	281 (35%)
Serious mental illness[d]	37 (5%)	29 (4%)
Depression	283 (38%)	276 (35%)
Dementia	27 (4%)	33 (4%)
Learning disability	7 (1%)	7 (1%)
Rheumatoid arthritis	55 (7%)	48 (6%)
EQ-5D-5L score: mean (SD), n	0·542 (0·292), 747	0·574 (0·282), 795

For peer review only

Table 2. Cost-effectiveness of the 3D approach from an NHS and personal social services perspective

	**Usual care Mean (SE) N=749**	**Intervention Mean (SE) N=797**	**Incremental difference (95% CI)**
**Costs (£)**			
Unadjusted costs from the NHS/PSS perspective	6032 (362)	6124 (317)	
Adjusted costs from the NHS/PSS perspective	6014 (343)	6140 (333)	126 (-739 to 991)
**Outcomes**			
Unadjusted QALYs over 15 months of follow-up	0.651 (0.013)	0.691 (0.012)	
Adjusted QALYs over 15 months of follow-up	0.668 (0.006)	0.675 (0.006)	0.007 (-0.009 to 0.023)
**Cost-effectiveness statistics**			
ICER: £18499			
Net monetary benefit at £20,000 (95%CI): £10 (-956 to 977)			
Net monetary benefit at £30,000 (95%CI): £78 (-974 to 1130)			

ICER, incremental cost-effectiveness ratio; CI, confidence interval; PSS, personal social services; QALY, quality-adjusted life year; SE, standard error. Cost estimates were adjusted for randomisation variables; QALY estimates were adjusted for randomisation variables and baseline utility.

For peer review only - http://bmjopen.bmj.com/site/about/guidelines.xhtml

Table 3. Sensitivity analysis: cost-effectiveness of the 3D approach from an NHS/PSS perspective based on complete cases only

	**Usual care Mean (SE) N=592 complete cases**	**Intervention Mean (SE) N=599 complete cases**	**Incremental difference (95%CI)**
Costs (£)			
Unadjusted costs from the NHS/PSS perspective	4916 (290)	4757 (222)	
Adjusted costs from the NHS/PSS perspective	4905 (258)	4768 (256)	-137 (-852 to 577)
Outcomes			
Unadjusted QALYs over 15 months of follow-up	0.698 (0.014)	0.750 (0.013)	
Adjusted QALYs over 15 months of follow-up	0.722 (0.005)	0.726 (0.005)	0.004 (-0.010 to 0.019)
Cost-effectiveness statistics			
ICER: Intervention dominates			
Net monetary benefit at £20,000: £222 (-584 to 1028)			

ICER, incremental cost-effectiveness ratio; CI, confidence interval; PSS, personal social services; QALY, quality-adjusted life year; SE, standard error. Cost estimates were adjusted for randomisation variables; QALY estimates were adjusted for randomisation variables and baseline utility.

For peer review only - http://bmjopen.bmj.com/site/about/guidelines.xhtml

Table 4. Costs and consequences of the 3D approach and usual care

**Costs and outcomes**	**Usual care**	***N***	**Intervention**	***N***	**Difference (95% CI)**
**Mean costs from the NHS perspective (£)**					
Practice-based consultations	627	754	726	715	99 (-7 to 205)
Practice-based investigations	45	755	61	717	15 (-6 to 37)
Community-based healthcare	160	615	167	601	7 (-35 to 49)
Inpatient stays	1867	766	1920	722	52 (-470 to 574)
Outpatient visits and day cases	614	766	613	722	-1 (-168 to 167)
Accident and emergency visits	102	766	99	722	-3 (-24 to 19)
Ambulance trips to hospital	131	615	141	601	10 (-56 to 77)
Prescribed medications	1230	755	1221	717	-8 (-220 to 203)
Pharmacy reviews	0	766	8	722	8 (7 to 9)
Intervention set up	0	797	4	749	4 (3 to 5)
Social services	559	615	403	601	-156 (-476 to 164)
All NHS/PSS	4929	609	4746	598	-183 (-923 to 556)
**Mean costs from the patient/carer perspective (£)**					
Prescription charges	5	755	4	717	-2 (-6 to 2)
Travel to GP practice	24	749	34	711	10 (-4 to 24)
Over-the-counter medications	39	615	35	601	-3 (-16 to 9)
Private healthcare	93	615	122	601	29 (-40 to 97)
All patient/carer	162	608	195	597	33 (-35 to 101)
**Mean societal productivity loss (£)**					
Productivity loss	122	608	161	597	39 (-47 to 125)
**Outcomes**					
QALYs (patient)	0.693	665	0.695	647	0.003 (-0.013 to 0.019)
QALYs (carer)	0.943	41	0.920	50	-0.024 (-0.064 to 0.017)
Deaths	32	749	46	797	p=0.18 (χ^2 test)

CI, confidence interval; QALY, quality-adjusted life year; PSS, personal social services. All costs and consequences are based on available data; the totals from each perspective are not, therefore, equal to the sum of the components. CI were calculated using standard errors from standard linear regressions adjusted for cluster at the level of the practice. QALYs were adjusted for baseline utility scores.

For peer review only - http://bmjopen.bmj.com/site/about/guidelines.xhtml

References

1. National Institute for Health and Care Excellence. Multimorbidity: clinical assessment and management 2016 [Available from: https://www.nice.org.uk/guidance/ng56.
2. Peters M, Kelly L, Potter CM, et al. Quality of life and burden of morbidity in primary care users with multimorbidity. *Patient Related Outcome Measures* 2018;9:103.
3. Li J, Green M, Kearns B, et al. Patterns of multimorbidity and their association with health outcomes within Yorkshire, England: baseline results from the Yorkshire Health Study. *BMC public health* 2016;16(1):649.
4. Marengoni A, Angleman S, Melis R, et al. Aging with multimorbidity: a systematic review of the literature. *Ageing research reviews* 2011;10(4):430-39.
5. Kasteridis P, Street A, Dolman M, et al. The importance of multimorbidity in explaining utilisation and costs across health and social care settings: evidence from South Somersets Symphony Project, 2014.
6. Wang L, Si L, Cocker F, et al. A Systematic Review of Cost-of-Illness Studies of Multimorbidity. *Applied health economics and health policy* 2017:1-15.
7. Smith SM, Wallace E, O'Dowd T, et al. Interventions for improving outcomes in patients with multimorbidity in primary care and community settings. *Cochrane Database of Systematic Reviews* 2016(3) doi: 10.1002/14651858.CD006560.pub3
8. Navickas R, Petric V-K, Feigl AB, et al. Multimorbidity: What do we know? What should we do? *Journal of comorbidity* 2016;6(1):4.
9. McPhail SM. Multimorbidity in chronic disease: impact on health care resources and costs. *Risk management and healthcare policy* 2016;9:143.
10. Hopman P, De Bruin SR, Forjaz MJ, et al. Effectiveness of comprehensive care programs for patients with multiple chronic conditions or frailty: a systematic literature review. *Health Policy* 2016;120(7):818-32.
11. Xu X, Mishra GD, Jones M. Evidence on multimorbidity from definition to intervention: an overview of systematic reviews. *Ageing research reviews* 2017;37:53-68.
12. Stewart M. Towards a global definition of patient centred care: the patient should be the judge of patient centred care: British Medical Journal Publishing Group, 2001.
13. Salisbury C, Man M-S, Bower P, et al. Management of multimorbidity using a patient-centred care model: a pragmatic cluster-randomised trial of the 3D approach. *The Lancet* 2018;392(10141):41-50.
14. Salisbury C, Man M-S, Chaplin K, et al. A patient-centred intervention to improve the management of multimorbidity in general practice: the 3D RCT. 2019;7:5. doi: 10.3310/hsdr07050
15. Man M-S, Chaplin K, Mann C, et al. Improving the management of multimorbidity in general practice: protocol of a cluster randomised controlled trial (The 3D Study). *BMJ open* 2016;6(4):e011261.
16. NHS Employers. Quality and outcomes framework [Available from: http://www.nhsemployers.org/your-workforce/primary-care-contacts/general-medical-services/quality-and-outcomes-framework accessed August 2018.
17. Herdman M, Gudex C, Lloyd A, et al. Development and preliminary testing of the new five-level version of EQ-5D (EQ-5D-5L). *Quality of life research* 2011;20(10):1727-36.
18. Van Hout B, Janssen M, Feng Y-S, et al. Interim scoring for the EQ-5D-5L: mapping the EQ-5D-5L to EQ-5D-3L value sets. *Value in Health* 2012;15(5):708-15.
19. National Institute for Health and Care Excellence. Position statement on use of the EQ-5D-5L valuation set 2017 [Available from: https://www.nice.org.uk/Media/Default/About/what-we-do/NICE-guidance/NICE-technology-appraisal-guidance/eq5d5l_nice_position_statement.pdf.

For peer review only - http://bmjopen.bmj.com/site/about/guidelines.xhtml

20. Curtis L, Burns A. Unit Costs of Health and Social Care 2016. University of Kent, Canterbury: Personal Social Services Research Unit 2016.
21. British National Formulary, March-September 2016. Basingstoke: Pharmaceutical Press/BMJ Group 2016.
22. Department of Health. NHS reference costs 2015 to 2016 2016 [Available from: https://www.gov.uk/government/publications/nhs-reference-costs-2015-to-2016.
23. Office for National Statistics. Annual Survey of Hours and Earnings: 2016 2016 [Available from: https://www.ons.gov.uk/employmentandlabourmarket/peopleinwork/earningsandworkinghours/datasets/agegroupashetable6.
24. HM Revenue and Customs. Travel - mileage and fuel rates and allowances Updated 6 April 2017 [Available from: https://www.gov.uk/government/publications/rates-and-allowances-travel-mileage-and-fuel-allowances/travel-mileage-and-fuel-rates-and-allowances.
25. Bank of England. UK inflation calculator [Available from: http://www.bankofengland.co.uk/education/Pages/resources/inflationtools/calculator/default.aspx.
26. NICE. Guide to the methods of technology appraisal 2013 2013 [Available from: https://www.nice.org.uk/process/pmg9/chapter/1-foreword.
27. Sterne JA, White IR, Carlin JB, et al. Multiple imputation for missing data in epidemiological and clinical research: potential and pitfalls. *Bmj* 2009;338:b2393.
28. Gomes M, Diaz-Ordaz K, Grieve R, et al. Multiple imputation methods for handling missing data in cost-effectiveness analyses that use data from hierarchical studies: an application to cluster randomized trials. *Med Decis Making* 2013;33(8):1051-63.
29. Díaz-Ordaz K, Kenward MG, Grieve R. Handling missing values in cost effectiveness analyses that use data from cluster randomized trials. *Journal of the Royal Statistical Society: Series A (Statistics in Society)* 2014;177(2):457-74. doi: 10.1111/rssa.12016
30. Stata Statistical Software: Release 14 [program]. College Station, TX: StataCorp LP., 2015.
31. Morris S, Devlin N, Parkin D. Economic analysis in health care. Chichester: John Wiley & Sons 2007.
32. Löthgren M, Zethraeus N. Definition, interpretation and calculation of cost-effectiveness acceptability curves. *Health Econ* 2000;9(7):623-30.
33. Manca A, Hawkins N, Sculpher MJ. Estimating mean QALYs in trial-based cost-effectiveness analysis: the importance of controlling for baseline utility. *Health Econ* 2005;14(5):487-96.
34. Mauskopf JA, Paul JE, Grant DM, et al. The Role of Cost—Consequence Analysis in Healthcare Decision—Making. *Pharmacoeconomics* 1998;13(3):277-88.
35. Husereau D, Drummond M, Petrou S, et al. Consolidated health economic evaluation reporting standards (CHEERS) statement. *Cost Effectiveness and Resource Allocation* 2013;11(1):6.
36. Noble SM, Hollingworth W, Tilling K. Missing data in trial-based cost-effectiveness analysis: the current state of play. *Health economics* 2012;21(2):187-200.
37. Katon W, Russo J, Lin EH, et al. Cost-effectiveness of a multicondition collaborative care intervention: a randomized controlled trial. *Archives of general psychiatry* 2012;69(5):506-14.
38. Barley EA, Walters P, Haddad M, et al. The UPBEAT nurse-delivered personalized care intervention for people with coronary heart disease who report current chest pain and depression: a randomised controlled pilot study. *PLoS One* 2014;9(6):e98704.
39. Mercer SW, Fitzpatrick B, Guthrie B, et al. The CARE Plus study–a whole-system intervention to improve quality of life of primary care patients with multimorbidity in areas of high socioeconomic deprivation: exploratory cluster randomised controlled trial and cost-utility analysis. *BMC medicine* 2016;14(1):88.
40. Janssen B, Szende A. Population norms for the EQ-5D. Self-reported population health: An international perspective based on EQ-5D: Springer 2014:19-30.

For peer review only

41. Al-Janabi H, Flynn TN, Coast J. Development of a self-report measure of capability wellbeing for adults: the ICECAP-A. *Quality of Life Research* 2012;21(1):167-76.

Figure captions

Figure 1. Trial profile †

† Salisbury C, Man M-S, Bower P, et al. Management of multimorbidity using a patient-centred care model: a pragmatic cluster-randomised trial of the 3D approach. *The Lancet* 2018;392(10141):41-50

Figure 2. Cost-effectiveness acceptability curve from the NHS/PSS perspective.†

† Salisbury C, Man M-S, Chaplin K, Mann C, Bower P, Brookes S, et al. A patient-centred intervention to improve the management of multimorbidity in general practice: the 3D RCT. Health Serv Deliv Res 2019;7(5)

For peer review only

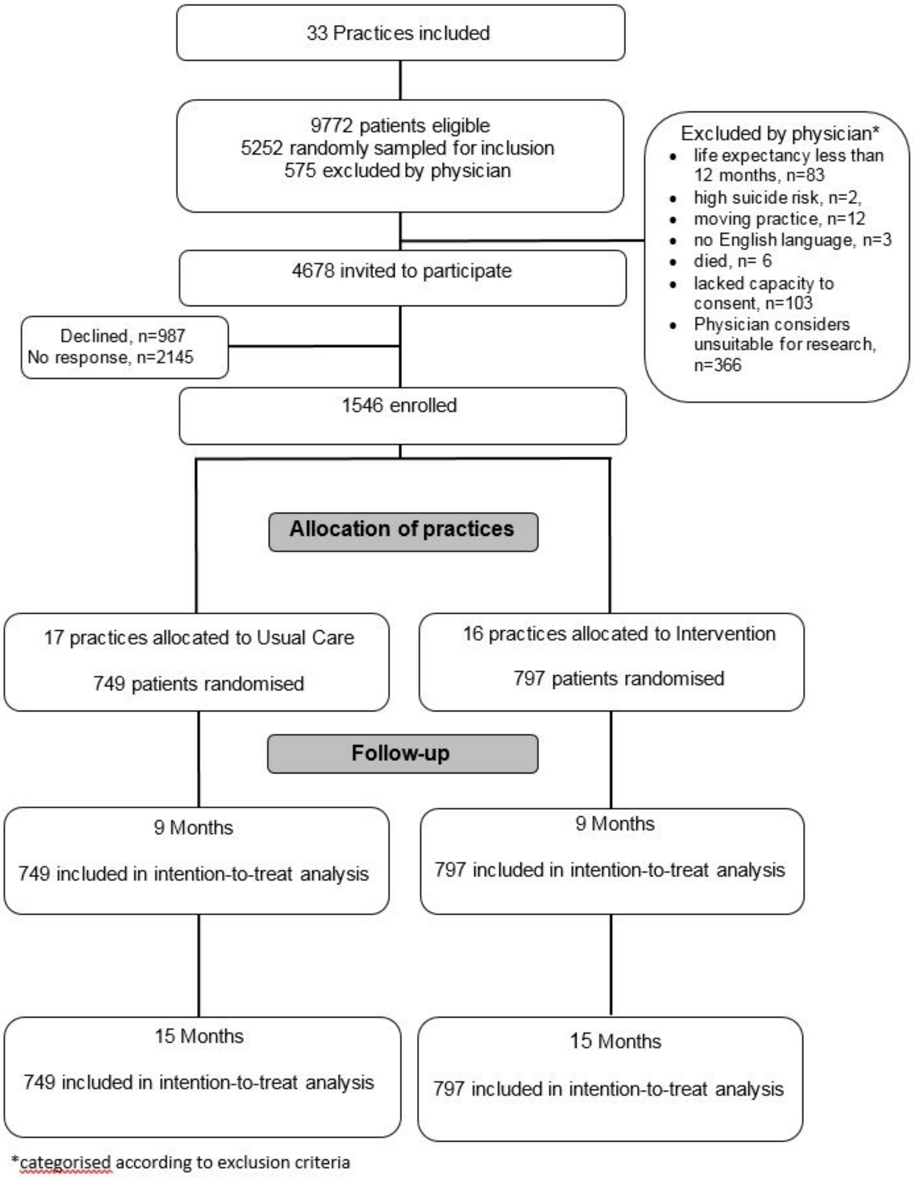

Figure 1. Trial profile †
† Salisbury C, Man M-S, Bower P, et al. Management of multimorbidity using a patient-centred care model: a pragmatic cluster-randomised trial of the 3D approach. The Lancet 2018;392(10141):41-50

190x240mm (96 x 96 DPI)

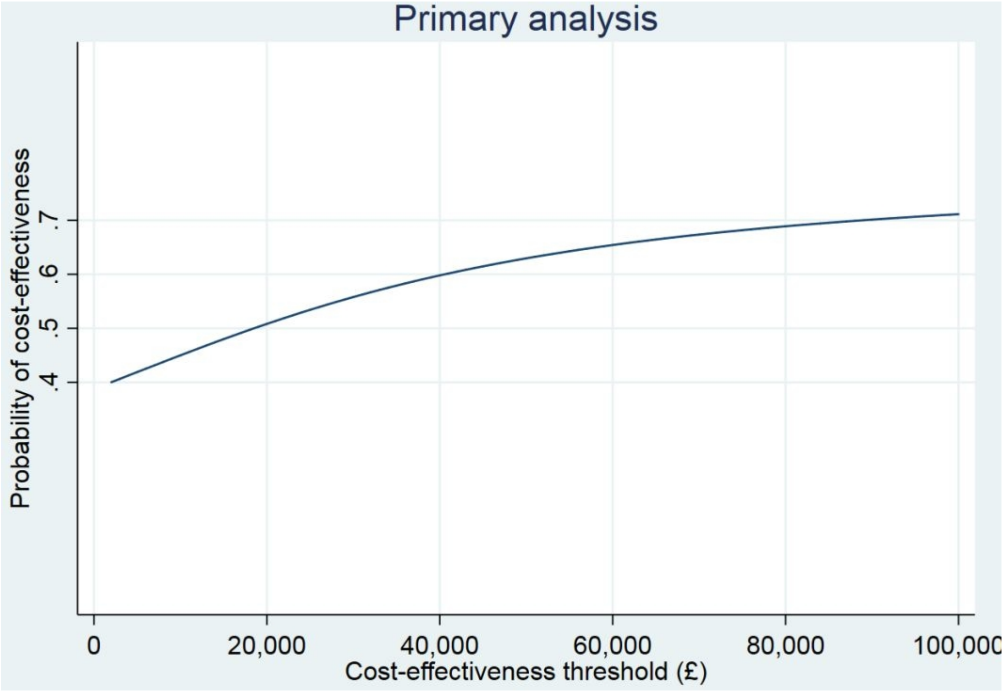

Figure 2. Cost-effectiveness acceptability curve from the NHS/PSS perspective.†† Salisbury C, Man M-S, Chaplin K, Mann C, Bower P, Brookes S, et al. A patient-centred intervention to improve the management of multimorbidity in general practice: the 3D RCT. Health Serv Deliv Res 2019;7(5)

90x61mm (300 x 300 DPI)

Appendix 1

Table 1 Unit costs

Service		Unit cost (£)	Notes	Source
**Social services**				
Social home care provided for social services		12	Independent sector home care provided for social services, face-to-face, based on 30 minute visit	1
Daycare		61	Per client attendance	
Social worker		55	Based on 1 hour appointment	
Meals on wheels		7.03		2
**Prescription charges***				
Prescription charge per item		8.40		3
Prescription charge 3mo prepayment certificate		29.10		
Prescription charge 12mo prepayment certificate		104		
**GP practice services**				
GP appointment	Control arm	51.84	Per surgery consultation lasting 14.4 minutes (3D data)	1
	Intervention arm	48.60	Per surgery consultation lasting 13.5 minutes (3D data)	

For peer review only

Service		Unit cost (£)	Notes	Source
GP home visit		100	Based on consultation lasting 15 minutes and 12 minutes travel time[4], plus 5 miles travel cost (NHS travel reimbursed at 56p per mile)	
GP phone call	Control arm	41.40	Based on consultation lasting 11.5 minutes (3D data)	
	Intervention arm	32.40	Based on consultation lasting 9 minutes (3D data)	
GP email consultation		21.60	Based on 6 minute consultation[5]	
Nurse appointment	Control arm	10.85	Based on 15.1 minute appointment (3D data), inflated from 14/15 costs[6]	
	Intervention arm	14.16	Based on 19.7 minute appointment (3D data), inflated from 14/15 costs[6]	
Nurse phone call	Control arm	4.24	Based on 5.9 minute call, inflated from 14/15 costs [6]	
	Intervention arm	5.03	Based on 7 minute call, inflated from 14/15 costs[6]	
Nurse home visit		22.21	Based on 15 minute visit, 12 minutes travelling, 5 miles travel costs (NHS travel reimbursed at 56p per mile)	
HCA face-to-face appointment	Control arm	3.45	Based on 9.4 minute appointment (3D data), band** 2 nurse	
	Intervention arm	3.59	Based on 9.8 minute appointment (3D data), band 2 nurse	
HCA phone call (control arm)		5.87	Based on 16 minute call (3D data), band 2 nurse	

2

Service	Unit cost (£)	Notes	Source
HCA home visit	12.70	Based on 15 minutes appointment, 12 minutes travelling, 5 miles travel costs, band 2 nurse (NHS travel reimbursed at 56p per mile)	
**Community services**			
Occupational Therapy visit	16	Based on band 5 occupational therapist, 30 minute appointment	[1]
Occupational Therapy home visit	25.20	Based on band 5 occupational therapist, 30 minute appointment, 12 minutes travelling, 5 miles travel costs (NHS travel reimbursed at 56p per mile)	
Occupational Therapy phone call	5.33	Based on band 5 occupational therapist, 10 minute call	
Speech and Language Therapy (SALT) visit	16	Based on band 5 SALT, 30 minute appointment	
Speech and Language Therapy home visit	25.20	Based on band 5 SALT, 30 minute appointment, 12 minutes travelling, 5 miles travel costs (NHS travel reimbursed at 56p per mile)	
Speech and Language Therapy telephone call	5.33	Based on band 5 SALT, 10 minute call	

For peer review only - http://bmjopen.bmj.com/site/about/guidelines.xhtml

Service	Unit cost (£)	Notes	Source
Physiotherapy visit	16	Based on band 5 physiotherapist, 30 minute appointment	
Physiotherapy home visit	25.20	Based on band 5 physiotherapist, 30 minute appointment, 12 minutes travelling, 5 miles travel costs (NHS travel reimbursed at 56p per mile)	
Physiotherapy phone call	5.33	Based on band 5 physiotherapist, 10 minute call	
Chiropody / Podiatry visit	16	Based on band 5 podiatrist, 30 minute appointment	
Chiropody / Podiatry home visit	25.20	Based on band 5 podiatrist, 30 minute appointment, 12 minutes travelling, 5 miles travel costs (NHS travel reimbursed at 56p per mile)	
Chiropody / Podiatry phone call	5.33	Based on band 5 podiatrist, 10 minute call	
Community / District Nurse /Mental health nurse visit	22	Based on band 6 district nurse, 30 minute appointment	
Community / District Nurse /Mental health nurse home visit	46.8	Based on band 6 district nurse, 1 hour appointment including travel time, 5 miles travel costs (NHS travel reimbursed at 56p per mile)	
Community / District Nurse/ Mental health nurse phone call	7.33	Based on band 6 district nurse, 10 minute phone call	

4

For peer review only

For peer review only

Service	Unit cost (£)	Notes	Source
NHS Counselling/Cognitive Behavioural Therapy visit	21	Based on band 6 counsellor, 30 minute appointment	
NHS Counselling/Cognitive Behavioural Therapy home visit	32.20	Based on band 6 counsellor, 30 minutes appointment, 12 minutes travelling, 5 miles travel costs (NHS travel reimbursed at 56p per mile)	
NHS Counselling/Cognitive Behavioural Therapy phone call	7	Based on band 6 counsellor, 10 minute call	
NHS 111 phone call	8.06	Maximum call cost of £7.80 in May 2013	[7]
NHS walk-in centre visit	41.74	Weighted mean of type 4 non-admitted, emergency medicine per attendance	[8]
OOH service visit	82.32	Average OOH hourly evening rate was £58.36 in 2005 compared with £36.75 for normal hours. Same differential applied to current GP in hours costs.	[9,10]
OOH service home visit	158.80		
OOH service phone call	65.74		
**Ambulance services**			
Paramedic at home not involving a hospital visit	184	Ambulance services, see, treat and refer	[1]
Ambulance to hospital	238	See and treat and convey (including carbon 39 kgCO2e)	[1]
**Investigations (in outpatients)**			

5

Service	Unit cost (£)	Notes	Source
MRI	155.64		8
DEXA	71		
CT scan	104.88		
ECG	72		
Ultrasound	58.47		
Fluoroscopy	147.68		
**Investigations (directly accessed)**			
Cytology	16.88	Directly accessed pathology services	8
Histopathology and histology	30.77	Directly accessed pathology services	
Clinical Biochemistry	1.18	Directly accessed pathology services	
Haematology	3.10	Directly accessed pathology services	
Immunology	6.42	Directly accessed pathology services	
Microbiology	7.63	Directly accessed pathology services	
Other	3.13	Directly accessed pathology services	
MRI scan	147.25	Diagnostic imaging, directly accessed, weighted mean	
CT scan	107.52	Diagnostic imaging, directly accessed, weighted mean	
Ultrasound scan	52.82	Diagnostic imaging, directly accessed, weighted mean	
Bone scan	210.11	Nuclear medicine, directly accessed, weighted mean	

For peer review only - http://bmjopen.bmj.com/site/about/guidelines.xhtml

Service	Unit cost (£)	Notes	Source
x-ray	30.26	Directly accessed diagnostic services, Plain film	
DEXA scan	68.29	Diagnostic imaging, direct access	
ECG	67.06	Diagnostic imaging, direct access, 19 years and over	
**Secondary care**			
Outpatient attendance	135.27	Weighted mean, consultant led	[8]
Day case	733.31	Weight mean of all daycases	
Elective inpatient	3749.81	Weighted mean	
Non-elective inpatient, long stay	3058.14	Weighted mean	
Non-elective inpatient, short stay	615.83	Weighted mean	
Elective excess bed day	361.67	Weighted mean	
Non-elective excess bed day	298.41	Weighted mean	
Individual HRGs		Mapped directly from Reference Costs file	
**Productivity losses**			
Median hourly wage, age 22-29	10.52	Excluding overtime, for employees on adult wages not affected by absence	[11]
Median hourly wage, age 30-39	13.57		
Median hourly wage, age 40-49	13.92		
Median hourly wage, age 50-59	13.18		

Service	Unit cost (£)	Notes	Source
Median hourly wage, age 60+	11.26		

* Where prescription charges are levied, patients can choose to pay for each individual item separately, or can purchase a pre-payment certificate to cover any number of prescriptions in a 3- or 12-month period ** NHS bands refer to payscales: for example, a recently qualified nurse will be paid on band 5, while a nurse with more experience might be paid on band 6.

OOH: out-of-hours. HCA: health care assistant. MRI: magnetic resonance imaging. DEXA: dual energy X-ray absorptiometry. ECG: electrocardiogram. CT: computerized tomography.

1. Curtis L, Burns A. Unit Costs of Health and Social Care 2016. University of Kent, Canterbury: Personal Social Services Research Unit 2016.
2. Curtis L. Unit Costs of Health and Social Care 2014. University of Kent, Canterbury: Personal Social Services Research Unit 2014.
3. Department of Health. NHS charges from April 2016 2016 [Available from: https://www.gov.uk/government/speeches/nhs-charges-from-april-2016.
4. Curtis L, Burns A. Unit Costs of Health and Social Care 2015. University of Kent, Canterbury: Personal Social Services Research Unit 2015.
5. Matheson C. Implementation of WebGP and e-consultations in Wessex GP practices. 2016
6. Bank of England. UK inflation calculator [Available from: http://www.bankofengland.co.uk/education/Pages/resources/inflationtools/calculator/default.aspx.
7. Health Committee. Written evidence from NHS Direct NHS Trust (ES 31) 2013 [Available from: https://www.publications.parliament.uk/pa/cm201314/cmselect/cmhealth/171/171vw25.htm.
8. Department of Health. NHS reference costs 2015 to 2016 2016 [Available from: https://www.gov.uk/government/publications/nhs-reference-costs-2015-to-2016.
9. O'Dowd A. Cost of out of hours care was 22% higher than predicted in England. *BMJ: British Medical Journal* 2006;332(7550):1113.
10. Curtis L, Netten A. Unit costs of health and social care: University of Kent; 2005 [
11. Office for National Statistics. Annual Survey of Hours and Earnings: 2016 2016 [Available from: https://www.ons.gov.uk/employmentandlabourmarket/peopleinwork/earningsandworkinghours/datasets/agegroupashetable6.

For peer review only - http://bmjopen.bmj.com/site/about/guidelines.xhtml

Appendix 2. **Glossary of economic terms.**

Economic evaluation

Economic evaluation refers to an analysis comparing both costs and outcomes (or benefits) to assess the value for money that an intervention offers.

Cost-consequences analysis

A cost-consequences analysis (CCA) is a type of economic evaluation in which the costs and the outcomes are presented in disaggregated form. No attempt is made to combine costs and outcomes into a cost-effectiveness statistic, instead providing commissioners with a broad range of relevant information, and allowing the scope to apply their own priorities.

Cost-effectiveness analysis

A cost-effectiveness analysis (CEA) is a type of economic evaluation in which the outcomes (or benefits) are measured in terms of a single (usually) clinical measure. For example, a cost-effectiveness analysis might have a result expressed in terms of cost per life-year gained.

Cost-utility analysis

A cost-utility analysis (CUA) is a type of economic evaluation in which the outcome is assessed in terms of a measure of health outcome to which preference-based valuations have been attached. The most commonly used outcome is the quality-adjusted life year (QALY). CUA is a particular form of CEA.

Net monetary benefit

The net monetary benefit is a cost-effectiveness statistic combining costs and outcomes in a single measure, and taking into account the societal 'willingness-to-pay' for a unit of improvement. A positive incremental net monetary benefit indicates that society is willing to pay for the benefits that can be gained.

For peer review only

Appendix 3. Mean number of health and social care resource-use contacts over 15 months of follow-up.

Services	Usual care		Intervention	
	mean (SD)	*N*	mean (SD)	*N*
Inpatient stays	0.50 (1.05)	722	0.55 (1.09)	766
Inpatient nights	4.51 (16.6)	722	3.84 (11.94)	766
Outpatient visits	3.60 (4.00)	722	3.64 (3.73)	766
Day cases	0.15 (0.54)	722	0.14 (0.51)	766
Accident and emergency visits	0.63 (1.41)	722	0.61 (1.14)	766
Ambulance journeys	0.55 (1.83)	601	0.60 (3.50)	615
GP face-to-face consultations	7.27 (5.93)	715	8.70 (6.26)	754
GP home visits	0.64 (1.89)	715	0.79 (2.16)	754
GP phone calls	2.88 (5.00)	715	2.99 (4.53)	754
Nurse face-to-face consultations	5.40 (5.70)	715	7.93 (10.09)	754
Nurse home visits	0.06 (0.40)	715	0.09 (0.54)	754
Nurse phone calls	0.60 (1.56)	715	0.76 (1.63)	754
Healthcare assistant face-to-face consultations	2.42 (4.30)	715	3.55 (7.75)	754
Healthcare assistant GP home visits	0.00 (0.05)	715	0.08 (0.28)	754
Healthcare assistant GP phone calls	0.03 (0.17)	715	0.03 (0.17)	754
Prescribed medications	143.44 (130.57)	717	132.23 (117.36)	755
GP investigations	19.60 (16.52)	717	22.61 (16.99)	755
Pharmacist reviews		722	0.78 (0.42)	766
Occupational therapist clinic visits	0.18 (1.10)	601	0.2 (1.28)	615
Occupational therapist home visits	0.24 (1.29)	601	0.08 (0.5)	615
Occupational therapist phone calls	0.06 (0.53)	601	0.01 (0.14)	615
Speech and language therapist clinic visits	0.02 (0.33)	601	0.1 (1.03)	615
Speech and language therapist home visits	0.04 (0.69)	601	0 (0)	615
Speech and language therapist phone calls	0.00 (0.08)	601	0 (0)	615
Physiotherapist clinic visits	0.80 (2.74)	601	1.03 (2.67)	615
Physiotherapist home visits	0.16 (1.34)	601	0.15 (0.92)	615
Physiotherapist phone calls	0.00 (0.06)	601	0.01 (0.11)	615
Podiatrist clinic visits	1.26 (2.83)	601	1.19 (2.53)	615
Podiatrist home visits	0.13 (0.84)	601	0.21 (1.46)	615
Podiatrist phone calls	0.01 (0.13)	601	0.02 (0.49)	615
Community mental health nurse clinic contacts	0.27 (2.59)	601	0.08 (0.42)	615
Community mental health nurse home visits	0.21 (2.45)	601	0.35 (5.16)	615
Community mental health nurse phone calls	0.02 (0.23)	601	0.01 (0.13)	615

For peer review only

District nurse home visits	1.21 (4.08)	601	1.48 (6.54)	615
District nurse phone calls	0.03 (0.30)	601	0.05 (0.84)	615
Counsellor clinic visits	0.24 (2.34)	601	0.14 (0.99)	615
Counsellor home visits	0.01 (0.10)	601	0.01 (0.18)	615
Counsellor phone calls	0.00 (0.08)	601	0.00 (0.06)	615
NHS 111 phone calls	0.21 (0.85)	601	0.18 (0.57)	615
NHS walk-in centre visits	0.08 (0.41)	601	0.09 (0.50)	615
GP out-of-hours clinic visits	0.08 (0.34)	601	0.07 (0.28)	615
GP out-of-hours home visits	0.04 (0.33)	601	0.06 (0.56)	615
GP out-of-hours phone calls	0.02 (0.20)	601	0.03 (0.28)	615
Paramedic attendances at home	0.09 (0.43)	601	0.05 (0.29)	615
Other healthcare clinic visits	0.11 (0.88)	601	0.25 (3.24)	615
Other healthcare home visits	0.06 (0.65)	601	0.04 (0.41)	615
Other healthcare phone calls	0.01 (0.19)	601	0.01 (0.10)	615
Carer contacts	30.93 (148.26)	601	25.27 (135.78)	615
Daycare contacts	2.14 (17.53)	601	1.17 (10.50)	615
Meals on wheels	2.66 (28.32)	601	0.93 (18.98)	615
Social worker contacts	0.77 (11.35)	601	0.45 (3.98)	615

For peer review only

Reporting checklist for economic evaluation of health interventions

.

Based on the CHEERS guidelines.

Instructions to authors

Complete this checklist by entering the page numbers from your manuscript where readers will find each of the items listed below.

Your article may not currently address all the items on the checklist. Please modify your text to include the missing information. If you are certain that an item does not apply, please write "n/a" and provide a short explanation.

Upload your completed checklist as an extra file when you submit to a journal.

In your methods section, say that you used the CHEERS reporting guidelines, and cite them as:

Husereau D, Drummond M, Petrou S, Carswell C, Moher D, Greenberg D, Augustovski F, Briggs AH, Mauskopf J, Loder E. Consolidated Health Economic Evaluation Reporting Standards (CHEERS) statement.

		Reporting Item	Page Number
	#1	Identify the study as an economic evaluation or use more specific terms such as “cost-effectiveness analysis”, and describe the interventions compared.	1

For peer review only

	#2	Provide a structured summary of objectives, perspective, setting, methods (including study design and inputs), results (including base case and uncertainty analyses), and conclusions	3
Background and objectives	#3	Provide an explicit statement of the broader context for the study. Present the study question and its relevance for health policy or practice decisions	5
Target population and subgroups	#4	Describe characteristics of the base case population and subgroups analysed, including why they were chosen.	5
Setting and location	#5	State relevant aspects of the system(s) in which the decision(s) need(s) to be made.	5
Study perspective	#6	Describe the perspective of the study and relate this to the costs being evaluated.	5
Comparators	#7	Describe the interventions or strategies being compared and state why they were chosen.	5-6
Time horizon	#8	State the time horizon(s) over which costs and consequences are being evaluated and say why appropriate.	5
Discount rate	#9	Report the choice of discount rate(s) used for costs and outcomes and say why appropriate	7
Choice of health outcomes	#10	Describe what outcomes were used as the measure(s) of benefit in the evaluation and their relevance for the type of analysis performed	6

For peer review only

Meaurement of effectiveness	#11a	Single study-based estimates: Describe fully the design features of the single effectiveness study and why the single study was a sufficient source of clinical effectiveness data	5
Measurement of effectiveness	#11b	Synthesis-based estimates: Describe fully the methods used for identification of included studies and synthesis of clinical effectiveness data	n/a
Measurement and valuation of preference based outcomes	#12	If applicable, describe the population and methods used to elicit preferences for outcomes.	6
	#13a	Single study-based economic evaluation: Describe approaches used to estimate resource use associated with the alternative interventions. Describe primary or secondary research methods for valuing each resource item in terms of its unit cost. Describe any adjustments made to approximate to opportunity costs	6-7
Estimating resources and costs	#13b	Model-based economic evaluation: Describe approaches and data sources used to estimate resource use associated with model health states. Describe primary or secondary research methods for valuing each resource item in terms of its unit cost. Describe any adjustments made to approximate to opportunity costs.	n/a
Currency, price date,	#14	Report the dates of the estimated resource quantities and	7

For peer review only

and conversion		unit costs. Describe methods for adjusting estimated unit costs to the year of reported costs if necessary. Describe methods for converting costs into a common currency base and the exchange rate.	
Choice of model	#15	Describe and give reasons for the specific type of decision analytical model used. Providing a figure to show model structure is strongly recommended.	n/a
Assumptions	#16	Describe all structural or other assumptions underpinning the decision-analytical model.	n/a
Analytical methods	#17	Describe all analytical methods supporting the evaluation. This could include methods for dealing with skewed, missing, or censored data; extrapolation methods; methods for pooling data; approaches to validate or make adjustments (such as half cycle corrections) to a model; and methods for handling population heterogeneity and uncertainty.	8-9
Study parameters	#18	Report the values, ranges, references, and, if used, probability distributions for all parameters. Report reasons or sources for distributions used to represent uncertainty where appropriate. Providing a table to show the input values is strongly recommended.	n/a
Incremental costs and outcomes	#19	For each intervention, report mean values for the main categories of estimated costs and outcomes of interest, as well as mean differences between the comparator groups.	18

For peer review only

		If applicable, report incremental cost-effectiveness ratios.	
Characterising uncertainty	#20a	Single study-based economic evaluation: Describe the effects of sampling uncertainty for the estimated incremental cost and incremental effectiveness parameters, together with the impact of methodological assumptions (such as discount rate, study perspective).	10
	#20b	Model-based economic evaluation: Describe the effects on the results of uncertainty for all input parameters, and uncertainty related to the structure of the model and assumptions.	n/a
Characterising heterogeneity	#21	If applicable, report differences in costs, outcomes, or cost effectiveness that can be explained by variations between subgroups of patients with different baseline characteristics or other observed variability in effects that are not reducible by more information.	10
Study findings, limitations, generalisability, and current knowledge	#22	Summarise key study findings and describe how they support the conclusions reached. Discuss limitations and the generalisability of the findings and how the findings fit with current knowledge.	11-13
Source of funding	#23	Describe how the study was funded and the role of the funder in the identification, design, conduct, and reporting of the analysis. Describe other non-monetary sources of support	1
Conflict of interest	#24	Describe any potential for conflict of interest of study	2

For peer review only

contributors in accordance with journal policy. In the absence of a journal policy, we recommend authors comply with International Committee of Medical Journal Editors recommendations

The CHEERS checklist is distributed under the terms of the Creative Commons Attribution License CC-BY-NC. This checklist can be completed online using https://www.goodreports.org/, a tool made by the EQUATOR Network in collaboration with Penelope.ai

For peer review only

BMJ Open

Cost-effectiveness of a patient-centred approach to managing multimorbidity in primary care: a pragmatic cluster randomised controlled trial

Journal:	*BMJ Open*
Manuscript ID	bmjopen-2019-030110.R2
Article Type:	Original research
Date Submitted by the Author:	15-Nov-2019
Complete List of Authors:	Thorn, Joanna; University of Bristol, Population Health Sciences<br>Man, Mei-See; University of Bristol School of Social and Community Medicine,<br>Chaplin, Katherine ; University of Bristol Centre for Academic Primary Care, Bristol Medical School<br>Bower, Peter; University of Manchester, NPCRDC<br>Brookes, Sara; Institute of Cancer and Genomic Sciences, University of Birmingham, Cancer Research UK Clinical Trials Unit ; University of Bristol, Population Health Sciences<br>Gaunt, Daisy; University of Bristol Faculty of Medicine and Dentistry, Bristol Randomised Trials Collaboration & School of Social and Community Medicine; University of Bristol<br>Fitzpatrick, Bridie; University of Glasgow, Institute of Health and Wellbeing<br>Gardner, Caroline; University of Manchester Faculty of Biology, Medicine and Health<br>Guthrie, Bruce; University of Dundee, Population Health Sciences Division, Medical Research Institute<br>Hollinghurst, Sandra; University of Bristol, Community Based Medicine<br>Lee, Victoria; University of Manchester Faculty of Biology, Medicine and Health<br>Mercer, Stewart; University of Glasgow, Institute of Health and Wellbeing<br>Salisbury, Chris; University of Bristol, Academic Unit of Primary Health Care
**Primary Subject Heading**:	Health economics
Secondary Subject Heading:	General practice / Family practice
Keywords:	Multimorbidity, PRIMARY CARE, patient-centred care, economic evaluation

For peer review only - http://bmjopen.bmj.com/site/about/guidelines.xhtml

Cost-effectiveness of a patient-centred approach to managing multimorbidity in primary care: a pragmatic cluster randomised controlled trial

Joanna C Thorn[1], Mei-See Man[1,2], Katherine Chaplin[1], Peter Bower[3], Sara T Brookes[2], Daisy Gaunt[2], Bridie Fitzpatrick[4], Caroline Gardner[3], Bruce Guthrie[5], Sandra Hollinghurst[1], Victoria Lee[3], Stewart W Mercer[6], Chris Salisbury[1]

[1] Centre for Academic Primary Care, Population Health Sciences, Bristol Medical School, University of Bristol, Canynge Hall, 39 Whatley Road, Bristol, BS8 2PS, UK
[2] Bristol Randomised Trials Collaboration, Population Health Sciences, University of Bristol, Canynge Hall, 39 Whatley Road, Bristol, BS8 2PS, UK
[3] NIHR School for Primary Care Research, Centre for Primary Care and Health Services Research, Faculty of Biology, Medicine and Health, University of Manchester, Manchester Academic Health Science Centre, UK
[4] Institute of Health and Wellbeing, University of Glasgow, 1 Lilybank Gardens, Glasgow, G12 8RZ, UK
[5] Division of Population Health Sciences, Medical Research Institute, University of Dundee, Dundee, UK
[6] Usher Institute, University of Edinburgh, Old Medical School, Teviot Place, Edinburgh, EH8 9AG, UK

Corresponding author: Dr Joanna Thorn, Population Health Sciences, University of Bristol, 1-5 Whiteladies Road, Bristol, BS8 1NU, UK; joanna.thorn@bristol.ac.uk; Tel. +44 (0) 117 4283125

4010 words

Funding: This project was funded by the National Institute for Health Research (NIHR) Health Services and Delivery Research Programme (project number 12/130/15). This study was designed and conducted in collaboration with the Bristol Randomised Trials Collaboration (BRTC), a UKCRC Registered clinical trials unit (CTU) in receipt of NIHR CTU support funding. CS is partly supported by The NIHR Collaboration for Leadership in Applied Health Research and Care West (CLAHRC West) and by Bristol Clinical Commissioning Group.

The views and opinions expressed in this report are those of the authors and do not necessarily reflect those of the NIHR, the NHS or the Department of Health.

Keywords: Multimorbidity, primary care, economic evaluation, patient-centred care

Running title: Multimorbidity management in primary care

For peer review only - http://bmjopen.bmj.com/site/about/guidelines.xhtml

Acknowledgments: We would like to thank the patients and practices who participated in the study, the patients and carers who contributed to the advisory group and the other members of the 3D study team (Cindy Mann, Polly Duncan, Keith Moffatt, Emma Moody, Imran Rafi, Becca Robinson, Ali Heawood, Bryar Kadir, John McLeod). We would also like to thank Professor Joanna Coast and Dr Padraig Dixon for valuable guidance. This article is an abbreviated version of the final report to the funders (Salisbury C, Man M-S, Chaplin K, Mann C, Bower P, Brookes S, et al. A patient-centred intervention to improve the management of multimorbidity in general practice: the 3D RCT. *Health Serv Deliv Res* 2019;7(5)).

Author contributions

CS, PB, BG, STB, SH and SWM conceived, designed and interpreted results from the 3D trial. SH and JCT designed the economic evaluation. M-SM (trial manager), KC, BF, CG and VL contributed to local economic data collection and interpretation of results. DG (statistician) conducted the multiple imputation. JCT carried out the analysis and wrote the paper, and all authors revised it for important intellectual content.

Competing interests statement

CS reports grants from CLAHRC West and Bristol CCG outside the submitted work. No other competing interests are declared.

For peer review only

ABSTRACT

Objective Patients with multiple chronic health conditions are often managed in a disjointed fashion in primary care, with annual review clinic appointments offered separately for each condition. This study aimed to determine the cost-effectiveness of the 3D intervention, which was developed to improve the system of care.

Design Economic evaluation conducted alongside a pragmatic cluster-randomised trial.

Setting General practices in three centres in England and Scotland.

Participants 797 adults with three or more chronic conditions were randomised to the 3D intervention, while 749 participants were randomised to receive usual care.

Intervention The 3D approach: comprehensive 6-monthly GP consultations, supported by medication reviews and nurse appointments.

Primary and secondary outcome measures The primary economic evaluation assessed the cost per quality-adjusted life year (QALY) gained from the perspective of the NHS and personal social services (PSS). Costs were related to changes in a range of secondary outcomes (QALYs accrued by both participants and carers, and deaths) in a cost–consequences analysis from the perspectives of the NHS/PSS, patients/carers and productivity losses.

Results

Very small increases were found in both QALYs (adjusted mean difference 0.007 [-0.009 to 0.023]) and costs (adjusted mean difference £126 [£-739 to £991]) in the intervention arm compared with usual care after 15 months. The incremental cost-effectiveness ratio was £18,499, with a 50.8% chance of being cost-effective at a willingness-to-pay threshold of £20,000 per QALY (55.8% at £30,000 per QALY).

Conclusions

The small differences in costs and outcomes were consistent with chance, and the uncertainty was substantial; therefore, the evidence for the cost-effectiveness of the 3D approach from the NHS/PSS perspective should be considered equivocal.

For peer review only - http://bmjopen.bmj.com/site/about/guidelines.xhtml

Article summary

Strengths and limitations of this study

- This economic evaluation was conducted alongside the largest randomised controlled trial of an intervention for managing multimorbid patients in primary care.
- Data collection was meticulous and high questionnaire return rates were achieved.
- Data on the use of care homes, which may contribute significantly to social care costs, were not available to include in the economic evaluation.
- Estimates of healthcare costs for this type of intervention have wide confidence intervals, even with a substantial sample size.

Data sharing statement

Requests for data sharing should be submitted to the Professor C Salisbury (c.salisbury@bristol.ac.uk) for consideration. Access to anonymised data might be granted following review via the University of Bristol Research Data Storage Facility.

For peer review only

For peer review only - http://bmjopen.bmj.com/site/about/guidelines.xhtml

INTRODUCTION

The number of patients living with multiple chronic health conditions (multimorbidity) is increasing in developed countries as the population ages[1]. Multimorbidity is associated with poor health-related quality of life[2 3], higher use of health services and higher costs[4 5]. Multimorbidity is common for older adults (with a prevalence of up to 98%[4]), leading to a substantial economic burden[6].

Care pathways for patients with multimorbidity are often poorly co-ordinated and burdensome for patients. Each condition may be treated separately, which fails to take account of interactions between diseases[1]. There have been relatively few randomised controlled trials (RCTs) of interventions designed to address issues affecting multimorbid patients, with mixed findings leading to substantial uncertainty about the effectiveness of such interventions[7]. Evidence on the cost-effectiveness of interventions remains even more limited and the evidence that is available is inconsistent[7-9]. A recent review of comprehensive care programmes for multimorbidity found no evidence that such interventions reduce healthcare costs or primary care visits[10]. Several reviews have highlighted the need for research on new interventions, including assessment of cost-effectiveness[1 7 11]. The inconsistency in the evidence for cost-effectiveness provided a clear indication that a large, well-conducted trial with an integral economic evaluation needed to be carried out.

The '3D' intervention was developed to address the issues associated with managing patients with multimorbidity in primary care in the UK. The intervention was based on a patient-centred care model and aimed to improve continuity of care with caregivers in GP surgeries, reduce patient burden in accessing healthcare and increase patient involvement in decision-making about their care (see Box 1). Based on the seminal work of Stewart[12], the concept of patient-centred care includes a focus on the patient's individual illness experience, an integrated biopsychosocial perspective, seeking to find common ground with the patient and agreeing management plans, and enhancing a continuing relationship between the patient and doctor. We sought to apply these principles within the 3D intervention. The 3D randomised trial assessed the effectiveness of the 3D approach compared with usual care, and incorporated an embedded process evaluation. The trial did not find any differences in health-related quality of life over 15 months of follow-up, but the intervention did lead to improved patient-centred care[13 14]. Here, we report the results of the economic evaluation conducted alongside the 3D trial.

For peer review only - http://bmjopen.bmj.com/site/about/guidelines.xhtml

Box 1. The 3D model and usual care in the UK

The 3D model

3D is a name used to allude to the concept of wholeness, while also acting as a mnemonic for 'dimensions of health, depression and drugs'.

- Based on a patient centred care model.
- Practice level changes to improve continuity of care and to replace disease-focused reviews of each health condition with one six-monthly comprehensive 3D review with a named GP.
- Each 3D review consisted of three elements
  1) Nurse consultation to identify health problems most important to the patient, issues with quality of life, screening for depression, collecting health data e.g. blood pressure and information relevant to the patient's specific conditions.
  2) Pharmacist review of medication from medical records, aiming to simplify and optimise drug treatment. Pharmacists were asked to identify non-essential drugs that could be stopped and essential drugs that should be started, and to seek ways to simplify drug treatment regimes e.g. by making all doses once daily.
  3) GP reviewed data from nurse and pharmacist, and agreed a health plan with the patient, which was given to them as a printed copy.
- An interactive computerised template enabled sharing of data between the clinicians, tailored the review to the patient's specific conditions, contained automated checks, and generated printed summaries to enable sharing of information and plans with the patient.
- Strategies to encourage implementation were used, including training, monthly feedback about implementation, and financial incentives for completed reviews.

Usual care in the UK

- Review of chronic conditions is mainly carried out by nurses in primary care using disease-specific data-entry screens or templates.
- Nurses commonly specialise in particular conditions and review each disease separately.
- Chronic disease reviews mainly focus on meeting the requirements of the QOF pay-for-performance scheme.

For peer review only - http://bmjopen.bmj.com/site/about/guidelines.xhtml

METHODS

Design: The 3D trial

Full details of the study design, eligibility criteria, recruitment process and clinical effectiveness results are given elsewhere[14 15]. A favourable ethical opinion was granted by the South-West (Frenchay) National Health Service (NHS) Research Ethics Committee (14/SW/0011), the trial was prospectively registered (ISCRTN06180958), and all patients and practices gave written informed consent to participate in the study. In brief, the pragmatic 3D cluster randomised trial compared the effectiveness and cost-effectiveness of a complex intervention for multimorbidity with usual care delivered in 33 general practices in Scotland and England. The target patients were adults with multimorbidity, defined as having three or more chronic conditions from those included in the NHS Quality and Outcomes Framework[16]. Clinical staff in intervention practices attended two half-day 3D training sessions. Participants in the intervention arm were offered six-monthly holistic reviews of their health problems. Each review consisted of a nurse review appointment to collect relevant clinical information, a medication review conducted by a pharmacist and an extended appointment for the patient to discuss their conditions with a named GP. An individual health plan was negotiated with patients, detailing their own priorities for managing their conditions. The primary outcome measure for effectiveness was health-related quality of life at 15 months after randomisation, measured using the EQ-5D-5L[17].

The primary economic cost–utility analysis was conducted from the NHS and personal social services (NHS/PSS) perspective over 15 months of follow-up. A secondary analysis was conducted from the perspective of patients and carers.

Patient and public involvement

An active group of up to 14 patients and carers provided a service user perspective, contributing to refinement of the research questions, design of the intervention, design of outcome measures, analysis of qualitative data, patient newsletters, the study website and interpretation of findings. All participants were given a summary of the findings and a link to the published paper describing the main results. One of the outcome measures in the study was treatment burden and this was compared between trial arms.

Measures

Outcome measurement

The primary economic outcome measure was quality-adjusted life years (QALYs). EQ-5D-5L measurements were collected by postal questionnaires at baseline, 9 months and 15 months post-recruitment, supplemented by telephone collection for non-responders. Utility scores based on a UK population were derived from responses to the EQ-5D-5L cross-mapped to valuations obtained for the EQ-5D-3L instrument[18]. This was a change (approved by the Independent Data Monitoring Committee) to

For peer review only - http://bmjopen.bmj.com/site/about/guidelines.xhtml

the analysis originally planned, as the National Institute for Health and Care Excellence (NICE) issued a position statement recommending this approach[19] subsequent to the design of the study but prior to analysis. QALYs over the 15-month period were formed from these valuations by means of linear interpolation and an area under the curve calculation. Patients who died during follow-up were treated as if their most recent utility score was relevant until death, and set to zero immediately at death. Carers also completed the EQ-5D-5L instrument at the same timepoints as patients.

Resource-use measurement

As the trial population had multimorbidity, resource use related to any health condition experienced by the participant was considered relevant.

Staff training attendance records were kept to track resources used to deliver the training programmes for GPs, nurses and receptionists, including trainee and trainer delivery time (and preparation time), travel costs, and course materials. 3D GP appointments, nurse appointments and pharmacist reviews were captured by downloading routine GP records, supplemented by manual data capture by researchers reviewing participants' medical records at the end of the trial.

Data on medications prescribed and tests/investigations in primary care were extracted from downloads of routine GP records. Details of the number and duration of primary care consultations (other than 3D reviews) were similarly extracted. These included face-to-face, telephone and home consultations with doctors, nurses or health care assistants based in general practice. Duration details were not available for practices in Scotland. Therefore, an average duration for each type of consultation by each staff type in each arm was derived using available data (practices in England only) and applied to all relevant consultations. The research team collected NHS secondary care data from participants' GP records.

To assess travel costs, the patient's normal transport method for GP appointments and the cost (for public transport) or mileage (for private transport) were collected in the patient-reported questionnaire at baseline, along with usual practice with regards to time off work, and details of whether the participant paid prescription charges or not. NHS community care, care from social services, time off work for patients and carers to attend hospital appointments and over-the-counter medication expenditure were captured in the patient-reported questionnaires at 9 and 15 months, as were private use of treatments and therapies.

Valuation of resource use

Unit costs for NHS staff time for training and delivery of the intervention were based on the most recently available national estimates[20]. Actual expenses relating to training materials, refreshments and staff travel were recorded. Because only some GPs and a small proportion of patients in each practice

For peer review only - http://bmjopen.bmj.com/site/about/guidelines.xhtml

participated in the trial, costs were adjusted to estimate the cost of training a full practice, divided by the number of eligible patients in the practice and annualised over an estimated 5-year period of relevance. Medication costs were downloaded directly as part of the routine GP records, supplemented by estimates from the British National Formulary where necessary[21]. Patient prescription charges were subtracted from the NHS perspective medication costs. Community and primary care costs were based on recent national estimates[20].

Secondary care contacts were assigned to a healthcare resource group (HRGs, groups of events that have been judged to consume similar levels of resources) and costed based on national reference costs[22]. Productivity costs for both patients and carers were estimated based on average weekly earnings stratified by age group[23]. Mileage costs were estimated using UK government allowances[24]. Costs for over-the-counter medication and private therapies and treatments were all reported directly by patients. Unit costs used in the analyses are detailed in appendix 1[14].

All costs were reported in 2015/2016 pounds sterling, adjusted for inflation where necessary[25]. Costs and outcomes occurring during the final three months of follow-up were discounted in line with NICE guidance (currently 3.5%)[26]. Dates were not available for all types of resource use measured in the trial; in these cases, 50% of the costs incurred in the final six months of follow-up were subjected to discounting.

Data cleaning and missing costs and outcomes

Data cleaning was undertaken prior to unblinding. Questionnaires were not classed as 'missing data' for the cost analysis unless the questionnaire was not returned or the majority of responses were uninterpretable. Medication costs downloaded from practices were manually amended where they were clearly wrong on visual inspection (*e.g.* a prescription for a salbutamol inhaler with a recorded cost of over £1000). The primary analysis included all participants using imputation to predict missing costs and outcomes[27]. Data imputed (using chained equation multiple imputation methods) for the main statistical analysis were used for the economic evaluation[13 14 28 29]. To facilitate convergence of the imputation model, costs were imputed using aggregated cost categories (medications, pharmacy reviews, secondary care, primary care, social care and other types of care) rather than at the level of individual resource-use items.

Economic analyses

Statistical analyses were conducted using Stata 14.2[30]. All analyses were by 'intention to treat' (comparing the two groups as randomised and including all patients in the primary analysis). A glossary of economic terms is given in Appendix 2, and more details may be found in Morris *et al*[31].

For peer review only - http://bmjopen.bmj.com/site/about/guidelines.xhtml

A cost–utility analysis was conducted from the NHS/PSS perspective corresponding to the NICE reference case[26]. The incremental mean difference in QALYs between the two arms of the trial and 95% confidence intervals were derived. Overall mean NHS/PSS costs, and standard errors for both arms of the trial were calculated. The incremental mean difference in total costs between the two arms of the trial and 95% confidence intervals were estimated.

Cost and QALY data were combined to calculate an incremental cost-effectiveness ratio (ICER) and net monetary benefit (NMB) statistic[32] from the NHS/PSS perspective. In the primary analysis we estimated whether the 3D approach was cost-effective at the established NICE thresholds of £20,000 and £30,000 per QALY gained. The probability that the 3D approach was cost-effective at various societal 'willingness to pay for a QALY' thresholds was depicted using a cost-effectiveness acceptability curve (CEAC). All measures of cost-effectiveness (ICER, CEAC and NMB) and confidence intervals were derived parametrically using the output of seemingly-unrelated regression analysis to account for the correlation between costs and outcomes, and controlling for baseline imbalance in utility for the QALY equation[33]. Clustering within GP practices was accounted for by including the randomisation variables in the regression.

As the broader societal perspective also has relevance, the costs of each component of the intervention were estimated separately from the NHS/PSS perspective (i.e. including all resources supplied by the healthcare provider), the patient/carer perspective (i.e. including all costs borne by the participants themselves) and the productivity perspective (i.e. taking into account time off work) and related to changes in a range of secondary outcomes in a cost–consequences analysis[34]. Consequences included QALYs accrued by both participants and carers, and deaths. The cost-consequences analysis was based on available cases, which differed in number for each type of healthcare resource or outcome; an available case was defined as an individual having complete data for each relevant timepoint. Linear regression output was used to derive confidence intervals parametrically, accounting for clustering within practices.

Sensitivity analyses

One-way sensitivity analyses were used to judge the potential impact of sources of uncertainty. This included an analysis excluding participants who died to assess the impact of the imbalance in deaths between arms, and an analysis without discounting either costs or outcomes (i.e. a discount rate of zero) to assess the impact of the discount rate. A complete-case analysis was also conducted to assess the impact of the imputation process; a complete case was defined as a participant for whom full resource-use data and full outcome data were available.

For peer review only - http://bmjopen.bmj.com/site/about/guidelines.xhtml

RESULTS

The results are reported according to the specifications of the Consolidated Health Economic Evaluation Reporting Standards (CHEERS) statement[35]. Baseline characteristics of the practices and patients are given in Table 1[13], and the flow of patients through the study is illustrated in Figure 1[13].

Missing data

16 practices (797 participants) were randomised to be offered the 3D approach, while 17 practices (749 participants) were randomised to receive usual care. Missing data occurred for a number of reasons, including withdrawal from the trial, or leaving the practice. 12 participants (1.5%) in the 3D arm and six (0.8%) in the control arm had no information on secondary care use because it was not possible to locate their medical records (p=0.2). Practice downloads of medication and investigation data failed for 18 participants (2.3%) in the intervention arm and eight (1.1%) in the usual care arm (p=0.07), while 19 (2.4%) and 10 (1.3%) 3D and usual care participants respectively were missing consultation data from practice downloads (p=0.13). Not all participants returned all questionnaires at all timepoints. 165 participants (20.7%) in the intervention arm and 125 (16.7%) in the control arm did not return a questionnaire at one or more follow-up points (p=0.04). Not all those who did return questionnaires completed the resource-use questions; in total, 181 (22.7%) in the intervention arm and 146 (19.5%) in the control arm were missing resource-use data from questionnaires at one or more follow-up points (p=0.12). Complete datasets were available for 1191 participants in total (599 (75.2%) in the 3D arm and 592 (79%) in the control arm, p=0.07). Participants with missing data were in a significantly poorer health state at baseline (mean (95%CI) EQ-5D-5L score: 0.453 (0.422 to 0.485)) than participants with full datasets (mean (95%CI) EQ-5D-5L score: 0.589 (0.574 to 0.605)).

Primary analysis

Outcomes and resource use

The primary analysis using imputed data showed that participants in the intervention arm gained a mean of 0.007 (95%CI: -0.009 to 0.023) additional QALYs over 15 months compared with participants in the usual care arm. Total costs per patient from the NHS/PSS perspective were £126 (95%CI: -£739 to £991) higher in the intervention arm than in the usual care arm. Wide variability was observed in both costs and outcomes, with both confidence intervals overlapping zero. Disaggregated resource-use data are in Appendix 3[14].

Cost-effectiveness of 3D

Cost-effectiveness statistics from the NHS/PSS perspective are given in Table 2[14]. The ICER was £18,499, and the net monetary benefit at a societal willingness-to-pay value of £20,000 was £10 (95%CI: -£956 to £977). At this willingness-to-pay value, the probability that the 3D approach is cost-effective was

For peer review only - http://bmjopen.bmj.com/site/about/guidelines.xhtml

0.508, while at £30,000, the probability of cost-effectiveness was 0.558. A cost-effectiveness acceptability curve depicting the probability of cost-effectiveness at a range of willingness-to-pay values is given in Figure 2[14].

Sensitivity analyses

Results from an analysis restricted to complete cases only are given in Table 3[14]. The complete-case analysis suggested that the 3D approach was associated with both lower costs and better outcomes, with a probability of cost-effectiveness of 0.705 at a willingness-to-pay threshold of £20,000. A sensitivity analysis excluding participants who died suggested that the probability of cost-effectiveness of the 3D approach at £20,000 was 0.561. A further sensitivity analysis using undiscounted costs and outcomes did not suggest that the discount rate affected the conclusions.

Cost–consequences analysis

Costs and selected outcomes (on an available case basis) are presented in Table 4[14] from the primary perspective of the NHS/PSS and the secondary perspective of the patient/carer themselves alongside an estimate of the societal loss of productivity.

Costs from all perspectives were very similar between arms and no cost group differed significantly (other than those associated with the intervention itself). Other than for daycase/outpatient care, emergency care and medications, costs to the NHS were higher in the intervention arm than in the usual care arm, while social services usage was higher in the usual care arm. Overall costs (based on available data) from the NHS/PSS perspective were slightly higher in the usual care arm although the difference was consistent with a chance finding.

Costs borne by patients and carers were higher overall in the intervention arm, although the medication costs (both prescription charges and over-the-counter remedies) were slightly lower; no patient cost group exhibited a statistically significant difference. The cost of productivity losses was similar in the two arms (and statistically consistent with chance), although slightly higher in the 3D approach arm.

QALYs (adjusted for baseline utility scores) were slightly higher for patients and lower for carers in the intervention arm than in the usual care arm; however, the difference was consistent with chance. Although there was a higher number of deaths in the intervention arm, the difference was not statistically significant.

For peer review only

For peer review only - http://bmjopen.bmj.com/site/about/guidelines.xhtml

DISCUSSION

Principal findings

No significant difference was observed between participants offered the 3D intervention and those receiving usual care only for overall costs, resource use of any category, or QALY outcomes. The participants were substantial users of healthcare, with inpatient hospital care and medications both high contributors to overall costs. A small improvement in outcome (representing an additional 2.6 days of the best imaginable health over 15 months) coupled with a small increase in costs between arms led to weak evidence for the cost-effectiveness of the 3D approach in the primary analysis from the NHS/PSS perspective. The net monetary benefit was small, but positive, indicating that society is willing to pay for the benefits that can be achieved.

Strengths and limitations

This economic evaluation was conducted alongside a large and rigorous RCT. Meticulous data collection practices allowed individual patient data to be measured for all the key cost drivers, a particularly challenging task for a patient group that has high healthcare usage. The study contributes to the body of evidence supporting the care of patients with multimorbidity. Patterns of missing resource-use data were similar between arms, and high questionnaire return rates were achieved[36]; however, the recall period for resource-use was quite long at 9 months, which may have led to patients omitting to include some resources. Medication costs were based on prescriptions issued by the GP, and it is not certain that all scripts were filled by the participant; the medication costs may, therefore, be overestimated. Although the medication data were checked carefully, the volume of medications prescribed to the 3D population rendered it infeasible to check every entry and some errors may have persisted.

Use of care homes, which can be a significant contributor to costs of social care, was not included in the economic evaluation. The funding of care homes within the UK is complex, with patients often paying considerable amounts themselves. However, only 13 participants reported living in a care home and this did not differ by arm. The follow-up period of the 3D trial was 15 months, and longer-term outcomes are unknown; a follow-up period of 2–3 years might have allowed an effect on quality of life or healthcare resource use to be observed. Use of simple mean imputation methods for estimating missing information (such as the cost of a bus fare) in the questionnaire data will have reduced standard errors and underestimated the uncertainty around these costs.

Comparison with other studies

Comparison with other studies is hampered by the variety of definitions used for multimorbidity, and by the heterogeneity in study designs[11]. Evidence concerning the cost-effectiveness of interventions designed to manage patients with multimorbidity is limited; only two of the RCTs identified in the review

For peer review only - http://bmjopen.bmj.com/site/about/guidelines.xhtml

by Smith *et al* undertook cost-effectiveness analyses[7]. Katon *et al* reported that implementation of a collaborative care intervention for patients with depression alongside either diabetes or coronary heart disease was justified on cost-effectiveness grounds[37]. However, the authors estimated QALYs based on clinical outcomes rather than by direct measurement, and there was substantial uncertainty around both costs and outcomes. Barley *et al* found that a nurse-led patient-centred intervention was cost-effective up to a willingness-to-pay threshold of approximately £3000 per QALY; however, the study was only a pilot RCT, and multimorbidity was defined to include patients with coronary heart disease and depression only[38]. In a feasibility trial, Mercer *et al* found that a whole-system intervention designed for patients with two or more chronic conditions in deprived areas may be considered cost-effective[39]. There is a clear need for further well conducted studies on the cost-effectiveness of interventions for multimorbid patients.

Implications of study

This is the largest trial of an intervention for multimorbidity ever undertaken; however, there remains substantial uncertainty surrounding the results. The impact of organisational changes in delivery of primary health care on patients' quality of life is usually very small and the costs are also usually low, but skewed by a small number of patients with high costs in secondary care often unrelated to the intervention. This leads to wide confidence intervals that cross zero (i.e. substantial uncertainty) for estimates of cost-effectiveness and illustrates the difficulty of obtaining precise estimates for the economic outcomes of this type of intervention even in large trials.

The 3D participants had a mean(sd) utility of 0.558(0.287) at entry to the study, which compares poorly to a UK population mean of 0.779 for ages 65-74[40]. Participants had a small positive increase in QALYs in the intervention arm, while carers had a small decrease in QALYs compared with the usual care arm; it is possible that an analysis taking carer outcomes into account might reach a different conclusion. The small contribution to overall costs made by productivity losses is consistent with the predominantly retired study population; at baseline, over 65% of participants described themselves as 'fully retired from work'.

Anticipated reductions in appointment attendances were not achieved, partly because some patients attended 3D reviews in addition to, rather than instead of, their single-condition reviews. Similarly, although cost savings could have been achieved through a reduction in prescriptions issued, this was not observed in the trial; although the brief was to seek to simplify patients' medication regimes, the embedded process evaluation[14] suggested that pharmacists appeared to concentrate on adherence to guidelines rather than whether the patient might benefit from stopping some medications. There was substantial variation between practices in the extent to which they implemented the variation and this may have contributed to variation in costs in the trial arm. Three practices barely implemented the intervention at all because of practice or staffing problems unconnected with the intervention, and this will have diluted the potential effects of the intervention overall. It is possible that the cost-effectiveness of the intervention

For peer review only - http://bmjopen.bmj.com/site/about/guidelines.xhtml

could be increased by focusing more on patients with the greatest needs, by more attention to de-prescribing, and by ending unnecessary additional disease-specific reviews as the 3D model became normalised.

Future work

The EQ-5D instrument was used because it is recommended by NICE in the UK[26], and there was little evidence on alternatives at the trial outset. However, it is difficult to pick up small changes in health-related quality of life arising from service reconfigurations, and it may be that a measure capturing outcomes beyond health would be more appropriate. In future work, researchers should consider including alternative economic outcome measures alongside the EQ-5D; for example, the ICECAP suite of capability wellbeing instruments[41]. The effect of the intervention on carers is being considered further, and it would be interesting to explore the reasons for the errors observed in the medication records downloaded from GP practice systems.

CONCLUSIONS

The evidence for the cost-effectiveness of the 3D intervention is equivocal; the results suggest that there is just over a 50% chance of cost-effectiveness at the established threshold of £20,000 per QALY from the NHS/PSS perspective. The small differences in costs and outcomes are consistent with chance, and the uncertainty is substantial; therefore, the results should be interpreted with caution. Given the equivocal nature of the cost-effectiveness results, implementation of the intervention cannot be recommended on economic grounds alone.

For peer review only - http://bmjopen.bmj.com/site/about/guidelines.xhtml

Table 1. Baseline characteristics of practices and patients.

	Usual care (n=17 practices, 749 patients)	Intervention (n=16 practices, 797 patients)
**Patients**		
Age: mean (SD)	70·7 (11·4)	71·0 (11·6)
Female: no. (%)	377 (50%)	406 (51%)
White ethnicity: no./total no. (%)	729/739 (99%)	775/780 (99%)
Fully retired from work: no./total no. (%)	512/721 (71%)	525/759 (69%)
No. of long term conditions from QOF: median (IQR)	3·0 (3·0, 3·0)	3·0 (3·0, 3·0)
No. of self-reported conditions: median (IQR), n	7·0 (5·0, 10·0), 748	7·0 (5·0, 9·0), 795
Long term conditions.[b] No. (%) with:		
Cardiovascular disease or chronic kidney disease[c]	698 (93%)	747 (94%)
Stroke or Transient Ischemic Attack	241 (32%)	286 (36%)
Diabetes	401 (54%)	411 (52%)
Chronic Obstructive Pulmonary Disease or Asthma	382 (51%)	388 (49%)
Epilepsy	35 (5%)	41 (5%)
Atrial fibrillation	249 (33%)	281 (35%)
Serious mental illness[d]	37 (5%)	29 (4%)
Depression	283 (38%)	276 (35%)
Dementia	27 (4%)	33 (4%)
Learning disability	7 (1%)	7 (1%)
Rheumatoid arthritis	55 (7%)	48 (6%)
EQ-5D-5L score: mean (SD), n	0·542 (0·292), 747	0·574 (0·282), 795

For peer review only

Table 2. Cost-effectiveness of the 3D approach from an NHS and personal social services perspective

	**Usual care Mean (SE) N=749**	**Intervention Mean (SE) N=797**	**Incremental difference (95% CI)**
**Costs (£)**			
Unadjusted costs from the NHS/PSS perspective	6032 (362)	6124 (317)	
Adjusted costs from the NHS/PSS perspective	6014 (343)	6140 (333)	126 (-739 to 991)
**Outcomes**			
Unadjusted QALYs over 15 months of follow-up	0.651 (0.013)	0.691 (0.012)	
Adjusted QALYs over 15 months of follow-up	0.668 (0.006)	0.675 (0.006)	0.007 (-0.009 to 0.023)
**Cost-effectiveness statistics**			
ICER: £18499			
Net monetary benefit at £20,000 (95%CI): £10 (-956 to 977)			
Net monetary benefit at £30,000 (95%CI): £78 (-974 to 1130)			

ICER, incremental cost-effectiveness ratio; CI, confidence interval; PSS, personal social services; QALY, quality-adjusted life year; SE, standard error. Cost estimates were adjusted for randomisation variables; QALY estimates were adjusted for randomisation variables and baseline utility.

For peer review only - http://bmjopen.bmj.com/site/about/guidelines.xhtml

Table 3. Sensitivity analysis: cost-effectiveness of the 3D approach from an NHS/PSS perspective based on complete cases only

	**Usual care Mean (SE) N=592 complete cases**	**Intervention Mean (SE) N=599 complete cases**	**Incremental difference (95%CI)**
Costs (£)			
Unadjusted costs from the NHS/PSS perspective	4916 (290)	4757 (222)	
Adjusted costs from the NHS/PSS perspective	4905 (258)	4768 (256)	-137 (-852 to 577)
Outcomes			
Unadjusted QALYs over 15 months of follow-up	0.698 (0.014)	0.750 (0.013)	
Adjusted QALYs over 15 months of follow-up	0.722 (0.005)	0.726 (0.005)	0.004 (-0.010 to 0.019)
Cost-effectiveness statistics			
ICER: Intervention dominates			
Net monetary benefit at £20,000: £222 (-584 to 1028)			

ICER, incremental cost-effectiveness ratio; CI, confidence interval; PSS, personal social services; QALY, quality-adjusted life year; SE, standard error. Cost estimates were adjusted for randomisation variables; QALY estimates were adjusted for randomisation variables and baseline utility.

For peer review only

For peer review only - http://bmjopen.bmj.com/site/about/guidelines.xhtml

Table 4. Costs and consequences of the 3D approach and usual care

Costs and outcomes	Usual care	*N*	Intervention	*N*	Difference (95% CI)
**Mean costs from the NHS perspective (£)**					
Practice-based consultations	627	754	726	715	99 (-7 to 205)
Practice-based investigations	45	755	61	717	15 (-6 to 37)
Community-based healthcare	160	615	167	601	7 (-35 to 49)
Inpatient stays	1867	766	1920	722	52 (-470 to 574)
Outpatient visits and day cases	614	766	613	722	-1 (-168 to 167)
Accident and emergency visits	102	766	99	722	-3 (-24 to 19)
Ambulance trips to hospital	131	615	141	601	10 (-56 to 77)
Prescribed medications	1230	755	1221	717	-8 (-220 to 203)
Pharmacy reviews	0	766	8	722	8 (7 to 9)
Intervention set up	0	797	4	749	4 (3 to 5)
Social services	559	615	403	601	-156 (-476 to 164)
All NHS/PSS	4929	609	4746	598	-183 (-923 to 556)
**Mean costs from the patient/carer perspective (£)**					
Prescription charges	5	755	4	717	-2 (-6 to 2)
Travel to GP practice	24	749	34	711	10 (-4 to 24)
Over-the-counter medications	39	615	35	601	-3 (-16 to 9)
Private healthcare	93	615	122	601	29 (-40 to 97)
All patient/carer	162	608	195	597	33 (-35 to 101)
**Mean productivity loss (£)**					
Productivity loss	122	608	161	597	39 (-47 to 125)
**Outcomes**					
QALYs (patient)	0.693	665	0.695	647	0.003 (-0.013 to 0.019)
QALYs (carer)	0.943	41	0.920	50	-0.024 (-0.064 to 0.017)
Deaths	32	749	46	797	p=0.18 (χ^2 test)

CI, confidence interval; QALY, quality-adjusted life year; PSS, personal social services. All costs and consequences are based on available data; the totals from each perspective are not, therefore, equal to the sum of the components. CI were calculated using standard errors from standard linear regressions adjusted for cluster at the level of the practice. QALYs were adjusted for baseline utility scores.

For peer review only - http://bmjopen.bmj.com/site/about/guidelines.xhtml

References

1. National Institute for Health and Care Excellence. Multimorbidity: clinical assessment and management 2016 [Available from: https://www.nice.org.uk/guidance/ng56.
2. Peters M, Kelly L, Potter CM, et al. Quality of life and burden of morbidity in primary care users with multimorbidity. *Patient Related Outcome Measures* 2018;9:103.
3. Li J, Green M, Kearns B, et al. Patterns of multimorbidity and their association with health outcomes within Yorkshire, England: baseline results from the Yorkshire Health Study. *BMC public health* 2016;16(1):649.
4. Marengoni A, Angleman S, Melis R, et al. Aging with multimorbidity: a systematic review of the literature. *Ageing research reviews* 2011;10(4):430-39.
5. Kasteridis P, Street A, Dolman M, et al. The importance of multimorbidity in explaining utilisation and costs across health and social care settings: evidence from South Somersets Symphony Project, 2014.
6. Wang L, Si L, Cocker F, et al. A Systematic Review of Cost-of-Illness Studies of Multimorbidity. *Applied health economics and health policy* 2017:1-15.
7. Smith SM, Wallace E, O'Dowd T, et al. Interventions for improving outcomes in patients with multimorbidity in primary care and community settings. *Cochrane Database of Systematic Reviews* 2016(3) doi: 10.1002/14651858.CD006560.pub3
8. Navickas R, Petric V-K, Feigl AB, et al. Multimorbidity: What do we know? What should we do? *Journal of comorbidity* 2016;6(1):4.
9. McPhail SM. Multimorbidity in chronic disease: impact on health care resources and costs. *Risk management and healthcare policy* 2016;9:143.
10. Hopman P, De Bruin SR, Forjaz MJ, et al. Effectiveness of comprehensive care programs for patients with multiple chronic conditions or frailty: a systematic literature review. *Health Policy* 2016;120(7):818-32.
11. Xu X, Mishra GD, Jones M. Evidence on multimorbidity from definition to intervention: an overview of systematic reviews. *Ageing research reviews* 2017;37:53-68.
12. Stewart M. Towards a global definition of patient centred care: the patient should be the judge of patient centred care: British Medical Journal Publishing Group, 2001.
13. Salisbury C, Man M-S, Bower P, et al. Management of multimorbidity using a patient-centred care model: a pragmatic cluster-randomised trial of the 3D approach. *The Lancet* 2018;392(10141):41-50.
14. Salisbury C, Man M-S, Chaplin K, et al. A patient-centred intervention to improve the management of multimorbidity in general practice: the 3D RCT. 2019;7:5. doi: 10.3310/hsdr07050
15. Man M-S, Chaplin K, Mann C, et al. Improving the management of multimorbidity in general practice: protocol of a cluster randomised controlled trial (The 3D Study). *BMJ open* 2016;6(4):e011261.
16. NHS Employers. Quality and outcomes framework [Available from: http://www.nhsemployers.org/your-workforce/primary-care-contacts/general-medical-services/quality-and-outcomes-framework accessed August 2018.
17. Herdman M, Gudex C, Lloyd A, et al. Development and preliminary testing of the new five-level version of EQ-5D (EQ-5D-5L). *Quality of life research* 2011;20(10):1727-36.
18. Van Hout B, Janssen M, Feng Y-S, et al. Interim scoring for the EQ-5D-5L: mapping the EQ-5D-5L to EQ-5D-3L value sets. *Value in Health* 2012;15(5):708-15.
19. National Institute for Health and Care Excellence. Position statement on use of the EQ-5D-5L valuation set 2017 [Available from: https://www.nice.org.uk/Media/Default/About/what-we-do/NICE-guidance/NICE-technology-appraisal-guidance/eq5d5l_nice_position_statement.pdf.

For peer review only - http://bmjopen.bmj.com/site/about/guidelines.xhtml

20. Curtis L, Burns A. Unit Costs of Health and Social Care 2016. University of Kent, Canterbury: Personal Social Services Research Unit 2016.
21. British National Formulary, March-September 2016. Basingstoke: Pharmaceutical Press/BMJ Group 2016.
22. Department of Health. NHS reference costs 2015 to 2016 2016 [Available from: https://www.gov.uk/government/publications/nhs-reference-costs-2015-to-2016.
23. Office for National Statistics. Annual Survey of Hours and Earnings: 2016 2016 [Available from: https://www.ons.gov.uk/employmentandlabourmarket/peopleinwork/earningsandworkinghours/datasets/agegroupashetable6.
24. HM Revenue and Customs. Travel - mileage and fuel rates and allowances Updated 6 April 2017 [Available from: https://www.gov.uk/government/publications/rates-and-allowances-travel-mileage-and-fuel-allowances/travel-mileage-and-fuel-rates-and-allowances.
25. Bank of England. UK inflation calculator [Available from: http://www.bankofengland.co.uk/education/Pages/resources/inflationtools/calculator/default.aspx.
26. NICE. Guide to the methods of technology appraisal 2013 2013 [Available from: https://www.nice.org.uk/process/pmg9/chapter/1-foreword.
27. Sterne JA, White IR, Carlin JB, et al. Multiple imputation for missing data in epidemiological and clinical research: potential and pitfalls. *Bmj* 2009;338:b2393.
28. Gomes M, Diaz-Ordaz K, Grieve R, et al. Multiple imputation methods for handling missing data in cost-effectiveness analyses that use data from hierarchical studies: an application to cluster randomized trials. *Med Decis Making* 2013;33(8):1051-63.
29. Díaz-Ordaz K, Kenward MG, Grieve R. Handling missing values in cost effectiveness analyses that use data from cluster randomized trials. *Journal of the Royal Statistical Society: Series A (Statistics in Society)* 2014;177(2):457-74. doi: 10.1111/rssa.12016
30. Stata Statistical Software: Release 14 [program]. College Station, TX: StataCorp LP., 2015.
31. Morris S, Devlin N, Parkin D. Economic analysis in health care. Chichester: John Wiley & Sons 2007.
32. Löthgren M, Zethraeus N. Definition, interpretation and calculation of cost-effectiveness acceptability curves. *Health Econ* 2000;9(7):623-30.
33. Manca A, Hawkins N, Sculpher MJ. Estimating mean QALYs in trial-based cost-effectiveness analysis: the importance of controlling for baseline utility. *Health Econ* 2005;14(5):487-96.
34. Mauskopf JA, Paul JE, Grant DM, et al. The Role of Cost—Consequence Analysis in Healthcare Decision—Making. *Pharmacoeconomics* 1998;13(3):277-88.
35. Husereau D, Drummond M, Petrou S, et al. Consolidated health economic evaluation reporting standards (CHEERS) statement. *Cost Effectiveness and Resource Allocation* 2013;11(1):6.
36. Noble SM, Hollingworth W, Tilling K. Missing data in trial-based cost-effectiveness analysis: the current state of play. *Health economics* 2012;21(2):187-200.
37. Katon W, Russo J, Lin EH, et al. Cost-effectiveness of a multicondition collaborative care intervention: a randomized controlled trial. *Archives of general psychiatry* 2012;69(5):506-14.
38. Barley EA, Walters P, Haddad M, et al. The UPBEAT nurse-delivered personalized care intervention for people with coronary heart disease who report current chest pain and depression: a randomised controlled pilot study. *PLoS One* 2014;9(6):e98704.
39. Mercer SW, Fitzpatrick B, Guthrie B, et al. The CARE Plus study–a whole-system intervention to improve quality of life of primary care patients with multimorbidity in areas of high socioeconomic deprivation: exploratory cluster randomised controlled trial and cost-utility analysis. *BMC medicine* 2016;14(1):88.
40. Janssen B, Szende A. Population norms for the EQ-5D. Self-reported population health: An international perspective based on EQ-5D: Springer 2014:19-30.

For peer review only - http://bmjopen.bmj.com/site/about/guidelines.xhtml

41. Al-Janabi H, Flynn TN, Coast J. Development of a self-report measure of capability wellbeing for adults: the ICECAP-A. *Quality of Life Research* 2012;21(1):167-76.

Figure captions

Figure 1. Trial profile †

† Salisbury C, Man M-S, Bower P, et al. Management of multimorbidity using a patient-centred care model: a pragmatic cluster-randomised trial of the 3D approach. *The Lancet* 2018;392(10141):41-50

Figure 2. Cost-effectiveness acceptability curve from the NHS/PSS perspective.†

† Salisbury C, Man M-S, Chaplin K, Mann C, Bower P, Brookes S, et al. A patient-centred intervention to improve the management of multimorbidity in general practice: the 3D RCT. Health Serv Deliv Res 2019;7(5)

For peer review only

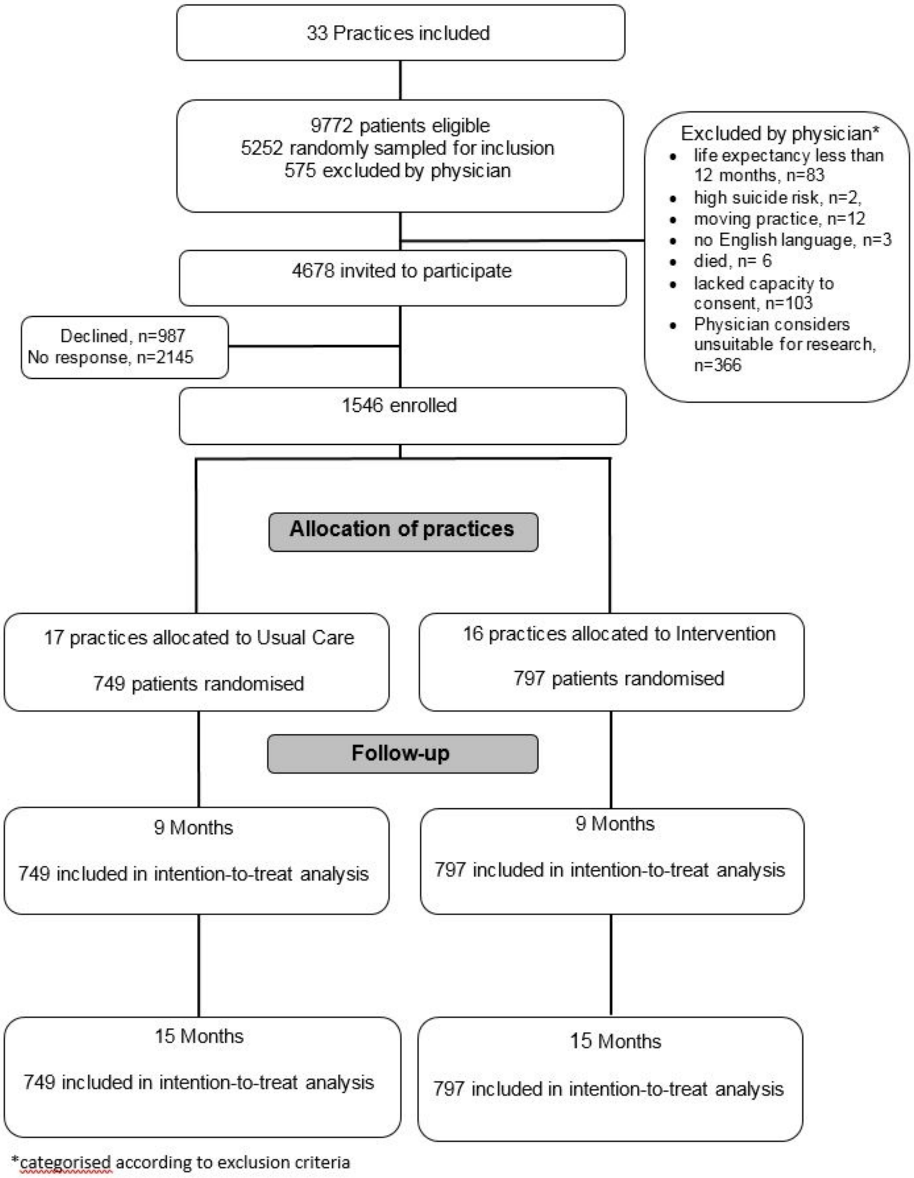

Figure 1. Trial profile †

† Salisbury C, Man M-S, Bower P, et al. Management of multimorbidity using a patient-centred care model: a pragmatic cluster-randomised trial of the 3D approach. The Lancet 2018;392(10141):41-50

190x240mm (96 x 96 DPI)

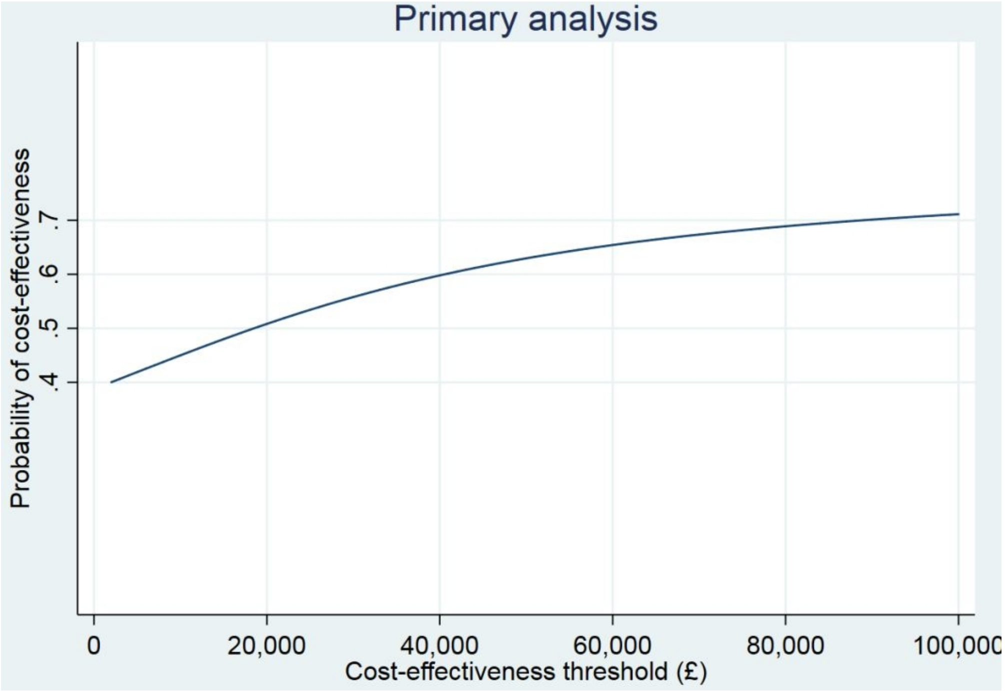

Figure 2. Cost-effectiveness acceptability curve from the NHS/PSS perspective.†† Salisbury C, Man M-S, Chaplin K, Mann C, Bower P, Brookes S, et al. A patient-centred intervention to improve the management of multimorbidity in general practice: the 3D RCT. Health Serv Deliv Res 2019;7(5)

90x61mm (300 x 300 DPI)

Appendix 1. Unit costs

Service		Unit cost (£)	Notes	Source
**Social services**				
Social home care provided for social services		12	Independent sector home care provided for social services, face-to-face, based on 30 minute visit	[1]
Daycare		61	Per client attendance	
Social worker		55	Based on 1 hour appointment	
Meals on wheels		7.03		[2]
**Prescription charges***				
Prescription charge per item		8.40		[3]
Prescription charge 3mo prepayment certificate		29.10		
Prescription charge 12mo prepayment certificate		104		
**GP practice services**				
GP appointment	Control arm	51.84	Per surgery consultation lasting 14.4 minutes (3D data)	[1]
	Intervention arm	48.60	Per surgery consultation lasting 13.5 minutes (3D data)	

For peer review only

1

Service		Unit cost (£)	Notes	Source
GP home visit		100	Based on consultation lasting 15 minutes and 12 minutes travel time[4], plus 5 miles travel cost (NHS travel reimbursed at 56p per mile)	
GP phone call	Control arm	41.40	Based on consultation lasting 11.5 minutes (3D data)	
	Intervention arm	32.40	Based on consultation lasting 9 minutes (3D data)	
GP email consultation		21.60	Based on 6 minute consultation[5]	
Nurse appointment	Control arm	10.85	Based on 15.1 minute appointment (3D data), inflated from 14/15 costs[6]	
	Intervention arm	14.16	Based on 19.7 minute appointment (3D data), inflated from 14/15 costs[6]	
Nurse phone call	Control arm	4.24	Based on 5.9 minute call, inflated from 14/15 costs [6]	
	Intervention arm	5.03	Based on 7 minute call, inflated from 14/15 costs[6]	
Nurse home visit		22.21	Based on 15 minute visit, 12 minutes travelling, 5 miles travel costs (NHS travel reimbursed at 56p per mile)	
HCA face-to-face appointment	Control arm	3.45	Based on 9.4 minute appointment (3D data), band[**] 2 nurse	
	Intervention arm	3.59	Based on 9.8 minute appointment (3D data), band 2 nurse	
HCA phone call (control arm)		5.87	Based on 16 minute call (3D data), band 2 nurse	

2

For peer review only

Service	Unit cost (£)	Notes	Source
HCA home visit	12.70	Based on 15 minutes appointment, 12 minutes travelling, 5 miles travel costs, band 2 nurse (NHS travel reimbursed at 56p per mile)	
**Community services**			
Occupational Therapy visit	16	Based on band 5 occupational therapist, 30 minute appointment	[1]
Occupational Therapy home visit	25.20	Based on band 5 occupational therapist, 30 minute appointment, 12 minutes travelling, 5 miles travel costs (NHS travel reimbursed at 56p per mile)	
Occupational Therapy phone call	5.33	Based on band 5 occupational therapist, 10 minute call	
Speech and Language Therapy (SALT) visit	16	Based on band 5 SALT, 30 minute appointment	
Speech and Language Therapy home visit	25.20	Based on band 5 SALT, 30 minute appointment, 12 minutes travelling, 5 miles travel costs (NHS travel reimbursed at 56p per mile)	
Speech and Language Therapy telephone call	5.33	Based on band 5 SALT, 10 minute call	

For peer review only - http://bmjopen.bmj.com/site/about/guidelines.xhtml

Service	Unit cost (£)	Notes	Source
Physiotherapy visit	16	Based on band 5 physiotherapist, 30 minute appointment	
Physiotherapy home visit	25.20	Based on band 5 physiotherapist, 30 minute appointment, 12 minutes travelling, 5 miles travel costs (NHS travel reimbursed at 56p per mile)	
Physiotherapy phone call	5.33	Based on band 5 physiotherapist, 10 minute call	
Chiropody / Podiatry visit	16	Based on band 5 podiatrist, 30 minute appointment	
Chiropody / Podiatry home visit	25.20	Based on band 5 podiatrist, 30 minute appointment, 12 minutes travelling, 5 miles travel costs (NHS travel reimbursed at 56p per mile)	
Chiropody / Podiatry phone call	5.33	Based on band 5 podiatrist, 10 minute call	
Community / District Nurse /Mental health nurse visit	22	Based on band 6 district nurse, 30 minute appointment	
Community / District Nurse /Mental health nurse home visit	46.8	Based on band 6 district nurse, 1 hour appointment including travel time, 5 miles travel costs (NHS travel reimbursed at 56p per mile)	
Community / District Nurse/ Mental health nurse phone call	7.33	Based on band 6 district nurse, 10 minute phone call	

For peer review only - http://bmjopen.bmj.com/site/about/guidelines.xhtml

Service	Unit cost (£)	Notes	Source
NHS Counselling/Cognitive Behavioural Therapy visit	21	Based on band 6 counsellor, 30 minute appointment	
NHS Counselling/Cognitive Behavioural Therapy home visit	32.20	Based on band 6 counsellor, 30 minutes appointment, 12 minutes travelling, 5 miles travel costs (NHS travel reimbursed at 56p per mile)	
NHS Counselling/Cognitive Behavioural Therapy phone call	7	Based on band 6 counsellor, 10 minute call	
NHS 111 phone call	8.06	Maximum call cost of £7.80 in May 2013	[7]
NHS walk-in centre visit	41.74	Weighted mean of type 4 non-admitted, emergency medicine per attendance	[8]
OOH service visit	82.32	Average OOH hourly evening rate was £58.36 in 2005 compared with £36.75 for normal hours. Same differential applied to current GP in hours costs.	[9,10]
OOH service home visit	158.80		
OOH service phone call	65.74		
**Ambulance services**			
Paramedic at home not involving a hospital visit	184	Ambulance services, see, treat and refer	[1]
Ambulance to hospital	238	See and treat and convey (including carbon 39 kgCO2e)	[1]
**Investigations (in outpatients)**			

5

For peer review only

Service	Unit cost (£)	Notes	Source
MRI	155.64		8
DEXA	71		
CT scan	104.88		
ECG	72		
Ultrasound	58.47		
Fluoroscopy	147.68		
**Investigations (directly accessed)**			
Cytology	16.88	Directly accessed pathology services	8
Histopathology and histology	30.77	Directly accessed pathology services	
Clinical Biochemistry	1.18	Directly accessed pathology services	
Haematology	3.10	Directly accessed pathology services	
Immunology	6.42	Directly accessed pathology services	
Microbiology	7.63	Directly accessed pathology services	
Other	3.13	Directly accessed pathology services	
MRI scan	147.25	Diagnostic imaging, directly accessed, weighted mean	
CT scan	107.52	Diagnostic imaging, directly accessed, weighted mean	
Ultrasound scan	52.82	Diagnostic imaging, directly accessed, weighted mean	
Bone scan	210.11	Nuclear medicine, directly accessed, weighted mean	

For peer review only - http://bmjopen.bmj.com/site/about/guidelines.xhtml

Service	Unit cost (£)	Notes	Source
x-ray	30.26	Directly accessed diagnostic services, Plain film	
DEXA scan	68.29	Diagnostic imaging, direct access	
ECG	67.06	Diagnostic imaging, direct access, 19 years and over	
**Secondary care**			
Outpatient attendance	135.27	Weighted mean, consultant led	[8]
Day case	733.31	Weight mean of all daycases	
Elective inpatient	3749.81	Weighted mean	
Non-elective inpatient, long stay	3058.14	Weighted mean	
Non-elective inpatient, short stay	615.83	Weighted mean	
Elective excess bed day	361.67	Weighted mean	
Non-elective excess bed day	298.41	Weighted mean	
Individual HRGs		Mapped directly from Reference Costs file	
**Productivity losses**			
Median hourly wage, age 22-29	10.52	Excluding overtime, for employees on adult wages not affected by absence	[11]
Median hourly wage, age 30-39	13.57		
Median hourly wage, age 40-49	13.92		
Median hourly wage, age 50-59	13.18		

For peer review only

Service	Unit cost (£)	Notes	Source
Median hourly wage, age 60+	11.26		

* Where prescription charges are levied, patients can choose to pay for each individual item separately, or can purchase a pre-payment certificate to cover any number of prescriptions in a 3- or 12-month period ** NHS bands refer to payscales: for example, a recently qualified nurse will be paid on band 5, while a nurse with more experience might be paid on band 6.

OOH: out-of-hours. HCA: health care assistant. MRI: magnetic resonance imaging. DEXA: dual energy X-ray absorptiometry. ECG: electrocardiogram. CT: computerized tomography.

1. Curtis L, Burns A. Unit Costs of Health and Social Care 2016. University of Kent, Canterbury: Personal Social Services Research Unit 2016.
2. Curtis L. Unit Costs of Health and Social Care 2014. University of Kent, Canterbury: Personal Social Services Research Unit 2014.
3. Department of Health. NHS charges from April 2016 2016 [Available from: https://www.gov.uk/government/speeches/nhs-charges-from-april-2016.
4. Curtis L, Burns A. Unit Costs of Health and Social Care 2015. University of Kent, Canterbury: Personal Social Services Research Unit 2015.
5. Matheson C. Implementation of WebGP and e-consultations in Wessex GP practices. 2016
6. Bank of England. UK inflation calculator [Available from: http://www.bankofengland.co.uk/education/Pages/resources/inflationtools/calculator/default.aspx.
7. Health Committee. Written evidence from NHS Direct NHS Trust (ES 31) 2013 [Available from: https://www.publications.parliament.uk/pa/cm201314/cmselect/cmhealth/171/171vw25.htm.
8. Department of Health. NHS reference costs 2015 to 2016 2016 [Available from: https://www.gov.uk/government/publications/nhs-reference-costs-2015-to-2016.
9. O'Dowd A. Cost of out of hours care was 22% higher than predicted in England. *BMJ: British Medical Journal* 2006;332(7550):1113.
10. Curtis L, Netten A. Unit costs of health and social care: University of Kent; 2005 [
11. Office for National Statistics. Annual Survey of Hours and Earnings: 2016 2016 [Available from: https://www.ons.gov.uk/employmentandlabourmarket/peopleinwork/earningsandworkinghours/datasets/agegroupashetable6.

For peer review only - http://bmjopen.bmj.com/site/about/guidelines.xhtml

Appendix 2. **Glossary of economic terms.**

Economic evaluation

Economic evaluation refers to an analysis comparing both costs and outcomes (or benefits) to assess the value for money that an intervention offers.

Cost-consequences analysis

A cost-consequences analysis (CCA) is a type of economic evaluation in which the costs and the outcomes are presented in disaggregated form. No attempt is made to combine costs and outcomes into a cost-effectiveness statistic, instead providing commissioners with a broad range of relevant information, and allowing the scope to apply their own priorities.

Cost-effectiveness analysis

A cost-effectiveness analysis (CEA) is a type of economic evaluation in which the outcomes (or benefits) are measured in terms of a single (usually) clinical measure. For example, a cost-effectiveness analysis might have a result expressed in terms of cost per life-year gained.

Cost-utility analysis

A cost-utility analysis (CUA) is a type of economic evaluation in which the outcome is assessed in terms of a measure of health outcome to which preference-based valuations have been attached. The most commonly used outcome is the quality-adjusted life year (QALY). CUA is a particular form of CEA.

Net monetary benefit

The net monetary benefit is a cost-effectiveness statistic combining costs and outcomes in a single measure, and taking into account the societal 'willingness-to-pay' for a unit of improvement. A positive incremental net monetary benefit indicates that society is willing to pay for the benefits that can be gained.

For peer review only

Appendix 3. Mean number of health and social care resource-use contacts over 15 months of follow-up.

**Services**	**Usual care**		**Intervention**	
	**mean (SD)**	***N***	**mean (SD)**	***N***
Inpatient stays	0.50 (1.05)	722	0.55 (1.09)	766
Inpatient nights	4.51 (16.6)	722	3.84 (11.94)	766
Outpatient visits	3.60 (4.00)	722	3.64 (3.73)	766
Day cases	0.15 (0.54)	722	0.14 (0.51)	766
Accident and emergency visits	0.63 (1.41)	722	0.61 (1.14)	766
Ambulance journeys	0.55 (1.83)	601	0.60 (3.50)	615
GP face-to-face consultations	7.27 (5.93)	715	8.70 (6.26)	754
GP home visits	0.64 (1.89)	715	0.79 (2.16)	754
GP phone calls	2.88 (5.00)	715	2.99 (4.53)	754
Nurse face-to-face consultations	5.40 (5.70)	715	7.93 (10.09)	754
Nurse home visits	0.06 (0.40)	715	0.09 (0.54)	754
Nurse phone calls	0.60 (1.56)	715	0.76 (1.63)	754
Healthcare assistant face-to-face consultations	2.42 (4.30)	715	3.55 (7.75)	754
Healthcare assistant GP home visits	0.00 (0.05)	715	0.08 (0.28)	754
Healthcare assistant GP phone calls	0.03 (0.17)	715	0.03 (0.17)	754
Prescribed medications	143.44 (130.57)	717	132.23 (117.36)	755
GP investigations	19.60 (16.52)	717	22.61 (16.99)	755
Pharmacist reviews		722	0.78 (0.42)	766
Occupational therapist clinic visits	0.18 (1.10)	601	0.2 (1.28)	615
Occupational therapist home visits	0.24 (1.29)	601	0.08 (0.5)	615
Occupational therapist phone calls	0.06 (0.53)	601	0.01 (0.14)	615
Speech and language therapist clinic visits	0.02 (0.33)	601	0.1 (1.03)	615
Speech and language therapist home visits	0.04 (0.69)	601	0 (0)	615
Speech and language therapist phone calls	0.00 (0.08)	601	0 (0)	615
Physiotherapist clinic visits	0.80 (2.74)	601	1.03 (2.67)	615
Physiotherapist home visits	0.16 (1.34)	601	0.15 (0.92)	615
Physiotherapist phone calls	0.00 (0.06)	601	0.01 (0.11)	615
Podiatrist clinic visits	1.26 (2.83)	601	1.19 (2.53)	615
Podiatrist home visits	0.13 (0.84)	601	0.21 (1.46)	615
Podiatrist phone calls	0.01 (0.13)	601	0.02 (0.49)	615
Community mental health nurse clinic contacts	0.27 (2.59)	601	0.08 (0.42)	615
Community mental health nurse home visits	0.21 (2.45)	601	0.35 (5.16)	615
Community mental health nurse phone calls	0.02 (0.23)	601	0.01 (0.13)	615

For peer review only

District nurse home visits	1.21 (4.08)	601	1.48 (6.54)	615
District nurse phone calls	0.03 (0.30)	601	0.05 (0.84)	615
Counsellor clinic visits	0.24 (2.34)	601	0.14 (0.99)	615
Counsellor home visits	0.01 (0.10)	601	0.01 (0.18)	615
Counsellor phone calls	0.00 (0.08)	601	0.00 (0.06)	615
NHS 111 phone calls	0.21 (0.85)	601	0.18 (0.57)	615
NHS walk-in centre visits	0.08 (0.41)	601	0.09 (0.50)	615
GP out-of-hours clinic visits	0.08 (0.34)	601	0.07 (0.28)	615
GP out-of-hours home visits	0.04 (0.33)	601	0.06 (0.56)	615
GP out-of-hours phone calls	0.02 (0.20)	601	0.03 (0.28)	615
Paramedic attendances at home	0.09 (0.43)	601	0.05 (0.29)	615
Other healthcare clinic visits	0.11 (0.88)	601	0.25 (3.24)	615
Other healthcare home visits	0.06 (0.65)	601	0.04 (0.41)	615
Other healthcare phone calls	0.01 (0.19)	601	0.01 (0.10)	615
Carer contacts	30.93 (148.26)	601	25.27 (135.78)	615
Daycare contacts	2.14 (17.53)	601	1.17 (10.50)	615
Meals on wheels	2.66 (28.32)	601	0.93 (18.98)	615
Social worker contacts	0.77 (11.35)	601	0.45 (3.98)	615

For peer review only

Reporting checklist for economic evaluation of health interventions

.

Based on the CHEERS guidelines.

Instructions to authors

Complete this checklist by entering the page numbers from your manuscript where readers will find each of the items listed below.

Your article may not currently address all the items on the checklist. Please modify your text to include the missing information. If you are certain that an item does not apply, please write "n/a" and provide a short explanation.

Upload your completed checklist as an extra file when you submit to a journal.

In your methods section, say that you used the CHEERS reporting guidelines, and cite them as:

Husereau D, Drummond M, Petrou S, Carswell C, Moher D, Greenberg D, Augustovski F, Briggs AH, Mauskopf J, Loder E. Consolidated Health Economic Evaluation Reporting Standards (CHEERS) statement.

		Reporting Item	Page Number
	#1	Identify the study as an economic evaluation or use more specific terms such as “cost-effectiveness analysis”, and describe the interventions compared.	1

For peer review only

	#2	Provide a structured summary of objectives, perspective, setting, methods (including study design and inputs), results (including base case and uncertainty analyses), and conclusions	3
Background and objectives	#3	Provide an explicit statement of the broader context for the study. Present the study question and its relevance for health policy or practice decisions	5
Target population and subgroups	#4	Describe characteristics of the base case population and subgroups analysed, including why they were chosen.	5
Setting and location	#5	State relevant aspects of the system(s) in which the decision(s) need(s) to be made.	5
Study perspective	#6	Describe the perspective of the study and relate this to the costs being evaluated.	5
Comparators	#7	Describe the interventions or strategies being compared and state why they were chosen.	5-6
Time horizon	#8	State the time horizon(s) over which costs and consequences are being evaluated and say why appropriate.	5
Discount rate	#9	Report the choice of discount rate(s) used for costs and outcomes and say why appropriate	7
Choice of health outcomes	#10	Describe what outcomes were used as the measure(s) of benefit in the evaluation and their relevance for the type of analysis performed	6

For peer review only

Meaurement of effectiveness	#11a	Single study-based estimates: Describe fully the design features of the single effectiveness study and why the single study was a sufficient source of clinical effectiveness data	5
Measurement of effectiveness	#11b	Synthesis-based estimates: Describe fully the methods used for identification of included studies and synthesis of clinical effectiveness data	n/a
Measurement and valuation of preference based outcomes	#12	If applicable, describe the population and methods used to elicit preferences for outcomes.	6
	#13a	Single study-based economic evaluation: Describe approaches used to estimate resource use associated with the alternative interventions. Describe primary or secondary research methods for valuing each resource item in terms of its unit cost. Describe any adjustments made to approximate to opportunity costs	6-7
Estimating resources and costs	#13b	Model-based economic evaluation: Describe approaches and data sources used to estimate resource use associated with model health states. Describe primary or secondary research methods for valuing each resource item in terms of its unit cost. Describe any adjustments made to approximate to opportunity costs.	n/a
Currency, price date,	#14	Report the dates of the estimated resource quantities and	7

For peer review only

and conversion		unit costs. Describe methods for adjusting estimated unit costs to the year of reported costs if necessary. Describe methods for converting costs into a common currency base and the exchange rate.	
Choice of model	#15	Describe and give reasons for the specific type of decision analytical model used. Providing a figure to show model structure is strongly recommended.	n/a
Assumptions	#16	Describe all structural or other assumptions underpinning the decision-analytical model.	n/a
Analytical methods	#17	Describe all analytical methods supporting the evaluation. This could include methods for dealing with skewed, missing, or censored data; extrapolation methods; methods for pooling data; approaches to validate or make adjustments (such as half cycle corrections) to a model; and methods for handling population heterogeneity and uncertainty.	8-9
Study parameters	#18	Report the values, ranges, references, and, if used, probability distributions for all parameters. Report reasons or sources for distributions used to represent uncertainty where appropriate. Providing a table to show the input values is strongly recommended.	n/a
Incremental costs and outcomes	#19	For each intervention, report mean values for the main categories of estimated costs and outcomes of interest, as well as mean differences between the comparator groups.	18

For peer review only

		If applicable, report incremental cost-effectiveness ratios.	
Characterising uncertainty	#20a	Single study-based economic evaluation: Describe the effects of sampling uncertainty for the estimated incremental cost and incremental effectiveness parameters, together with the impact of methodological assumptions (such as discount rate, study perspective).	10
	#20b	Model-based economic evaluation: Describe the effects on the results of uncertainty for all input parameters, and uncertainty related to the structure of the model and assumptions.	n/a
Characterising heterogeneity	#21	If applicable, report differences in costs, outcomes, or cost effectiveness that can be explained by variations between subgroups of patients with different baseline characteristics or other observed variability in effects that are not reducible by more information.	10
Study findings, limitations, generalisability, and current knowledge	#22	Summarise key study findings and describe how they support the conclusions reached. Discuss limitations and the generalisability of the findings and how the findings fit with current knowledge.	11-13
Source of funding	#23	Describe how the study was funded and the role of the funder in the identification, design, conduct, and reporting of the analysis. Describe other non-monetary sources of support	1
Conflict of interest	#24	Describe any potential for conflict of interest of study	2

For peer review only

contributors in accordance with journal policy. In the absence of a journal policy, we recommend authors comply with International Committee of Medical Journal Editors recommendations

The CHEERS checklist is distributed under the terms of the Creative Commons Attribution License CC-BY-NC. This checklist can be completed online using https://www.goodreports.org/, a tool made by the EQUATOR Network in collaboration with Penelope.ai

For peer review only
